# Supplementary figures and images for: Structural characterisation of chromatin remodelling intermediates supports linker DNA-dependent product inhibition as a mechanism for nucleosome spacing
Source: eLife. 2025 Dec 24;14:e52513. doi: 10.7554/eLife.52513 (PMC12834501; doi:10.7554/eLife.52513)

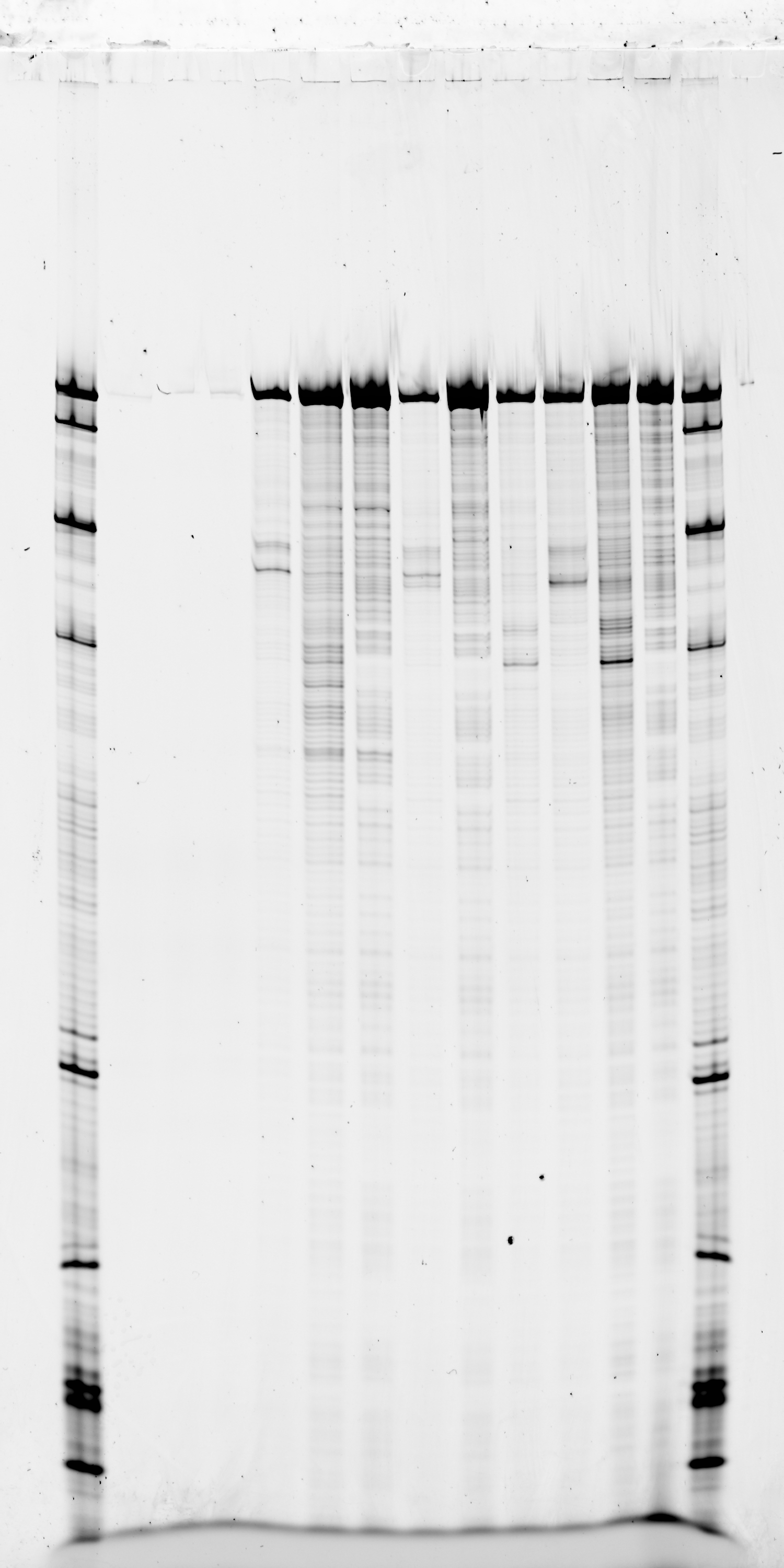

Supplement: Figure 1—source data 1. [file elife-52513-fig1-data1.zip › Figure1-sourcedata-original/NucMAPscans/19Oct2018xNucMAP0W47gap-24C-14C-4C-Cy3.tif]

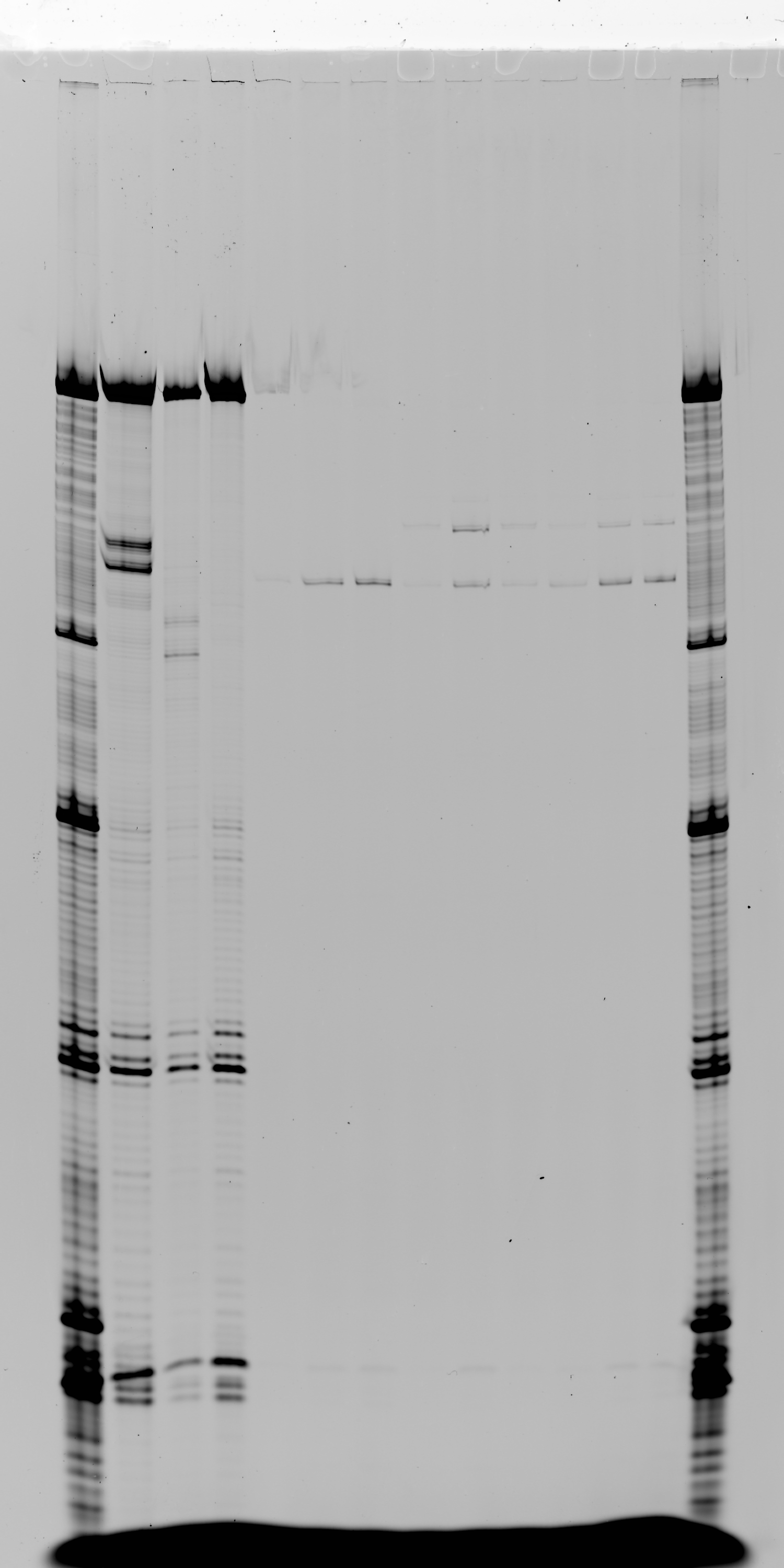

Supplement: Figure 1—source data 1. [file elife-52513-fig1-data1.zip › Figure1-sourcedata-original/NucMAPscans/19Oct2018xNucMAP0W47gap-24C-14C-4C-Cy5.tif]

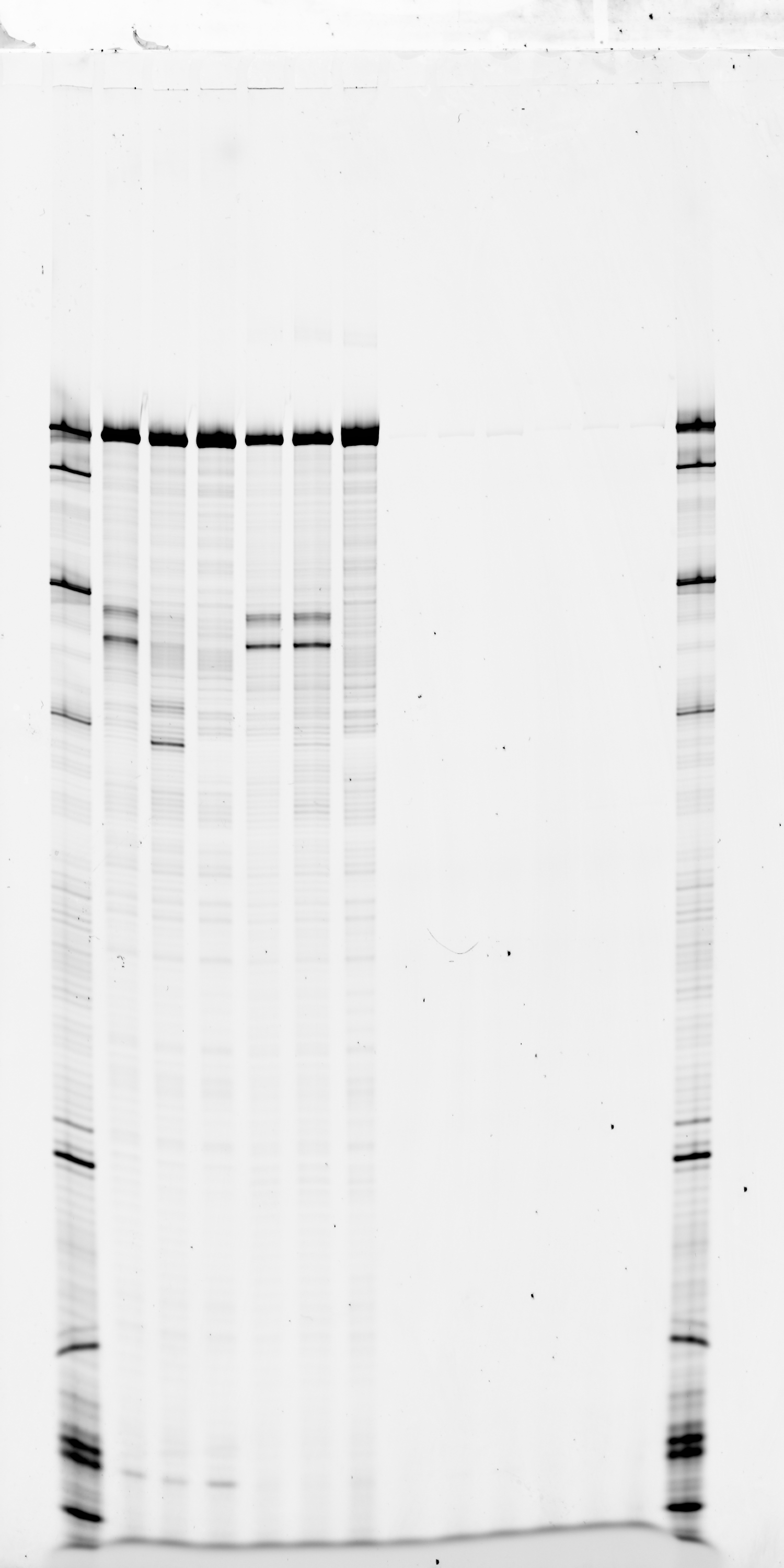

Supplement: Figure 1—source data 1. [file elife-52513-fig1-data1.zip › Figure1-sourcedata-original/NucMAPscans/19OCt2018xNucMAPgap+5C+15C+25C+35C-Cy3.tif]

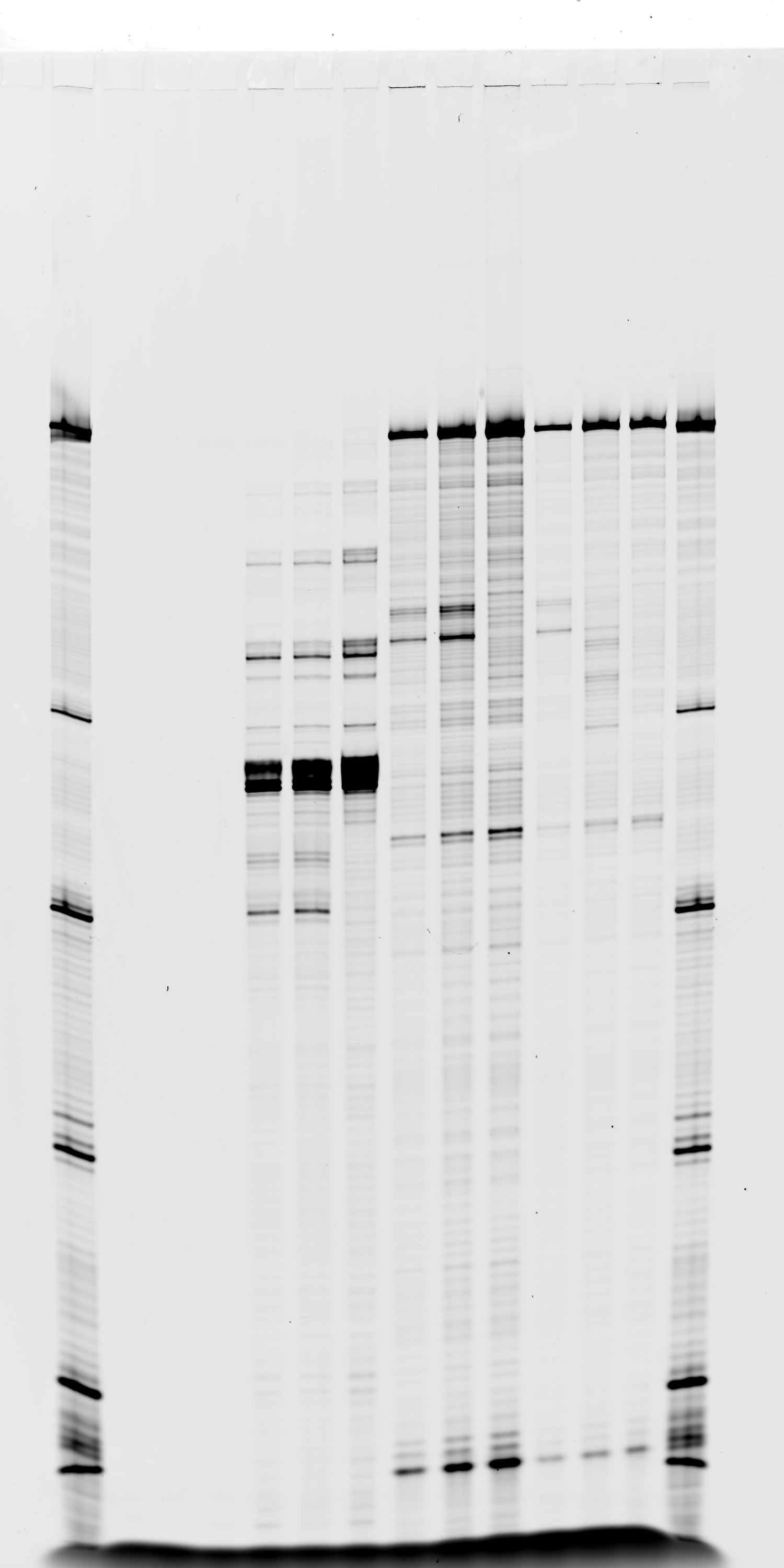

Supplement: Figure 1—source data 1. [file elife-52513-fig1-data1.zip › Figure1-sourcedata-original/NucMAPscans/19OCt2018xNucMAPgap+5C+15C+25C+35C-Cy5.tif]

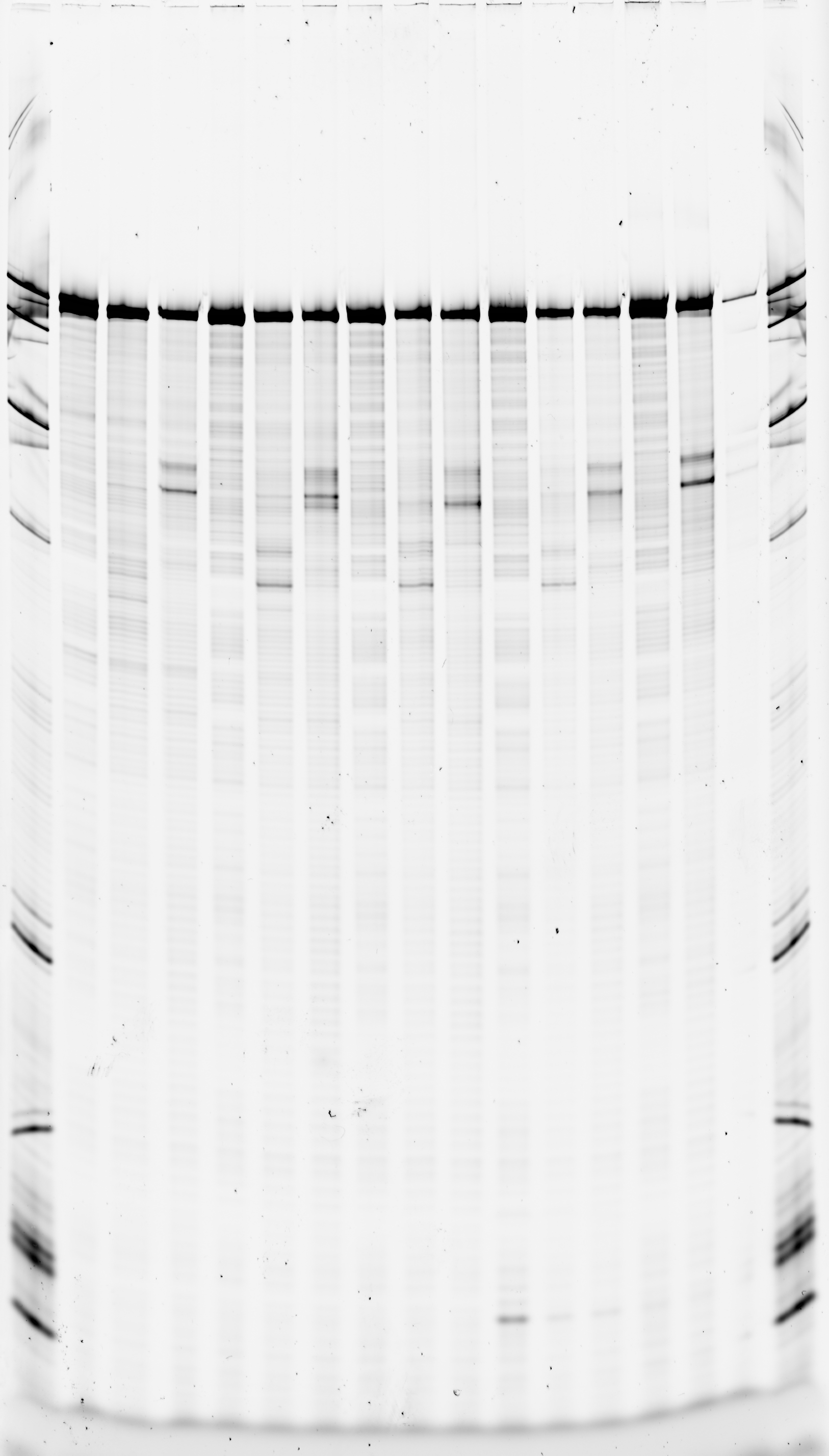

Supplement: Figure 1—source data 1. [file elife-52513-fig1-data1.zip › Figure1-sourcedata-original/NucMAPscans/23Oct2018xNucMAPgap-24C-14C-4C+5C+15Ccy3.tif]

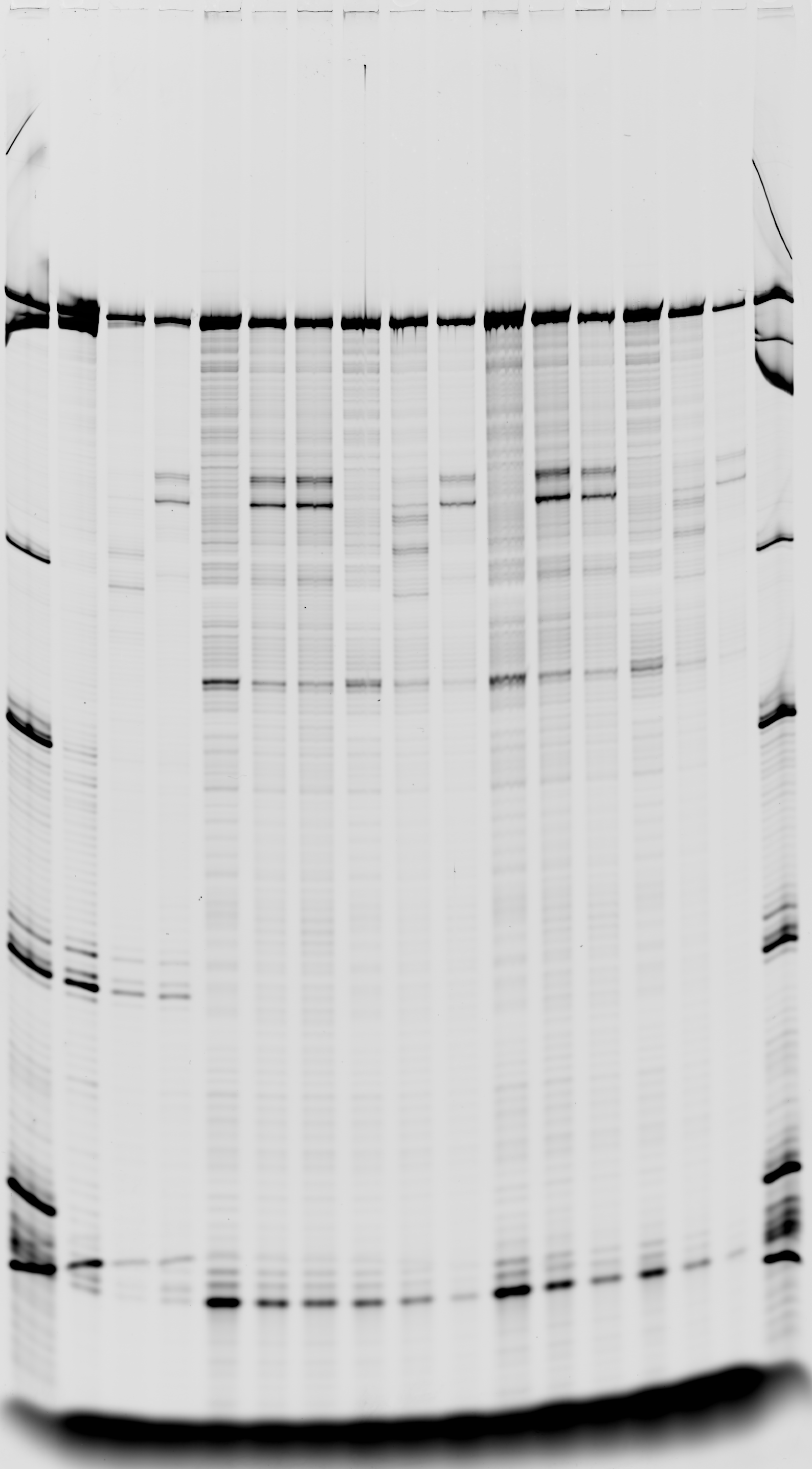

Supplement: Figure 1—source data 1. [file elife-52513-fig1-data1.zip › Figure1-sourcedata-original/NucMAPscans/24Oct2018xNucMAPgap0W47+25C+35CrepsCy5.tif]

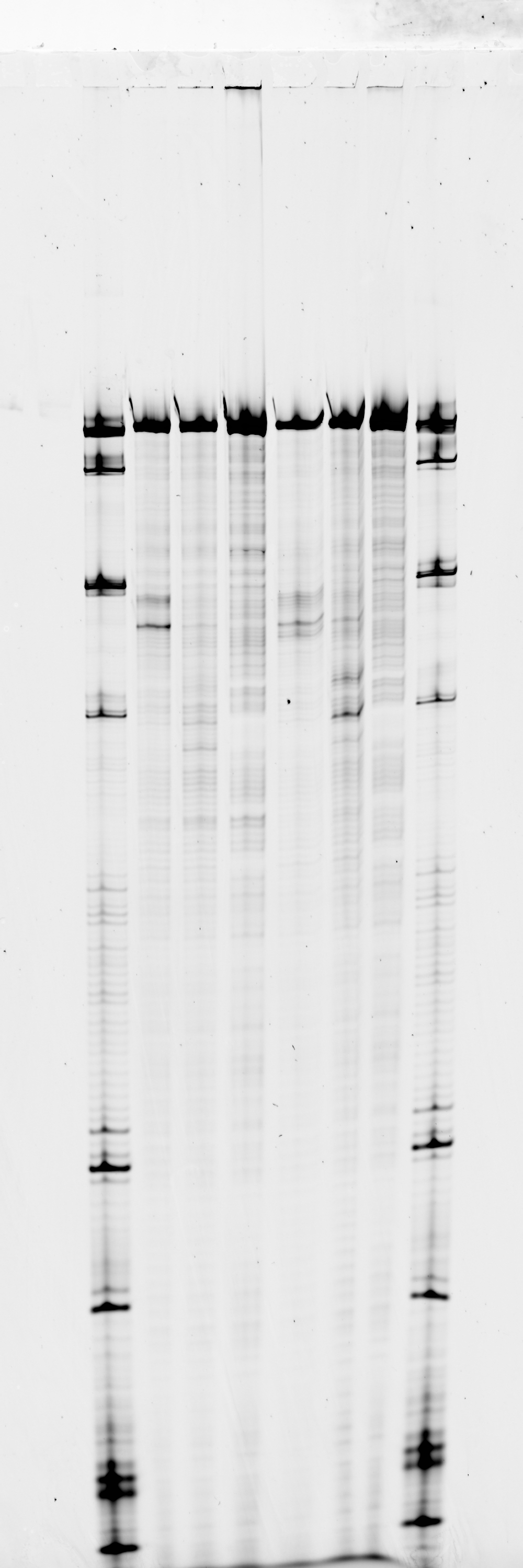

Supplement: Figure 1—source data 1. [file elife-52513-fig1-data1.zip › Figure1-sourcedata-original/NucMAPscans/26Oct2018xNucMAPgap-24C-14C-Cy3.tif]

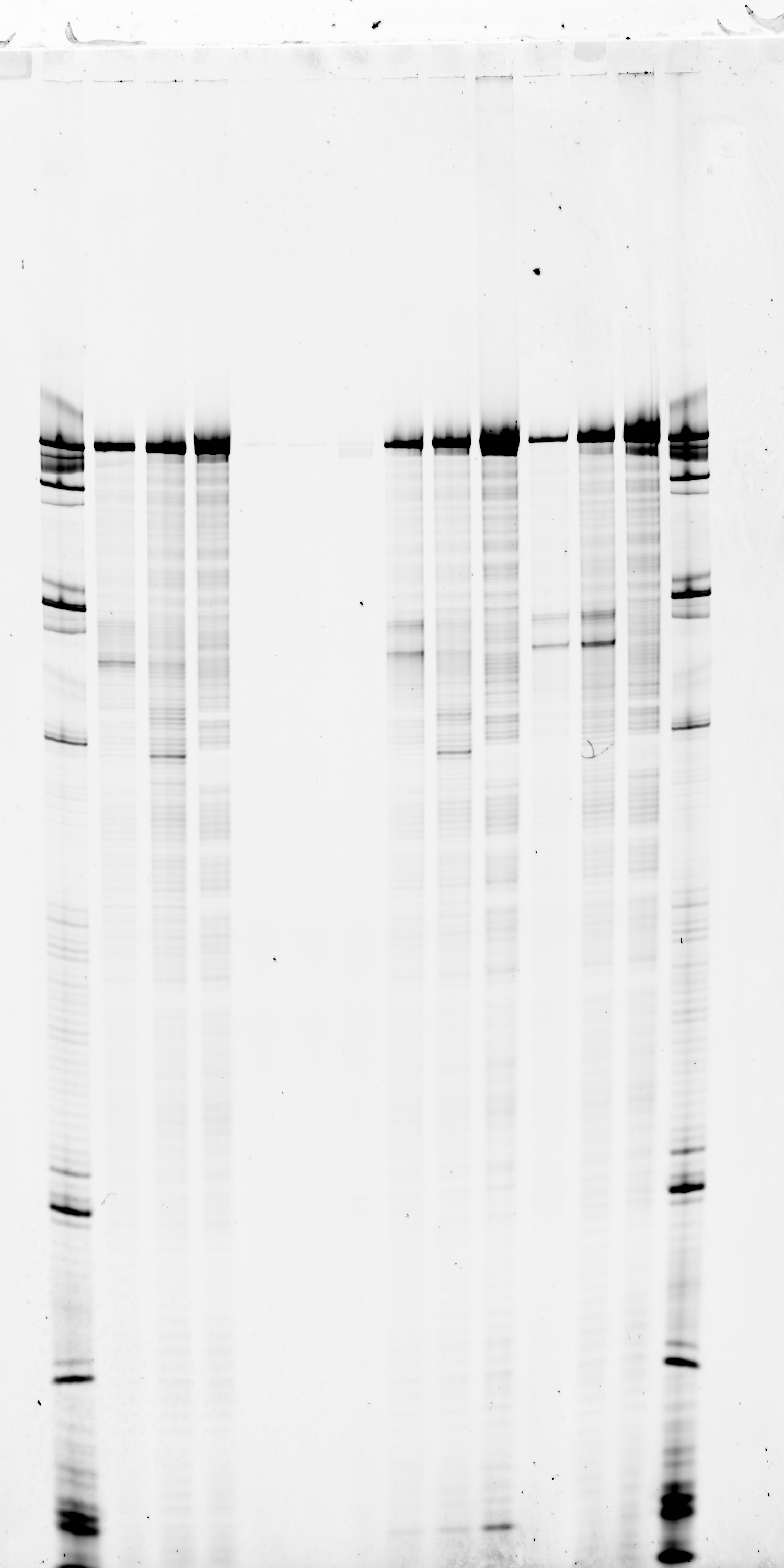

Supplement: Figure 1—source data 1. [file elife-52513-fig1-data1.zip › Figure1-sourcedata-original/NucMAPscans/26Oct2018xNucMAPgap-4C0W47+5C+15C-Cy3.tif]

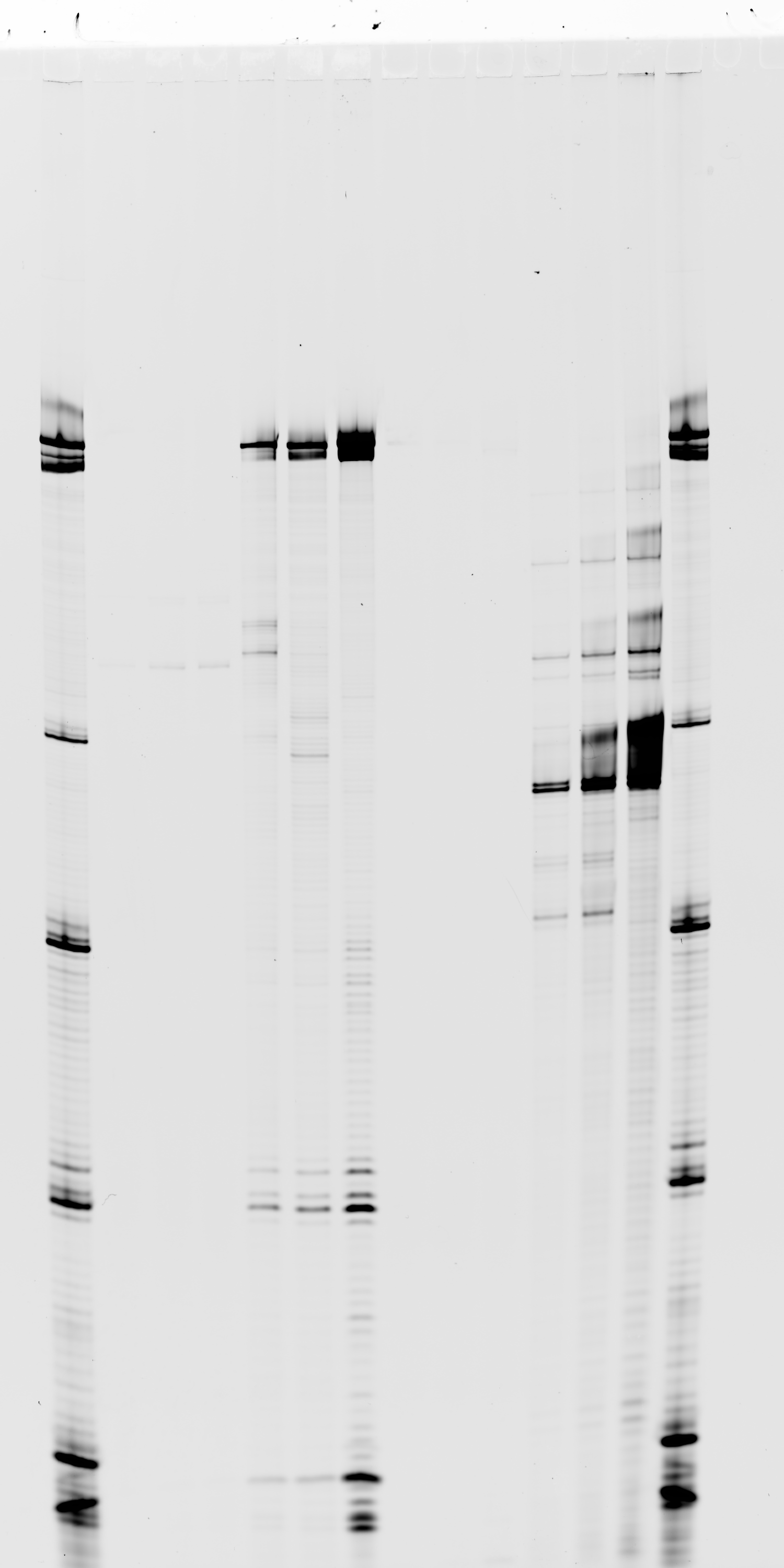

Supplement: Figure 1—source data 1. [file elife-52513-fig1-data1.zip › Figure1-sourcedata-original/NucMAPscans/26Oct2018xNucMAPgap-4C0W47+5C+15C-Cy5.tif]

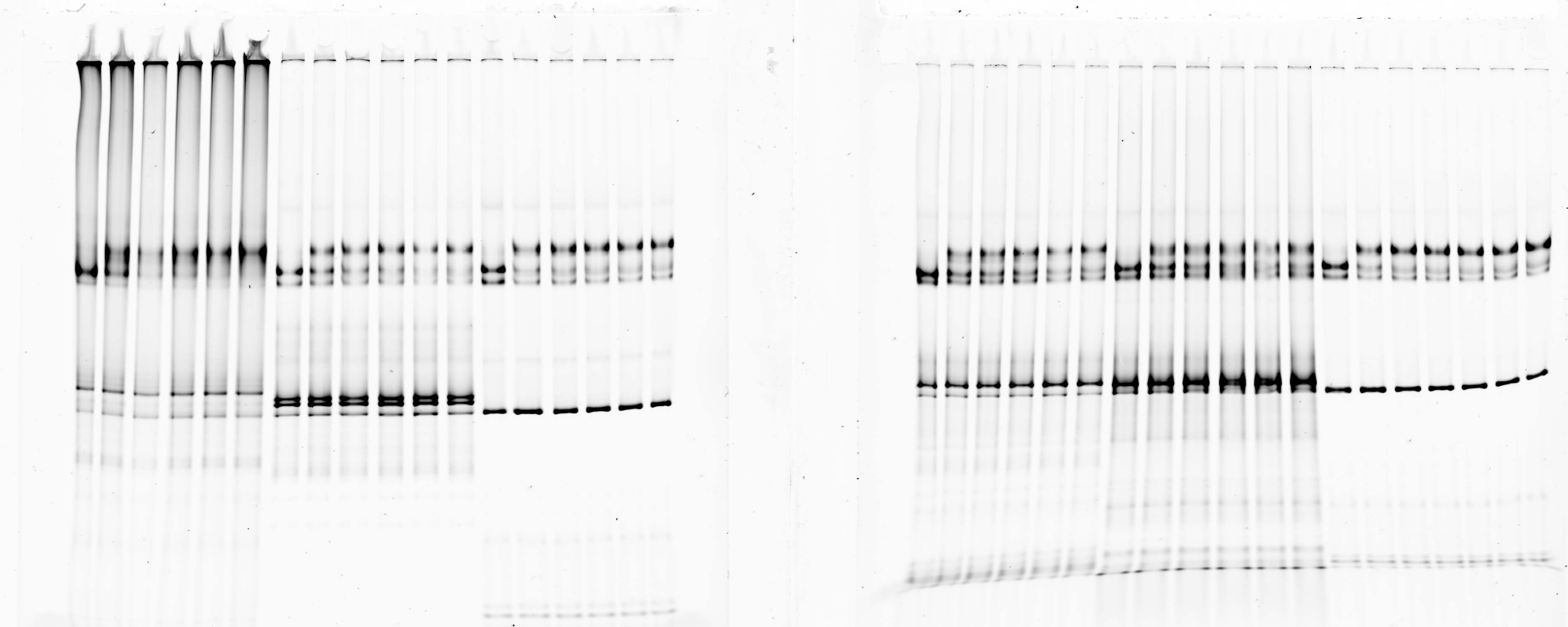

Supplement: Figure 1—source data 1. [file elife-52513-fig1-data1.zip › Figure1-sourcedata-original/Slidingscans/13April2018-4C+5C+35C_+74C+85C0W47Cy3dual.tif]

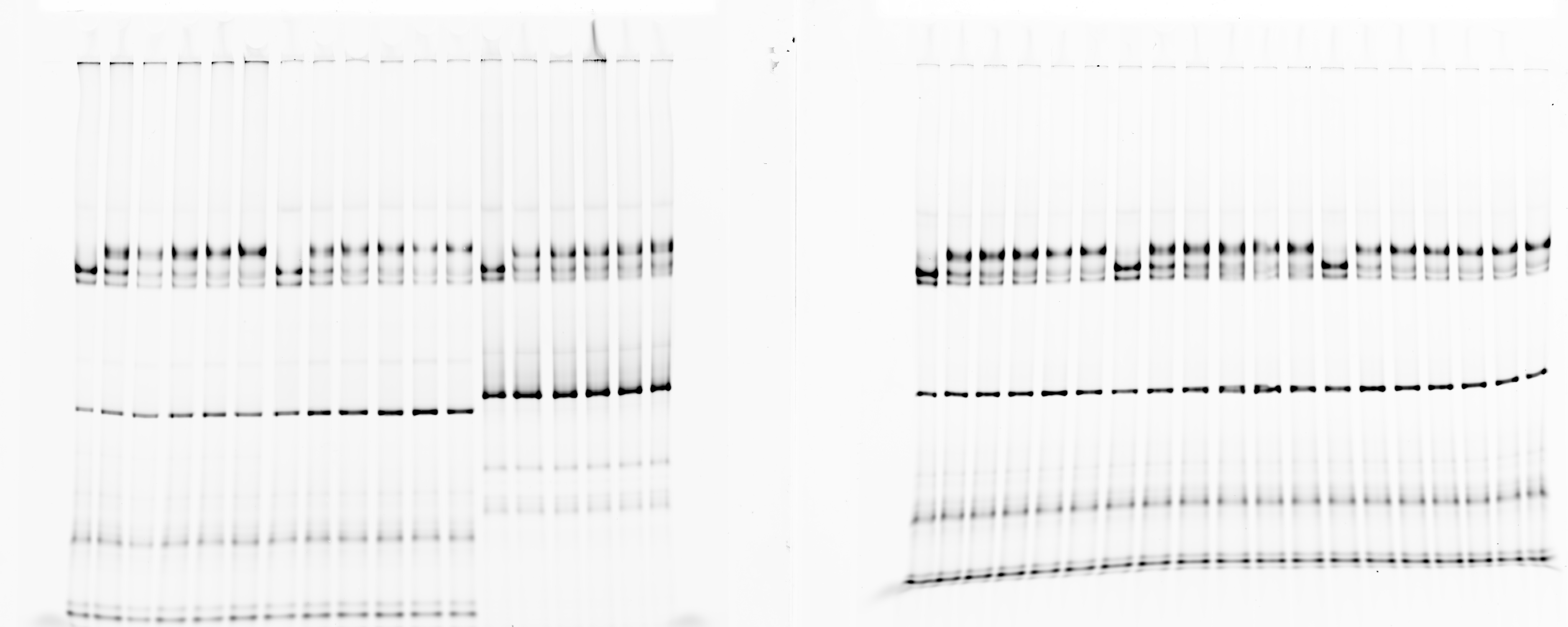

Supplement: Figure 1—source data 1. [file elife-52513-fig1-data1.zip › Figure1-sourcedata-original/Slidingscans/13April2018slide0W47Cy5dual.tif]

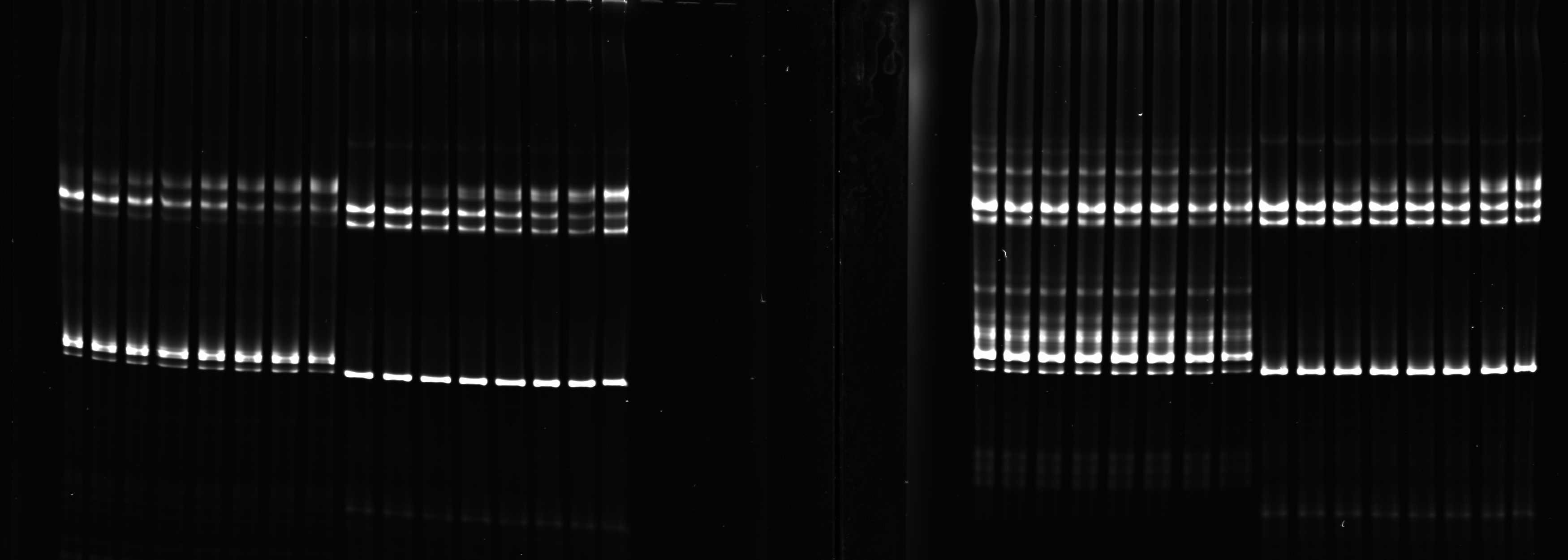

Supplement: Figure 1—source data 1. [file elife-52513-fig1-data1.zip › Figure1-sourcedata-original/Slidingscans/13Oct2017slide-24G0W47_+15G0W47dualCy3.tif]

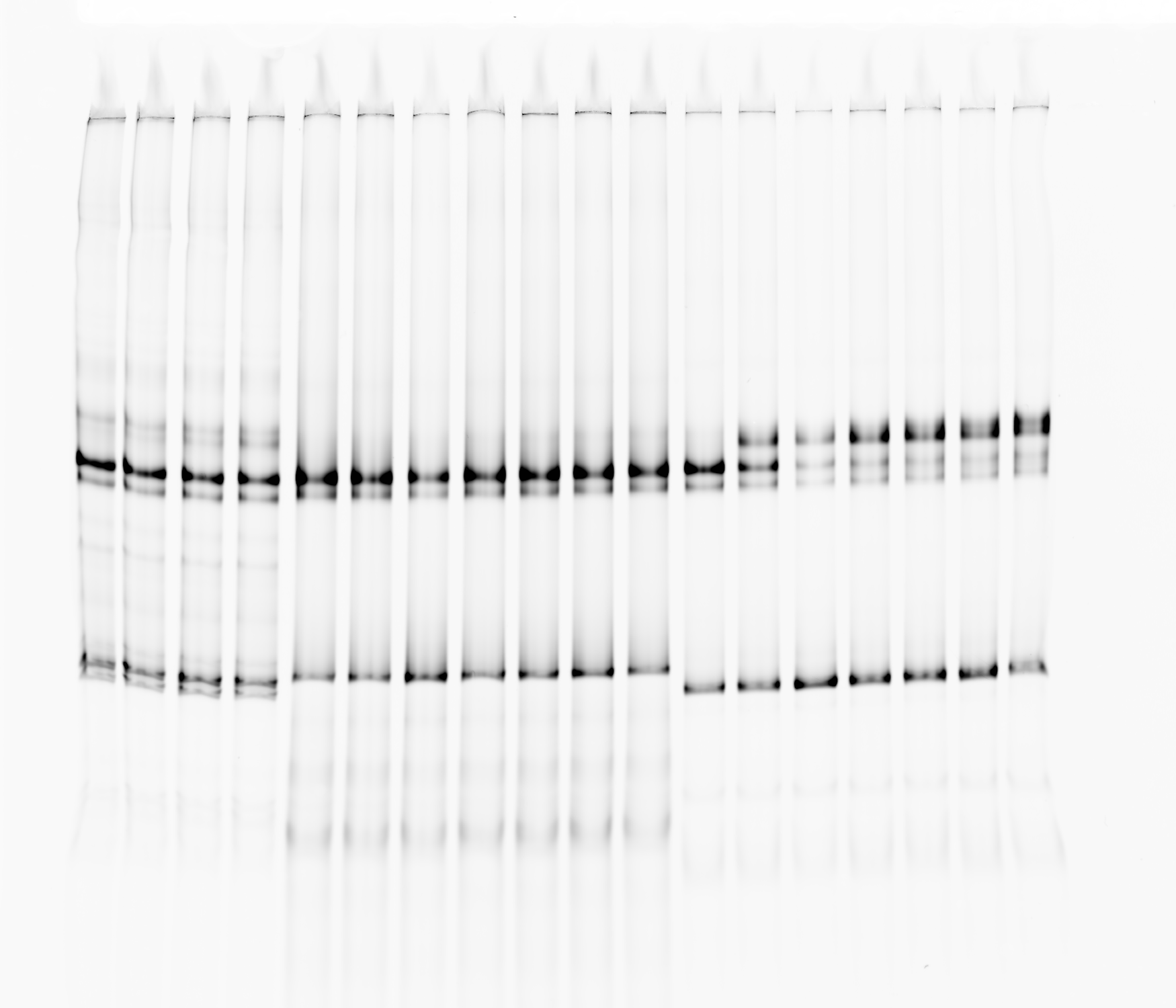

Supplement: Figure 1—source data 1. [file elife-52513-fig1-data1.zip › Figure1-sourcedata-original/Slidingscans/18Oct2018slide+15C+25C+35Ccy5.tif]

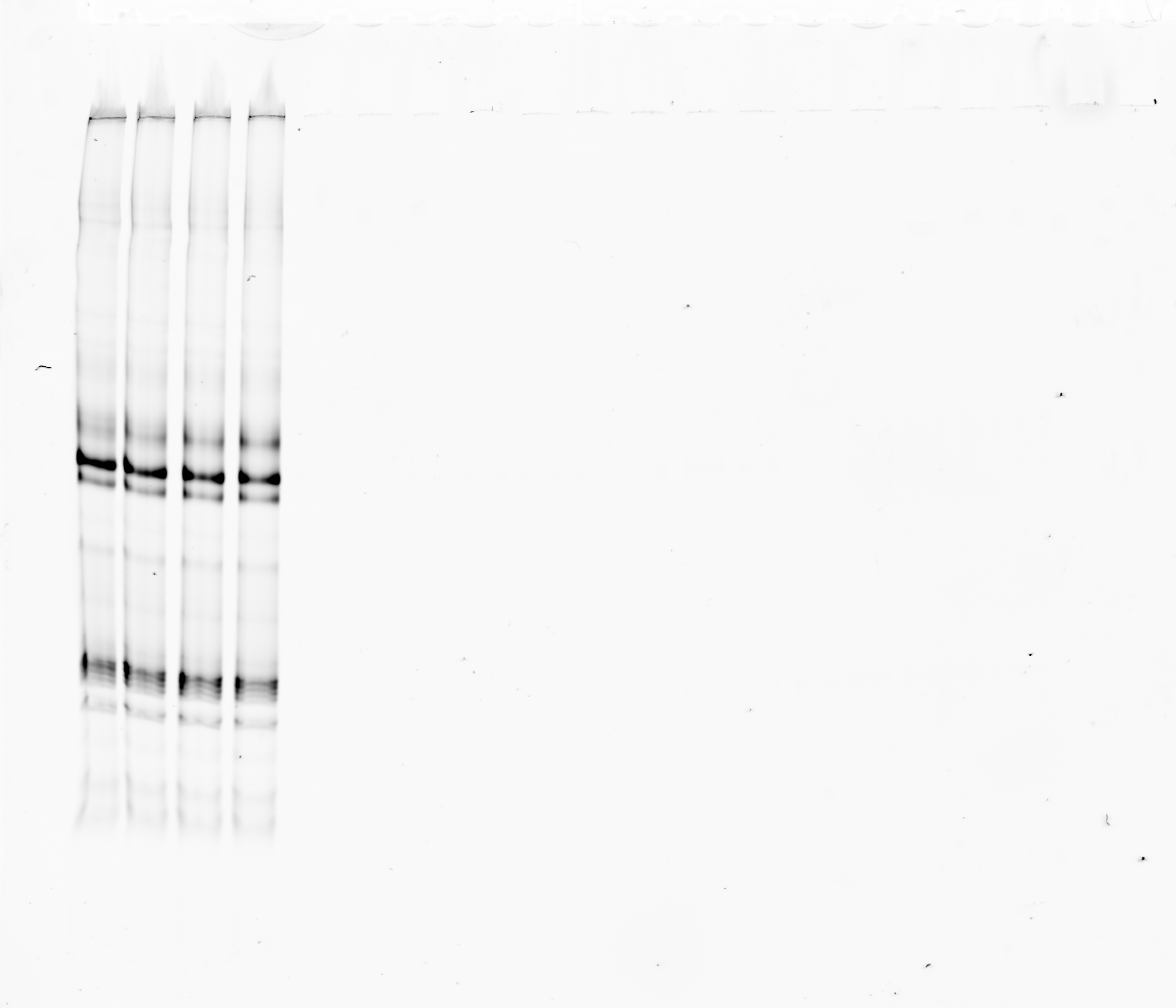

Supplement: Figure 1—source data 1. [file elife-52513-fig1-data1.zip › Figure1-sourcedata-original/Slidingscans/18Oct2018slide+15Ccy3.tif]

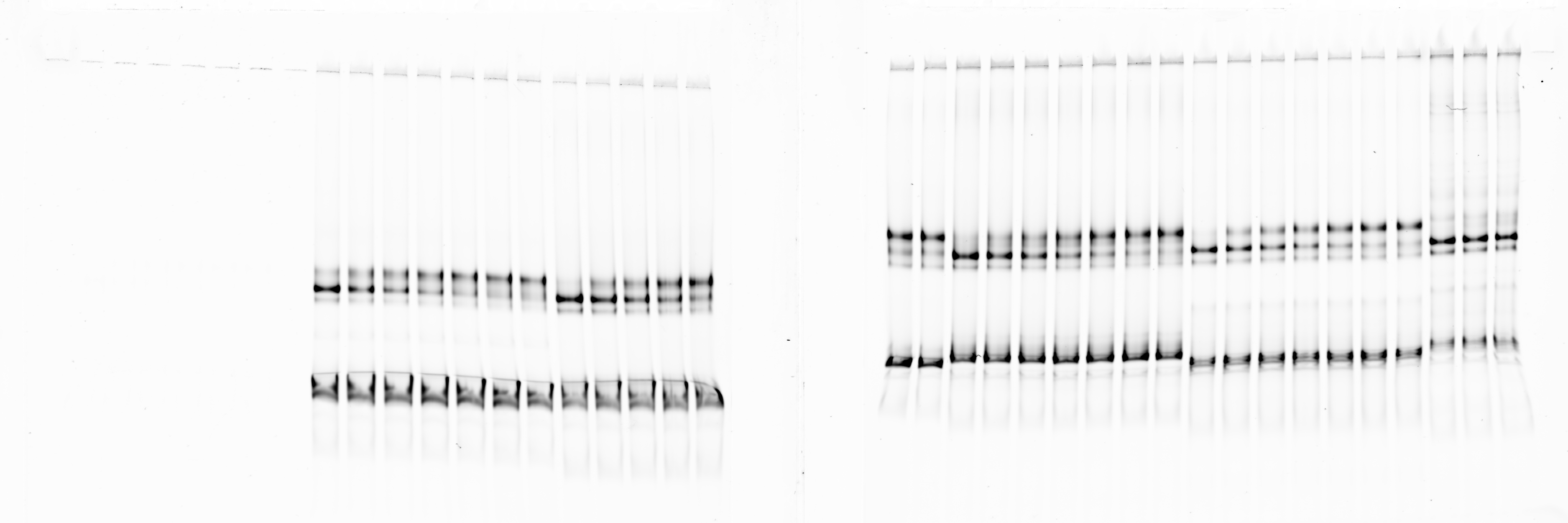

Supplement: Figure 1—source data 1. [file elife-52513-fig1-data1.zip › Figure1-sourcedata-original/Slidingscans/18Oct2018slide-24C-14C_-4C+5C+15Ccy3.tif]

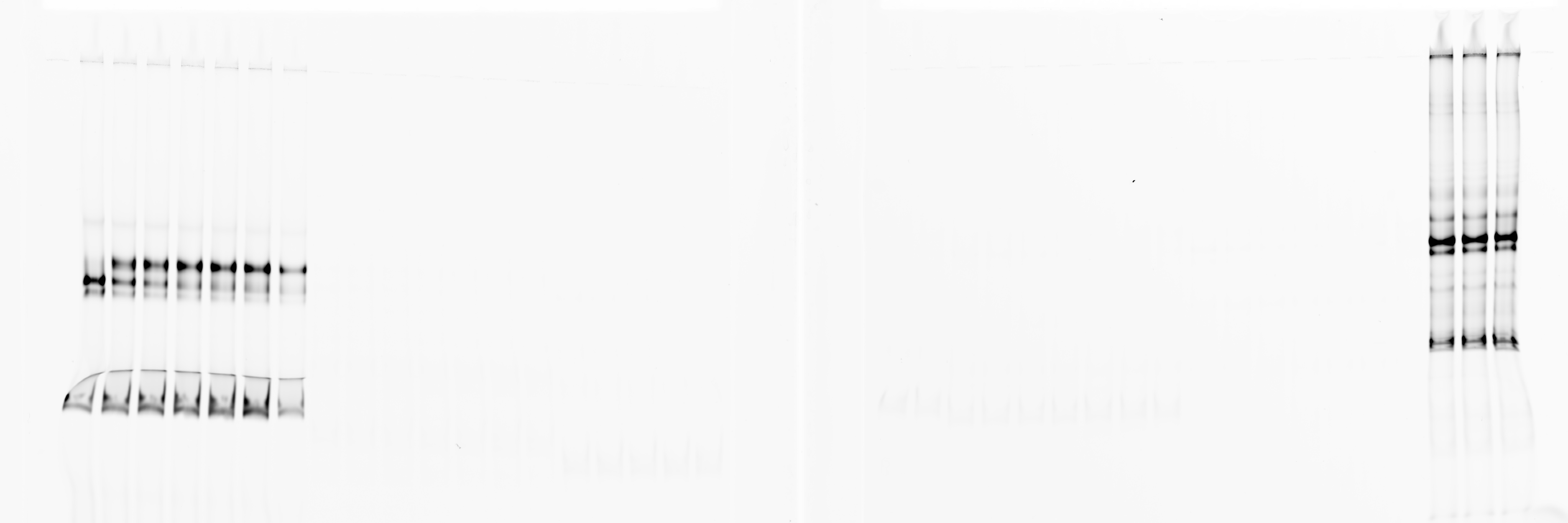

Supplement: Figure 1—source data 1. [file elife-52513-fig1-data1.zip › Figure1-sourcedata-original/Slidingscans/18Oct2018slide0W47_+15Ccy5.tif]

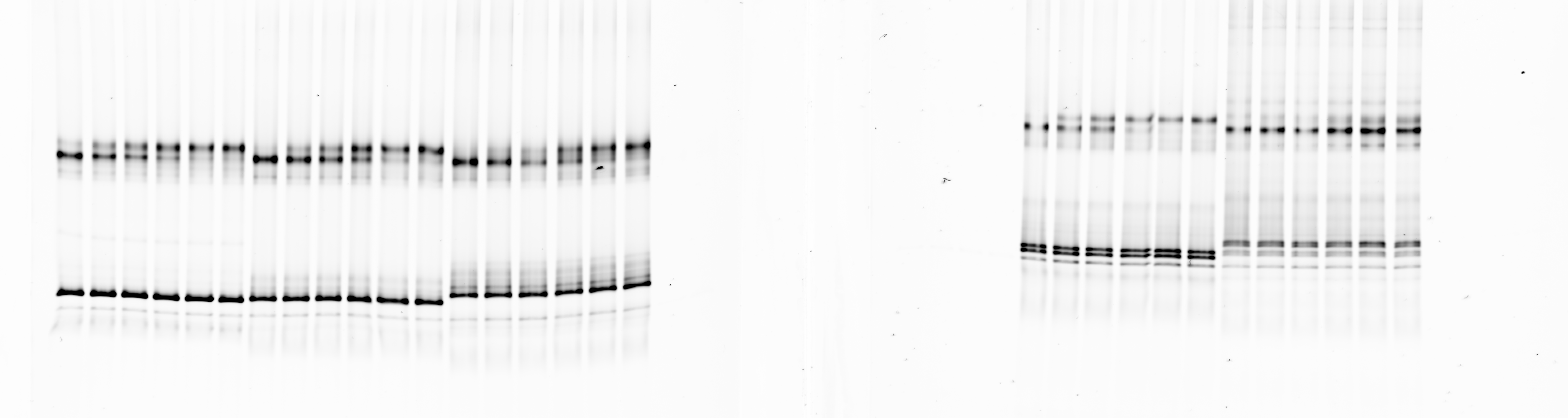

Supplement: Figure 1—source data 1. [file elife-52513-fig1-data1.zip › Figure1-sourcedata-original/Slidingscans/23Oct2018gap-24C-14C-4C_+5C+15Ccy3.tif]

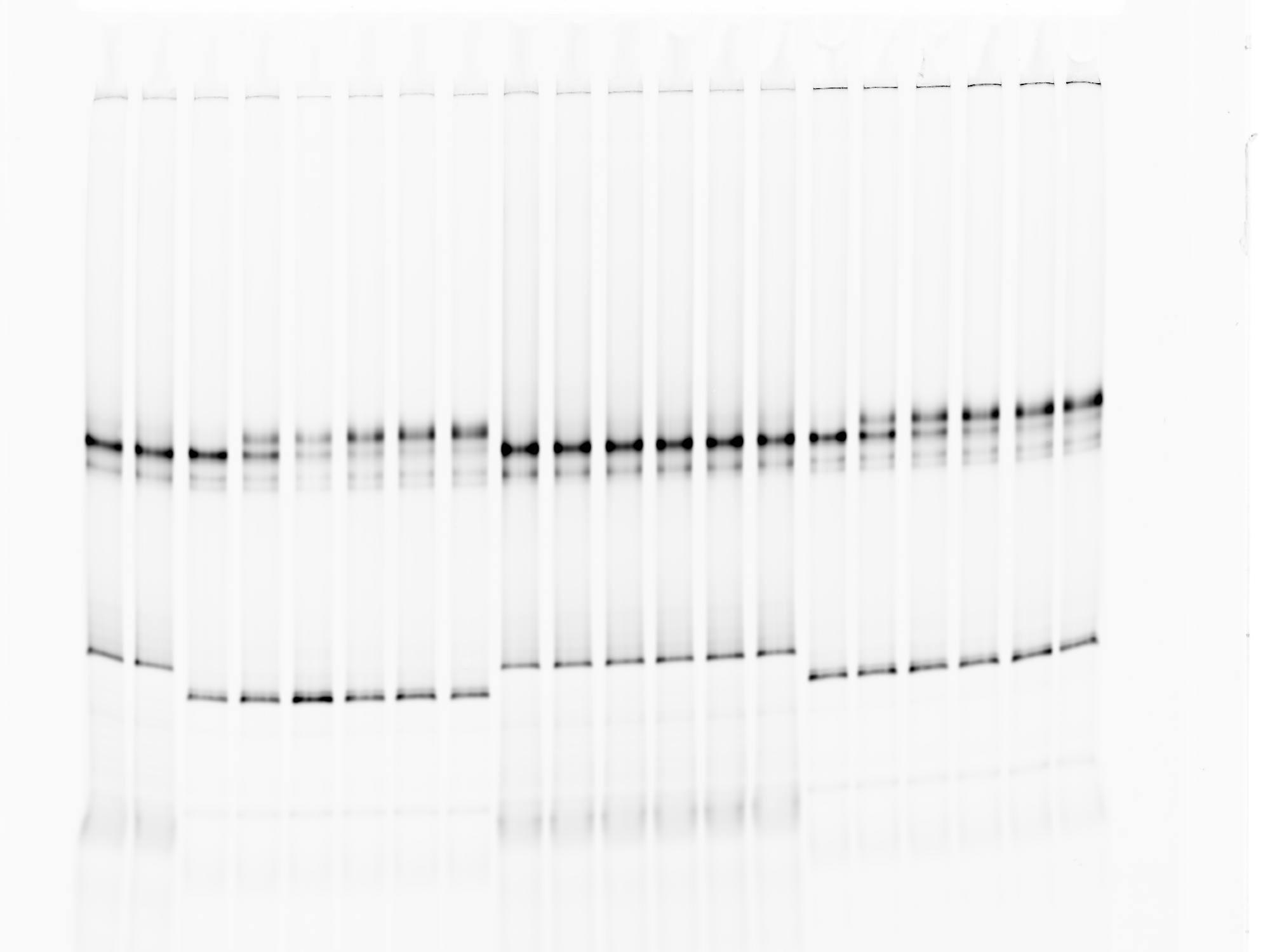

Supplement: Figure 1—source data 1. [file elife-52513-fig1-data1.zip › Figure1-sourcedata-original/Slidingscans/23Oct2018slide+25C+35CrepsCy5.tif]

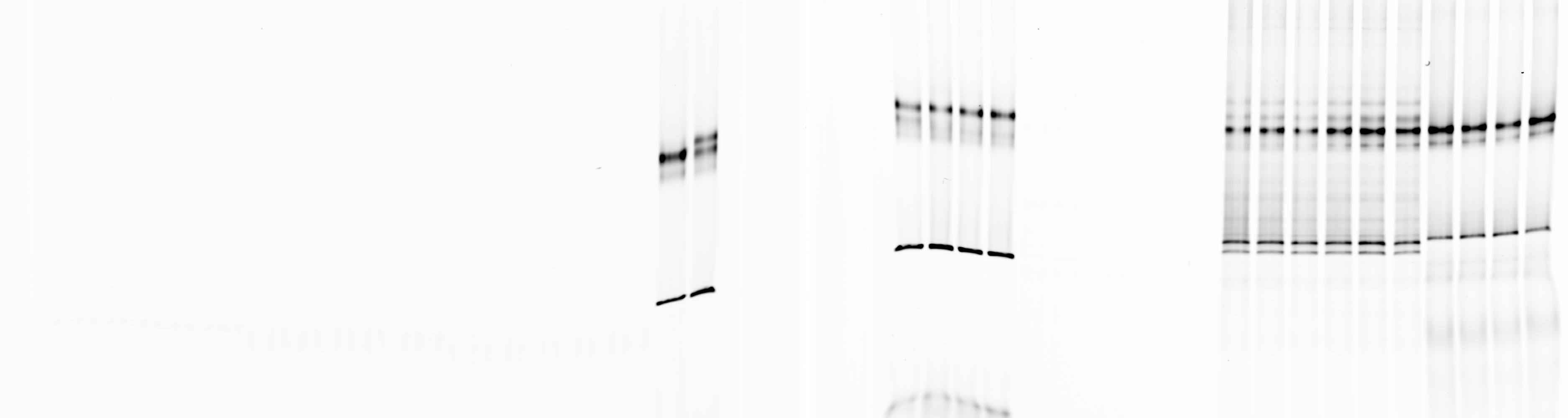

Supplement: Figure 1—source data 1. [file elife-52513-fig1-data1.zip › Figure1-sourcedata-original/Slidingscans/23OCt2018slide0W47_+15C+25Ccy5.tif]

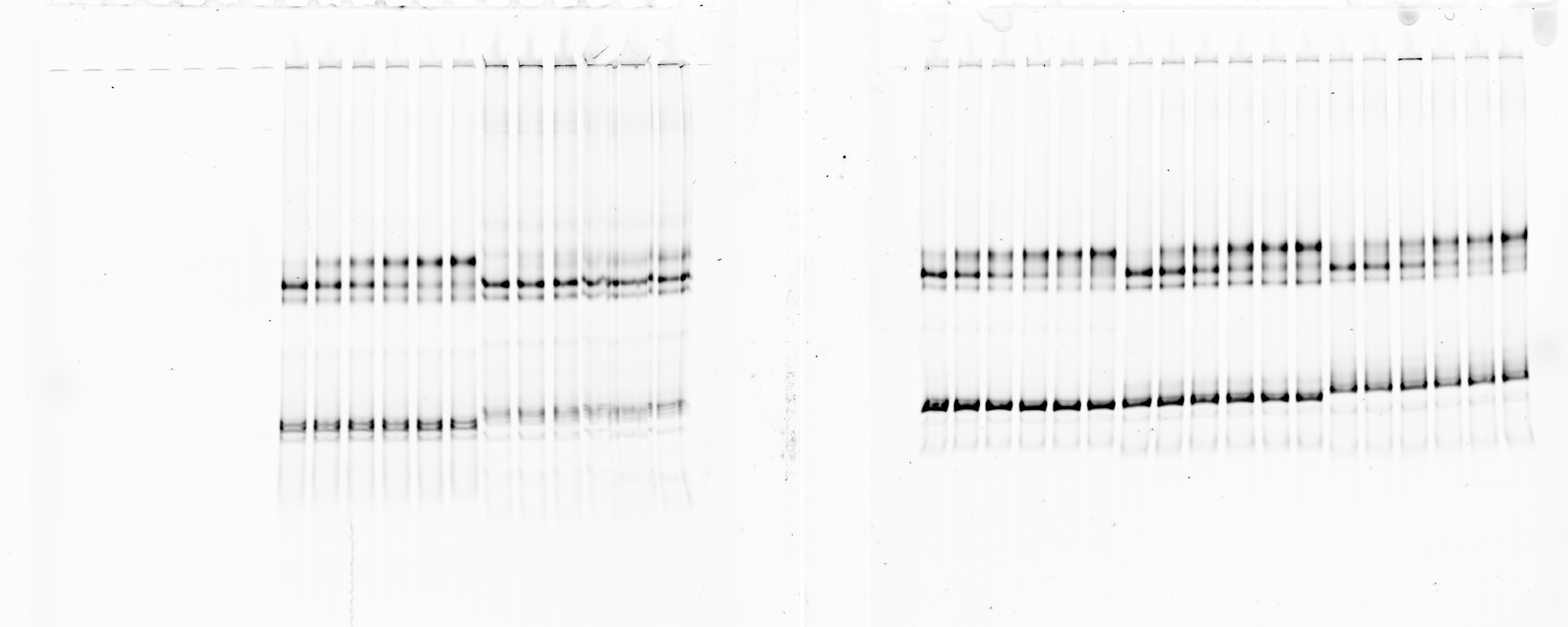

Supplement: Figure 1—source data 1. [file elife-52513-fig1-data1.zip › Figure1-sourcedata-original/Slidingscans/25Oct2018slide+5C+15C_-24C-14C-4Ccy3.tif]

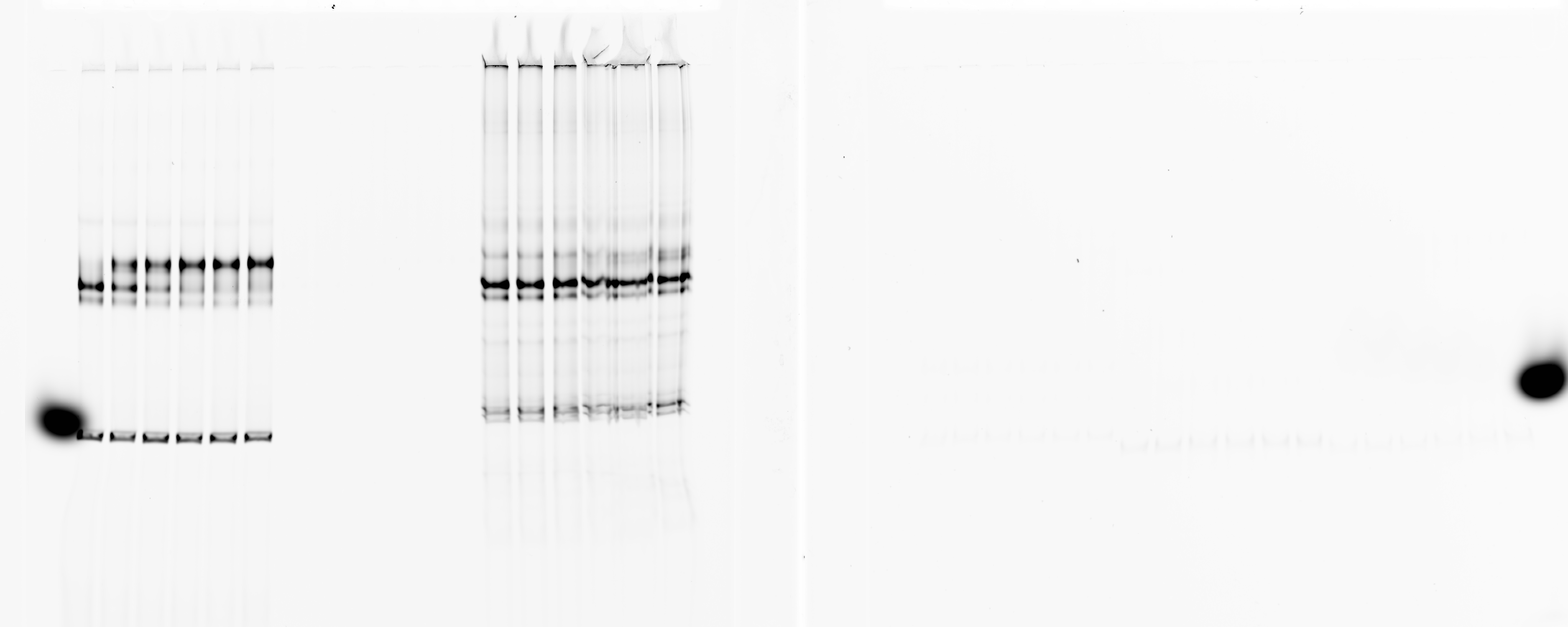

Supplement: Figure 1—source data 1. [file elife-52513-fig1-data1.zip › Figure1-sourcedata-original/Slidingscans/25Oct2018slide0W47-15Ccy5.tif]

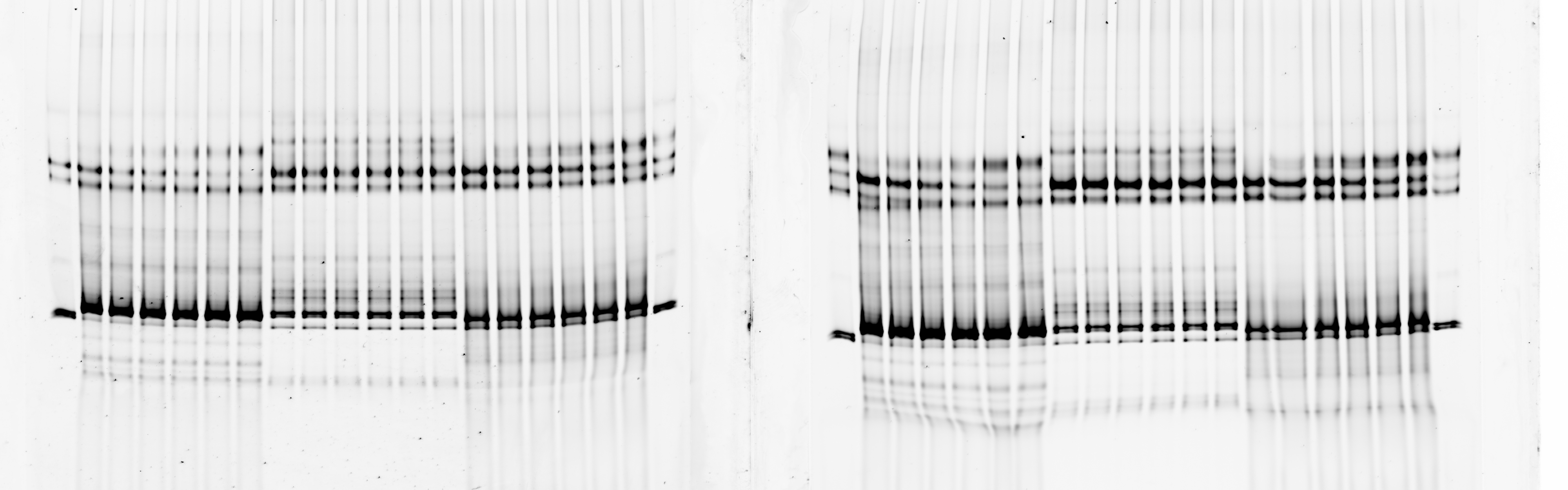

Supplement: Figure 1—source data 1. [file elife-52513-fig1-data1.zip › Figure1-sourcedata-original/Slidingscans/3Dec2018-24G+15G+85Cslide.tif]

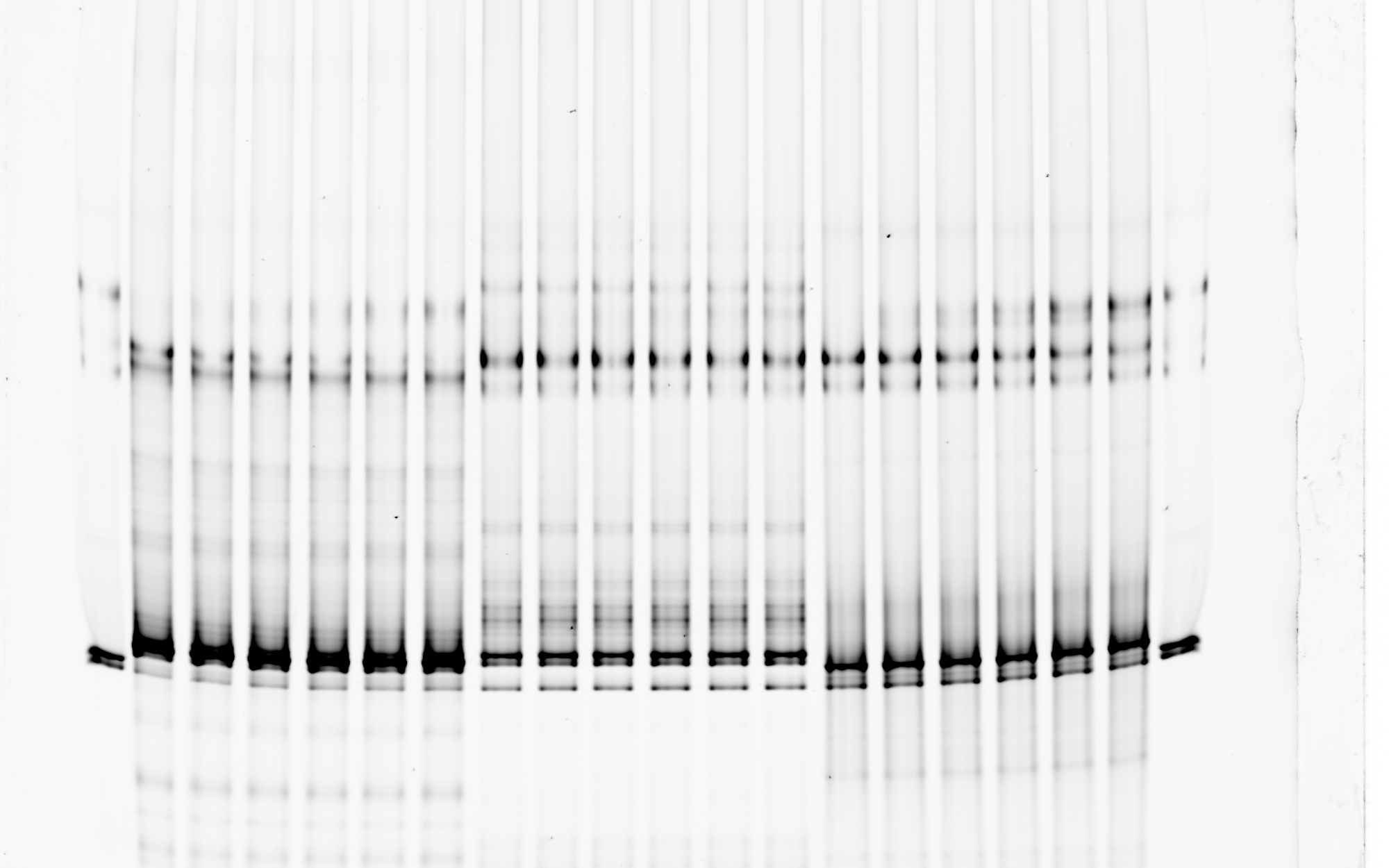

Supplement: Figure 1—source data 1. [file elife-52513-fig1-data1.zip › Figure1-sourcedata-original/Slidingscans/3Dec2018-24G+15G+85Cslide2.tif]

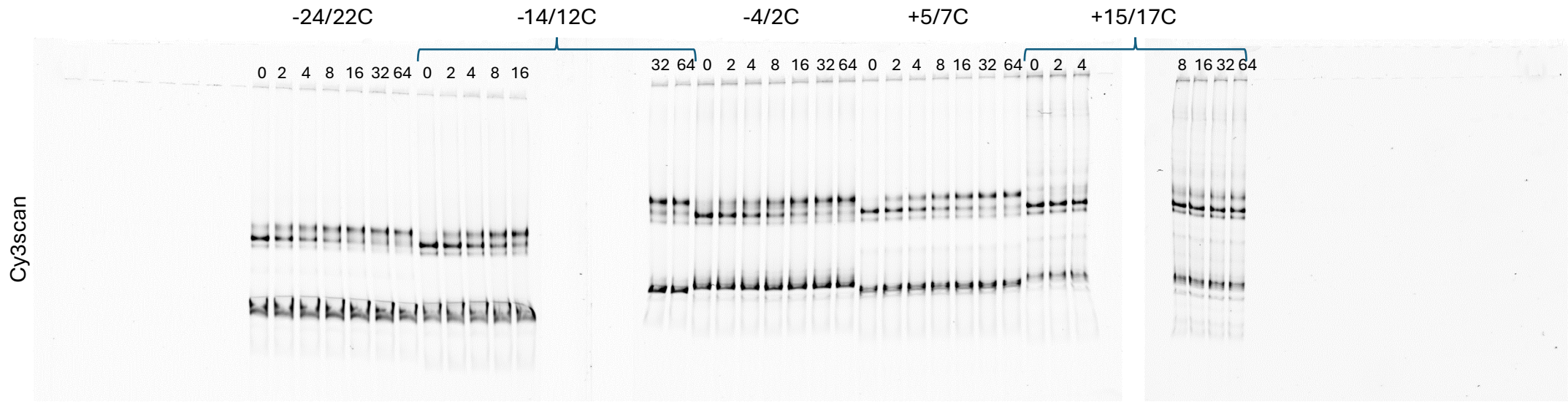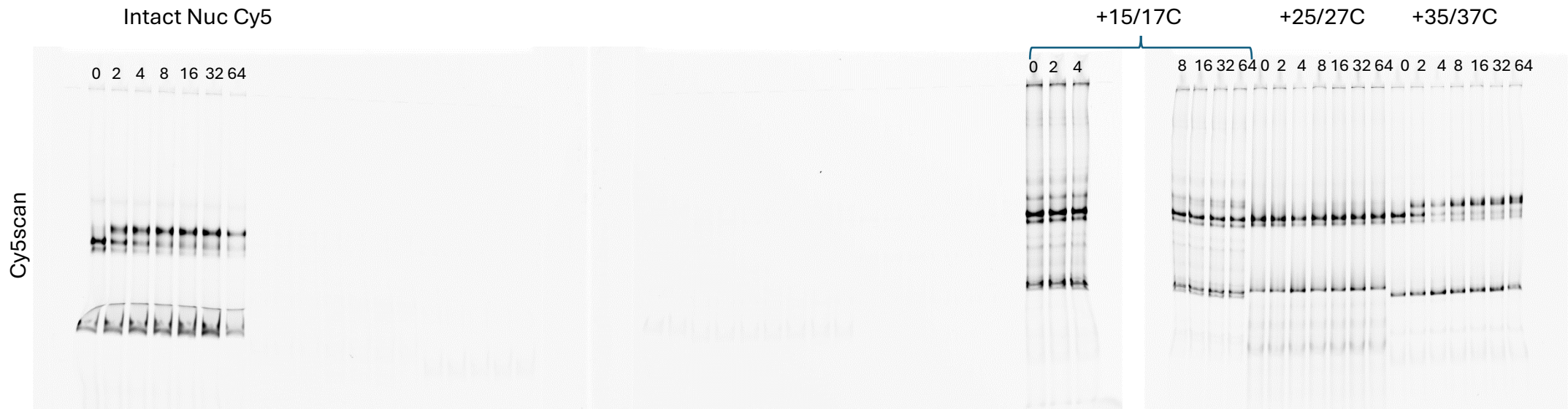

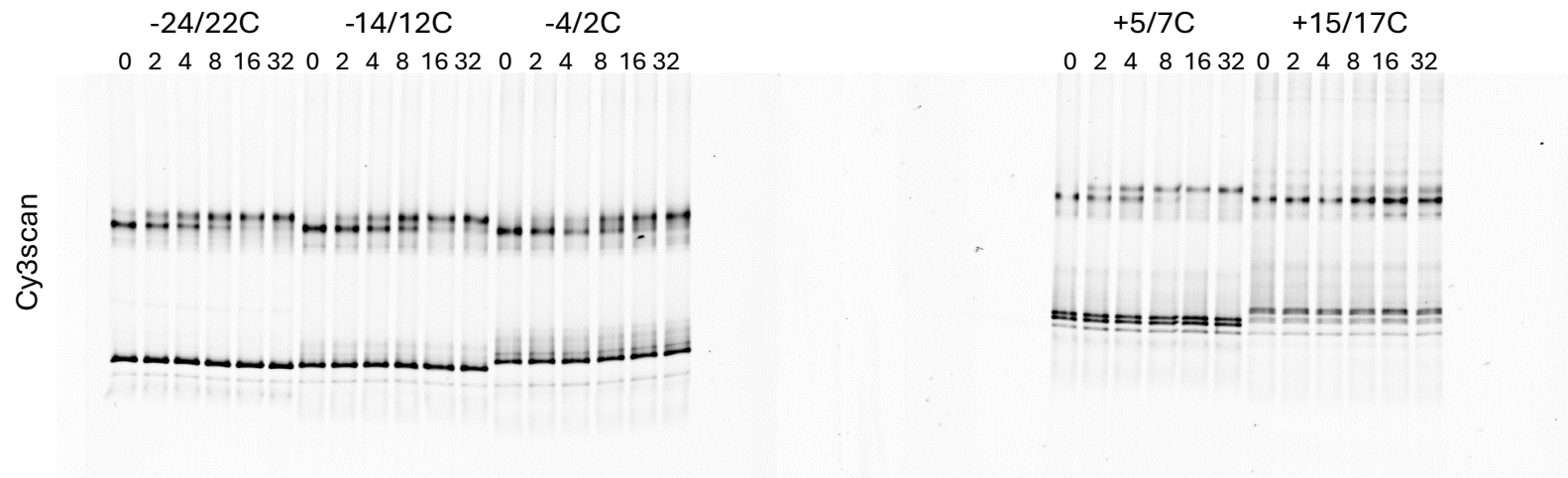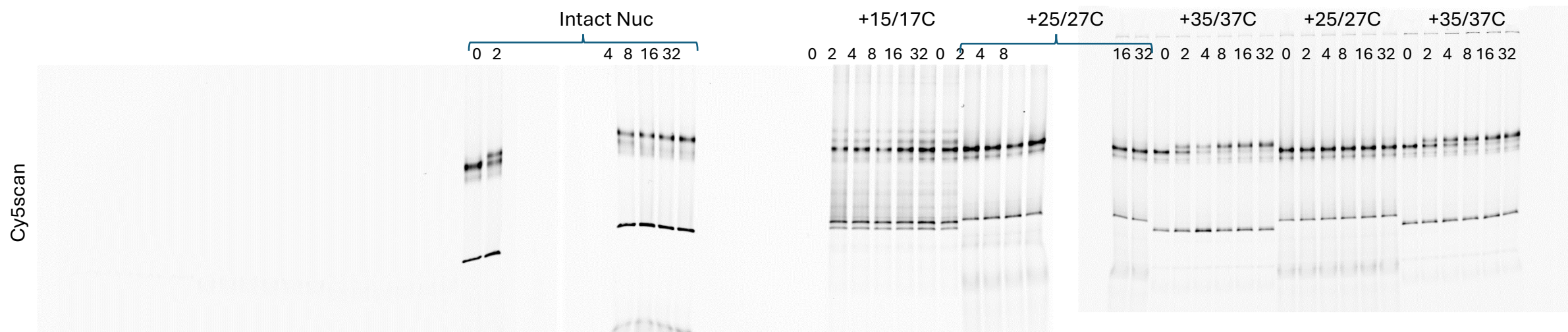

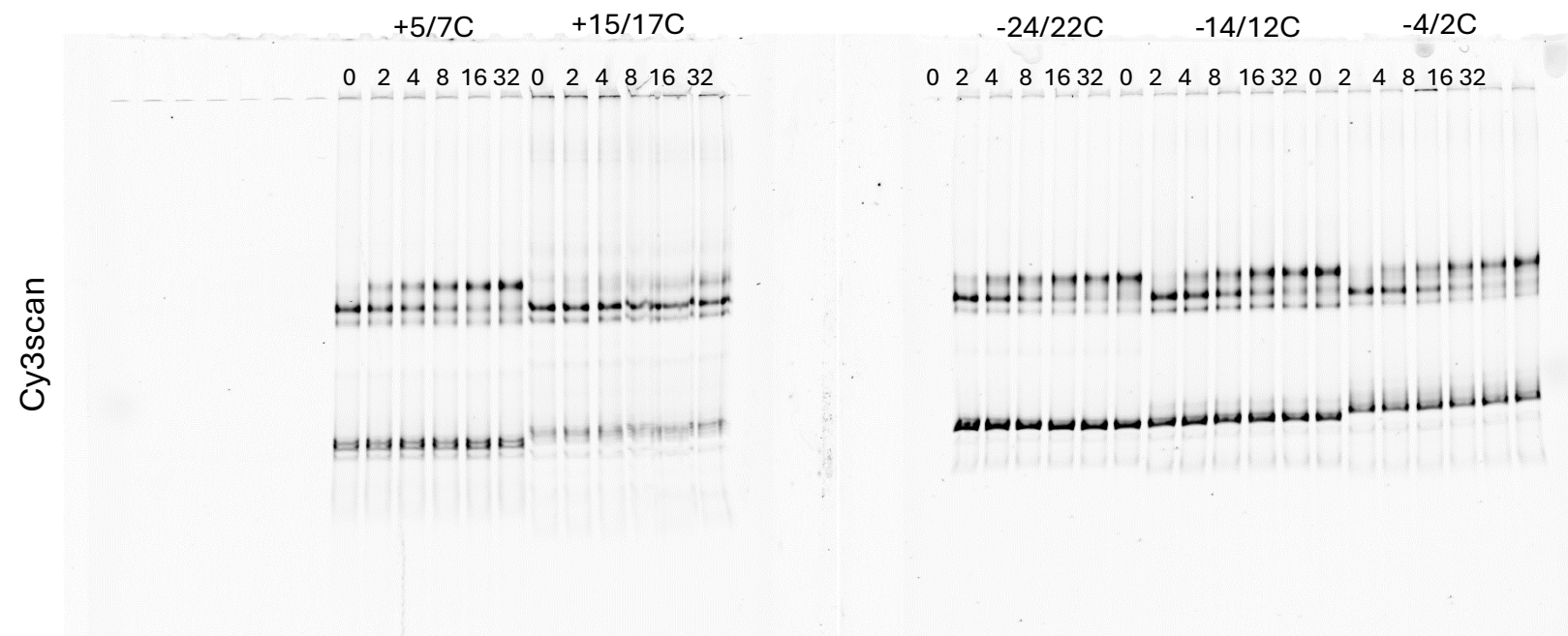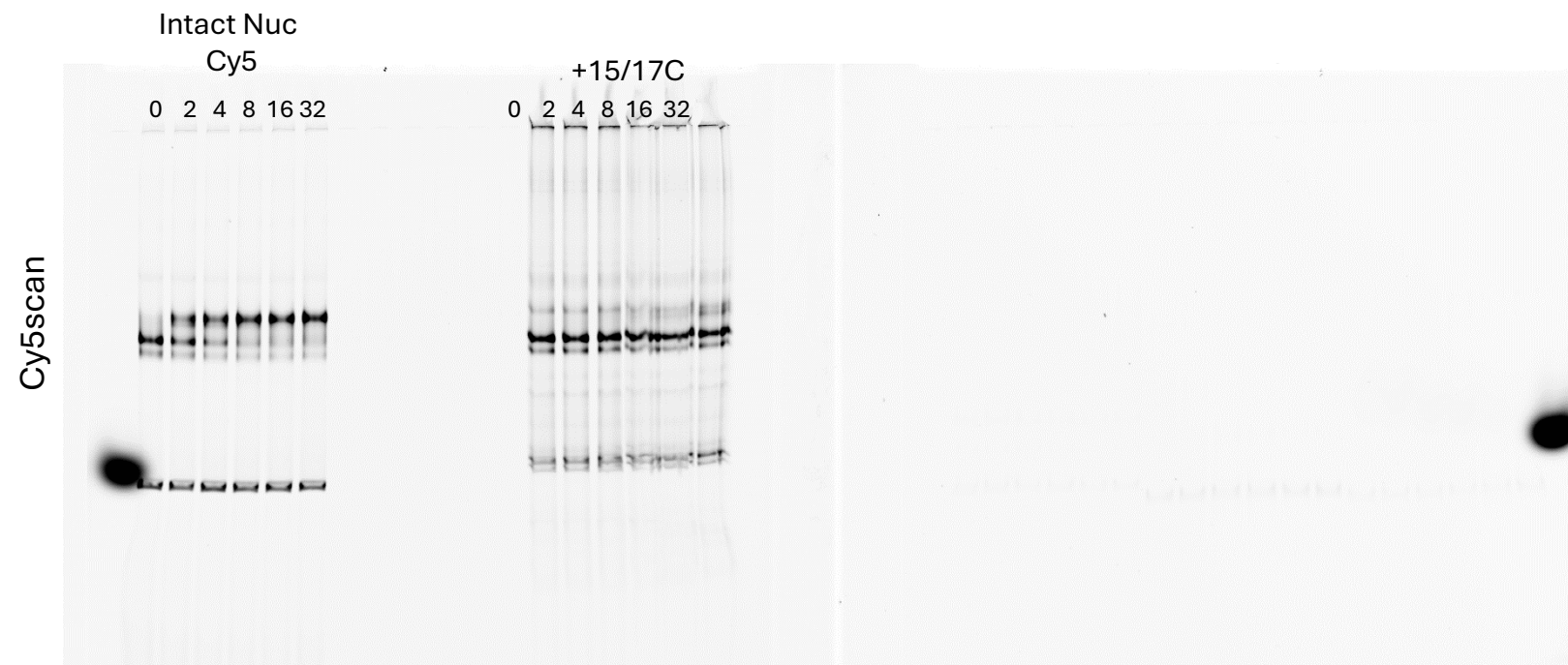

Cy3scan

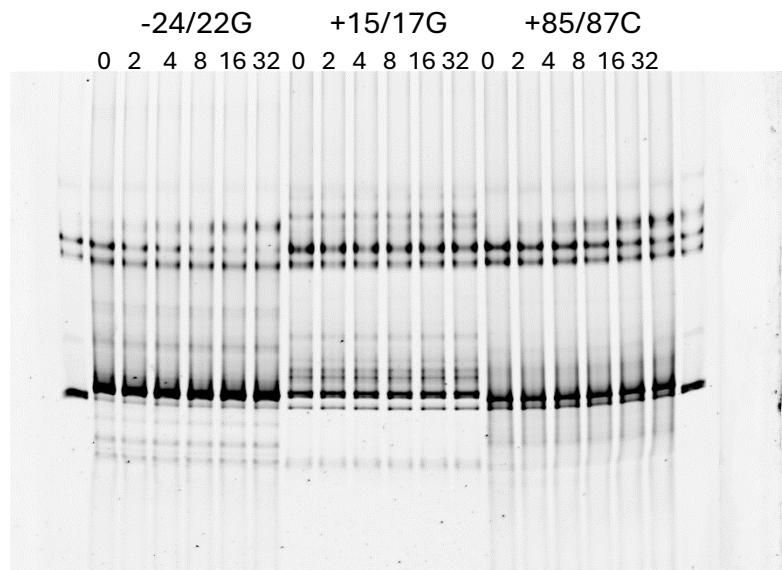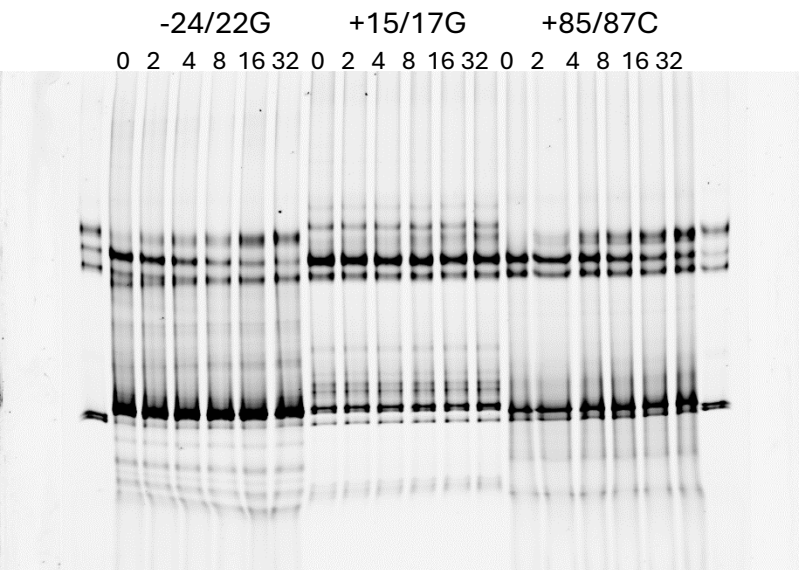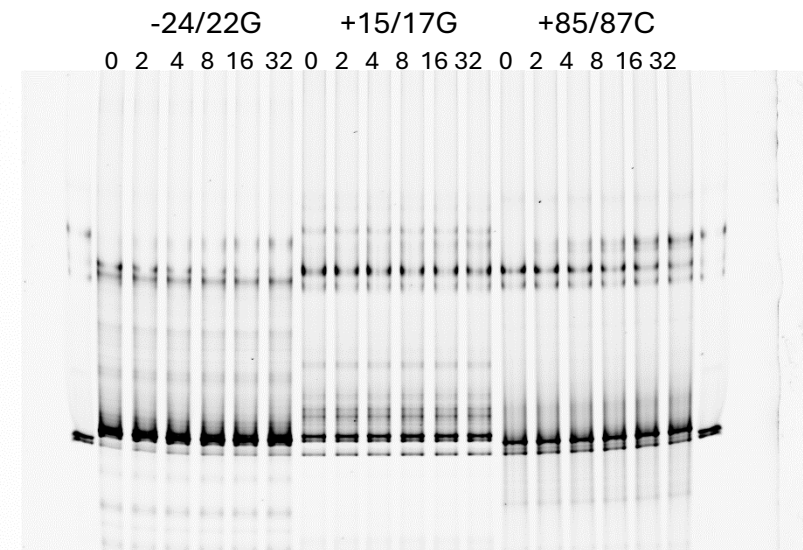

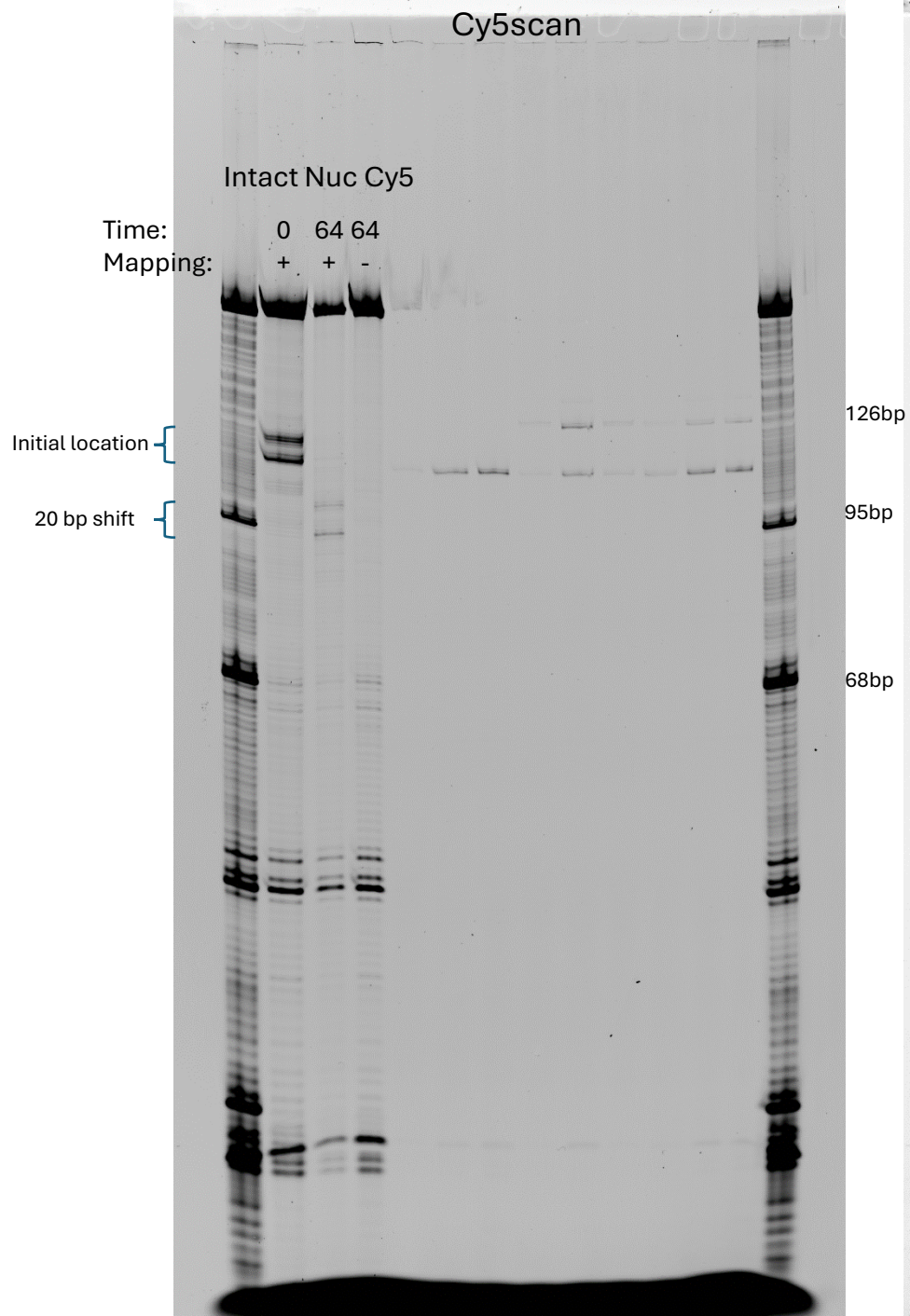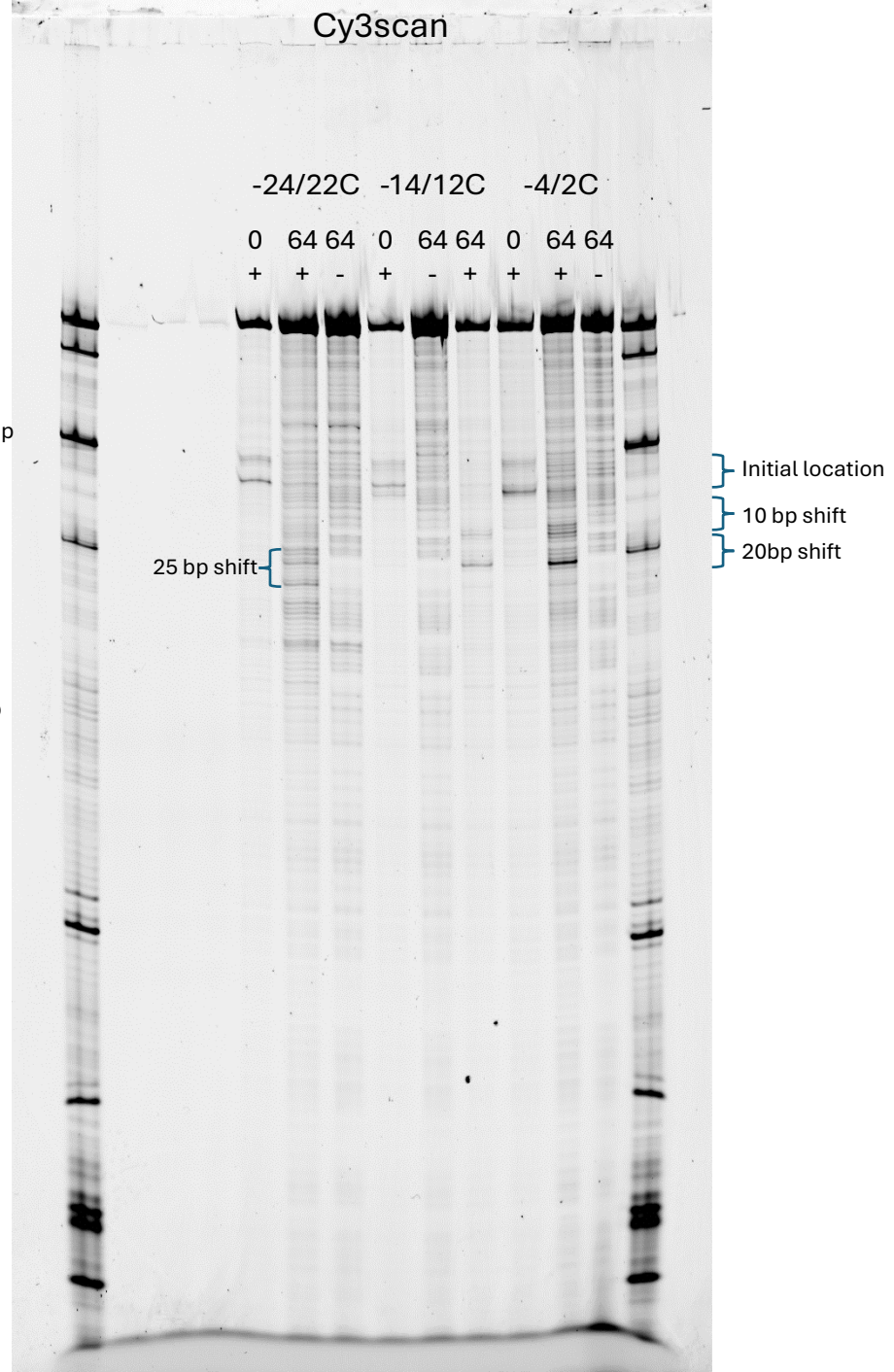

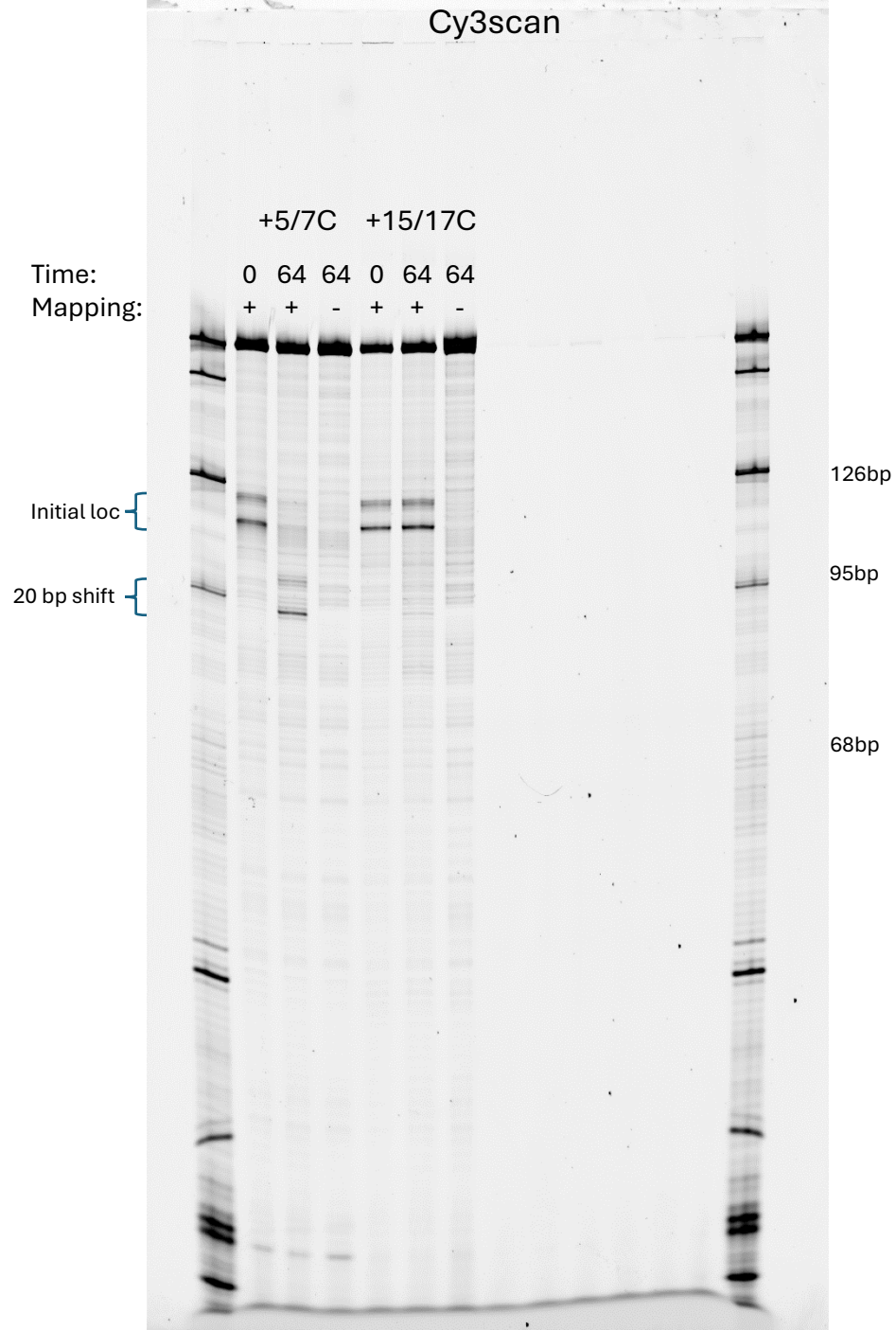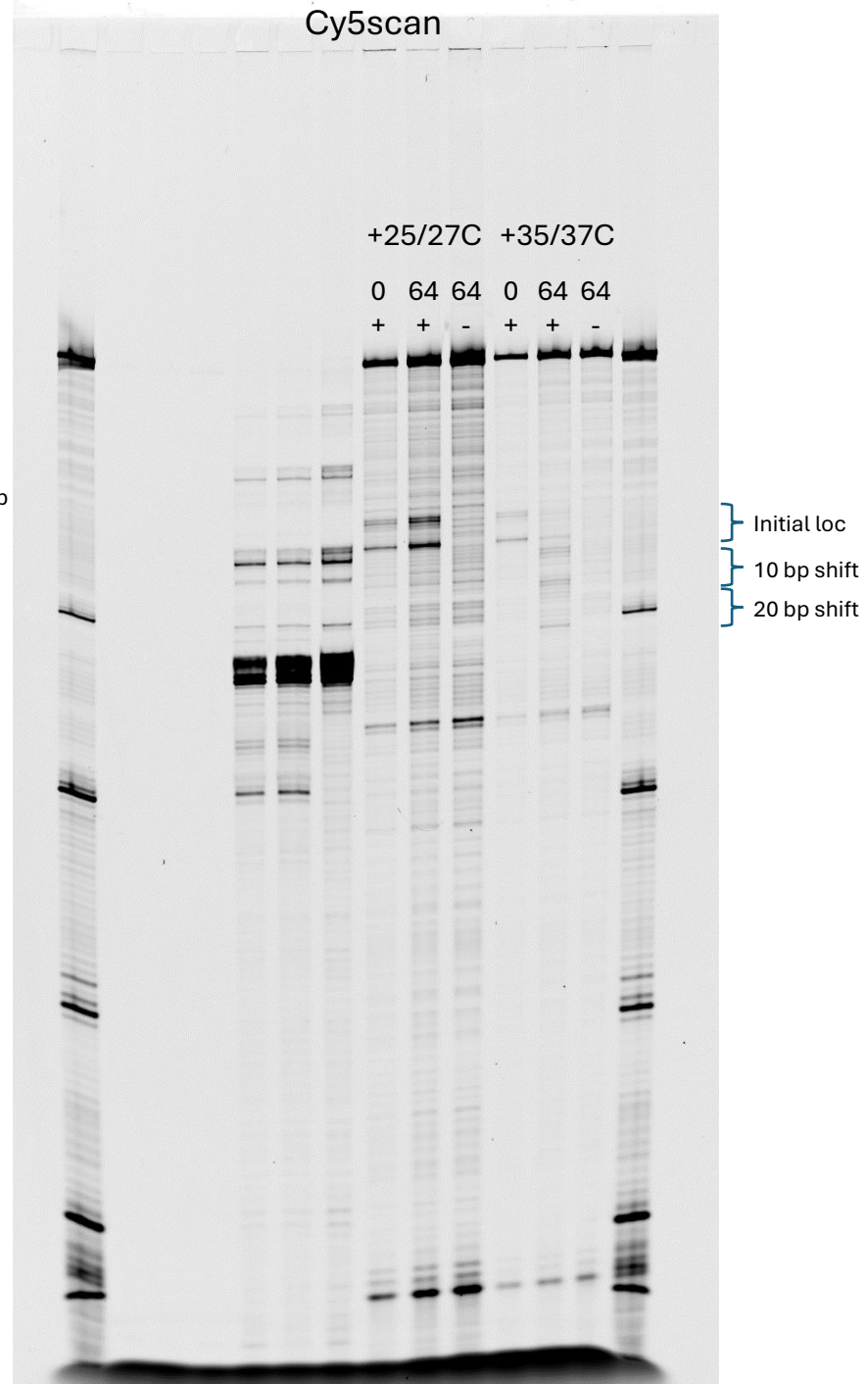

# Cy3scan

|          | -24/22C |    |   | -14/12C |    |   | -4/2C |    |   | +5/7C |    |   | +15/17C |    |   |
|----------|---------|----|---|---------|----|---|-------|----|---|-------|----|---|---------|----|---|
| Time:    | 32      | 32 | 0 | 32      | 32 | 0 | 32    | 32 | 0 | 32    | 32 | 0 | 32      | 32 | 0 |
| Mapping: | -       | +  | + | -       | +  | + | -     | +  | + | -     | +  | + | -       | +  | + |

Initial loc {  
 10 bp shift {  
 20bp shift {  
 25 bp shift {

126bp

95bp

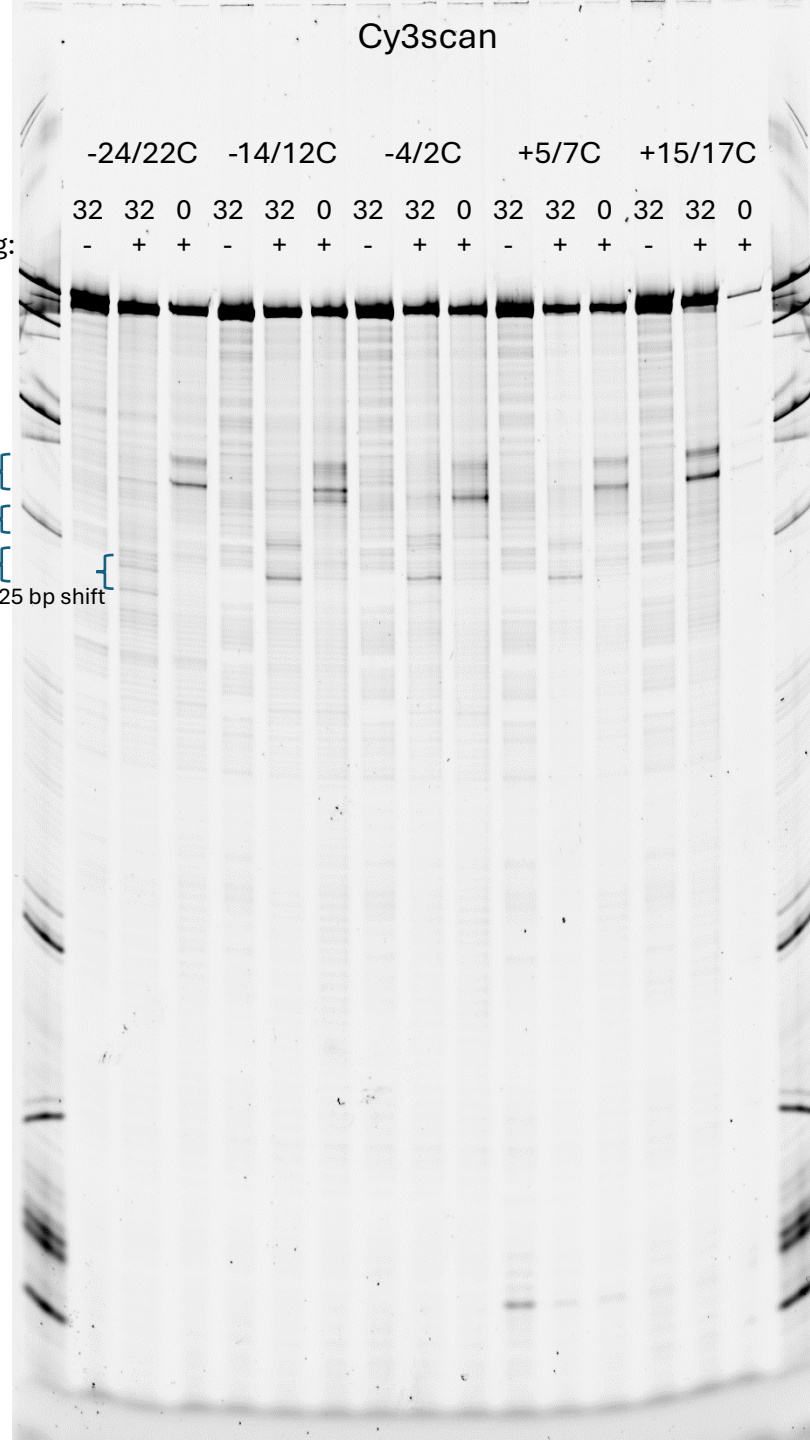

Cy5scan

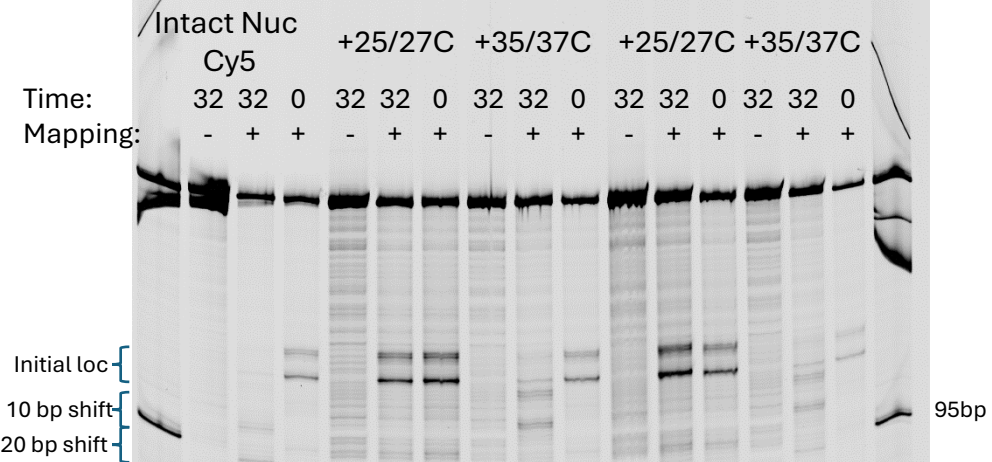

95bp

68bp

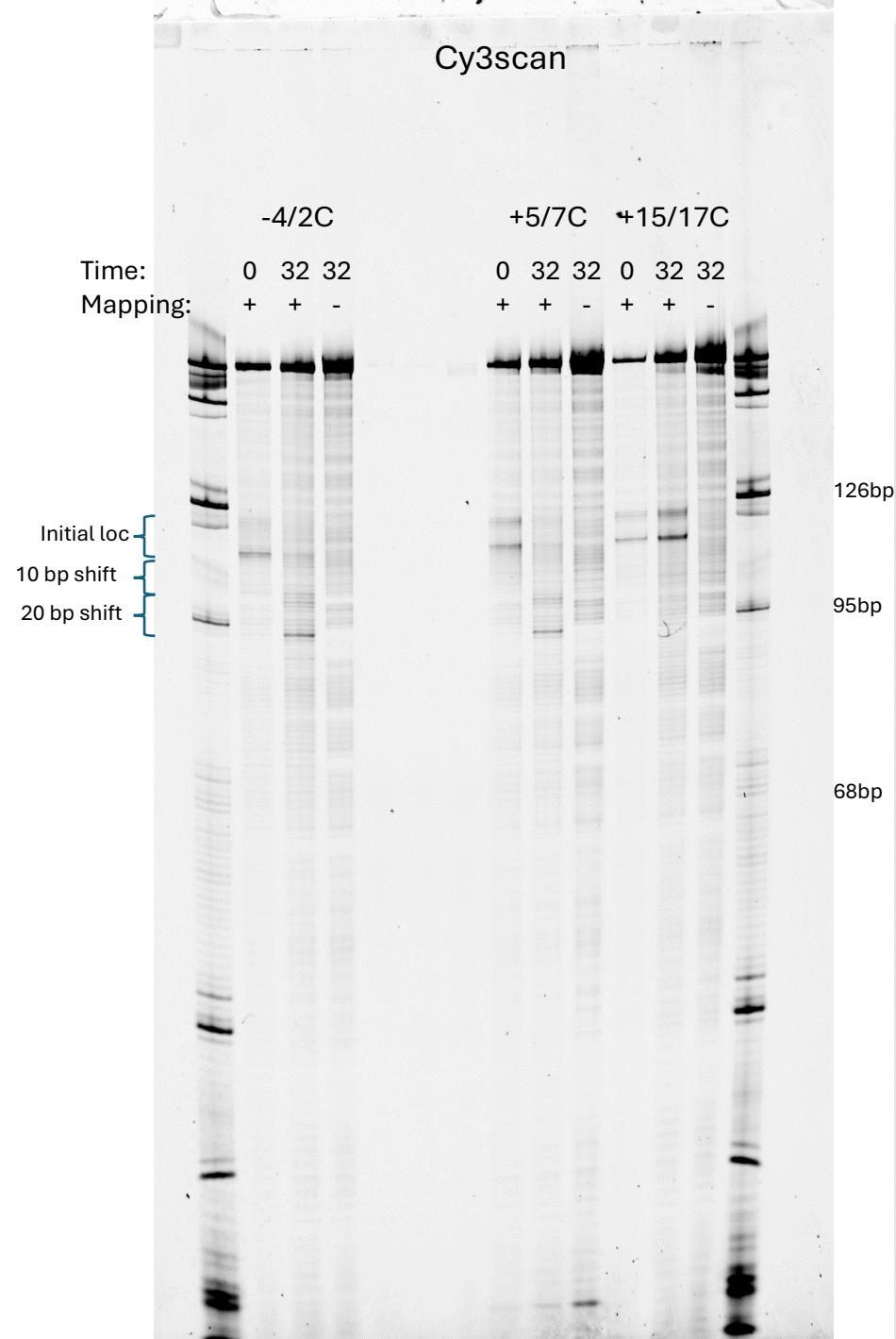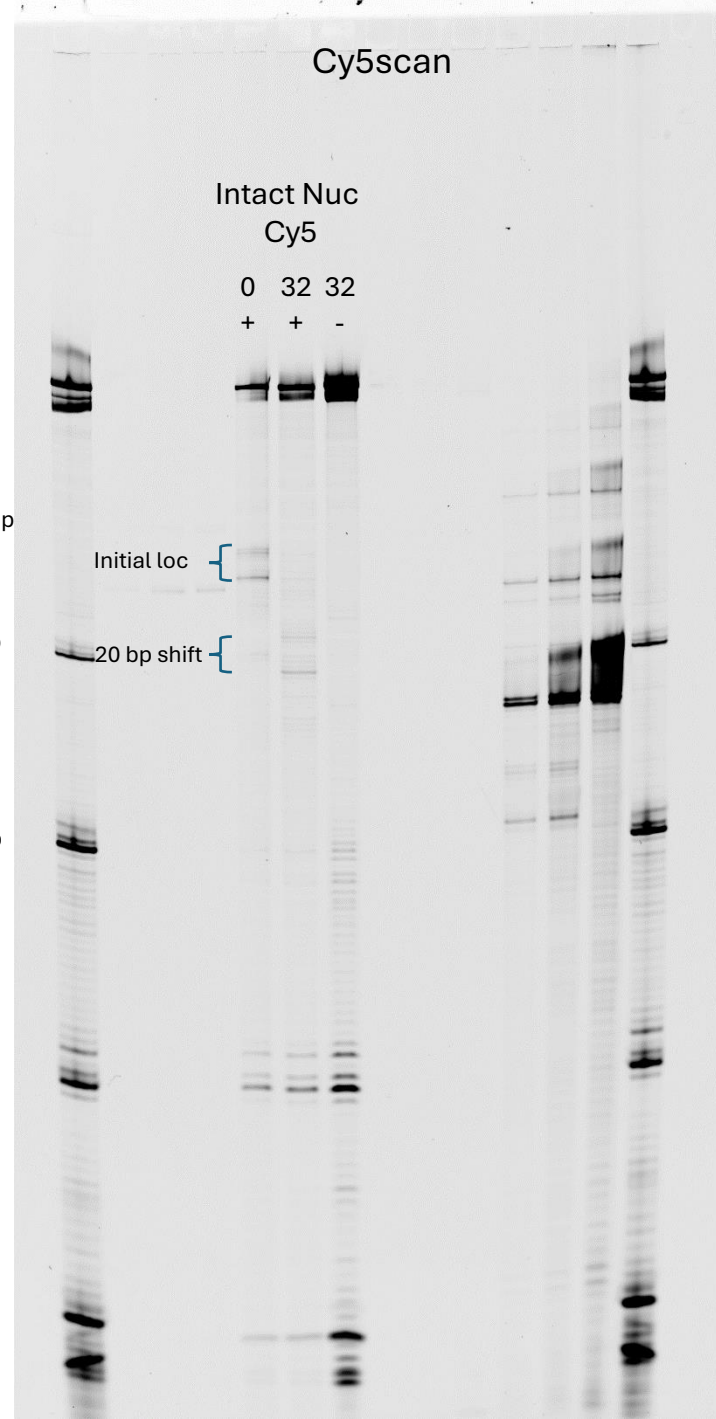

# Cy3scan

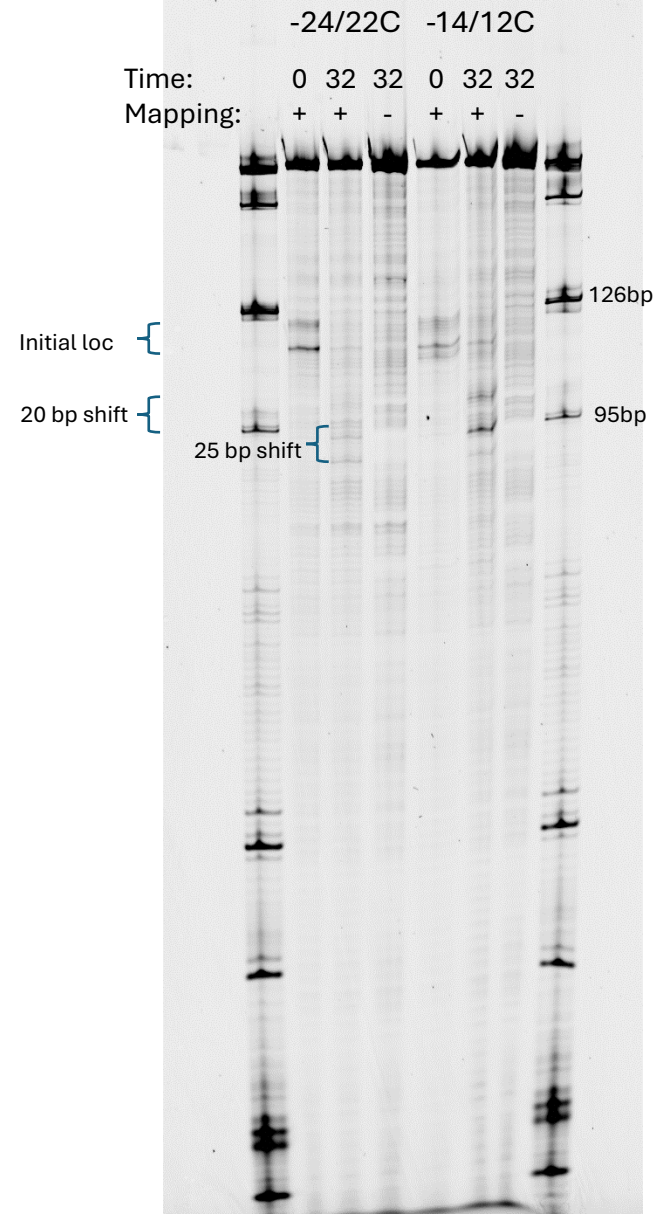

Supplement: Figure 1—source data 2. [file elife-52513-fig1-data2.zip › Figure1-source_data-labelled.pdf]

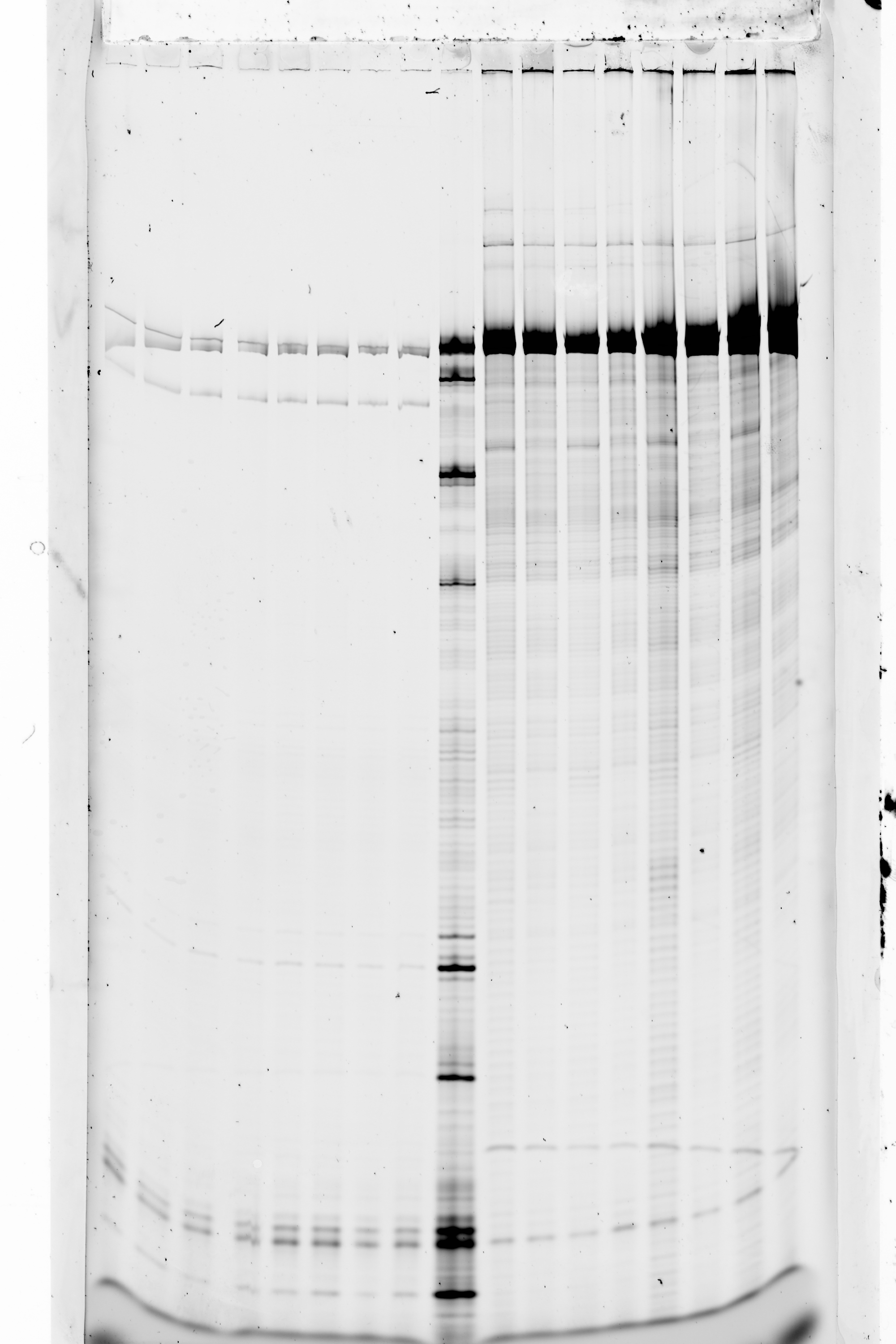

Supplement: Figure 2—source data 1. [file elife-52513-fig2-data1.zip › Figure2-sourcedata-Original/Chd1MAPscans/25Sept2018SHL-+2Chd1MapADP_ATP-Cy3.tif]

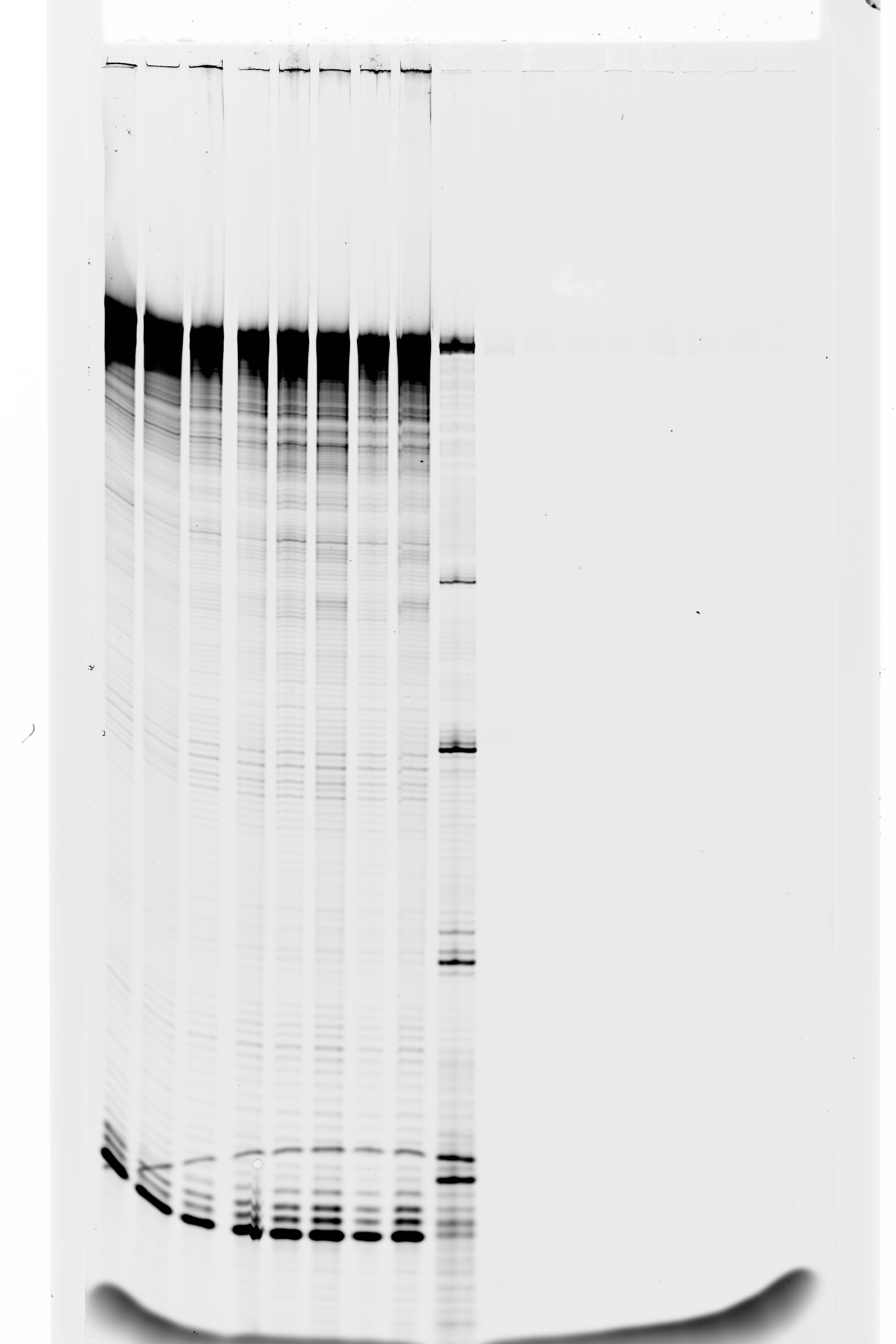

Supplement: Figure 2—source data 1. [file elife-52513-fig2-data1.zip › Figure2-sourcedata-Original/Chd1MAPscans/25Sept2018SHL-+2Chd1MapADP_ATP-Cy5.tif]

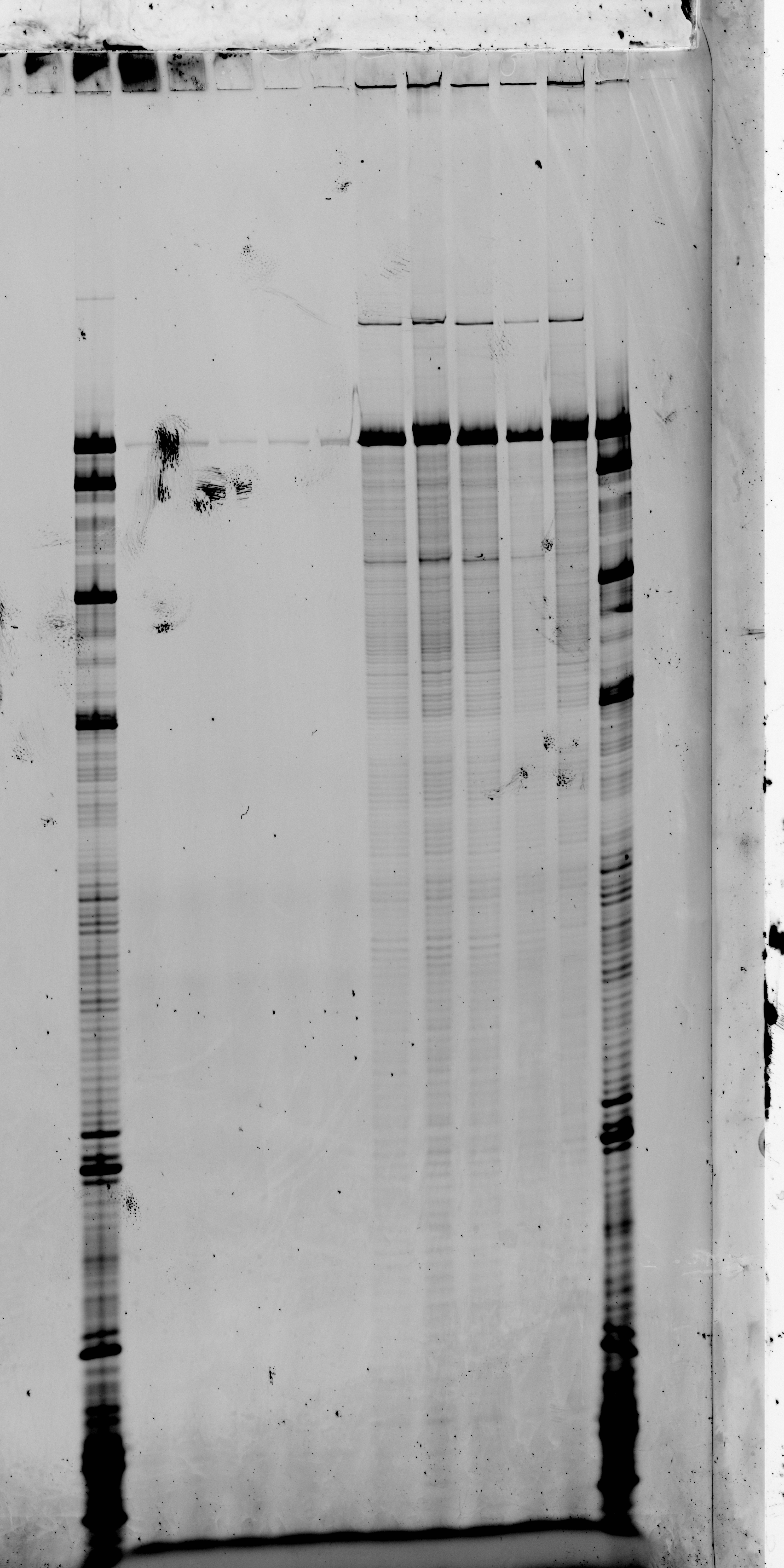

Supplement: Figure 2—source data 1. [file elife-52513-fig2-data1.zip › Figure2-sourcedata-Original/Chd1MAPscans/8Nov2018SHL+-2blockyChd1524map-Cy3.tif]

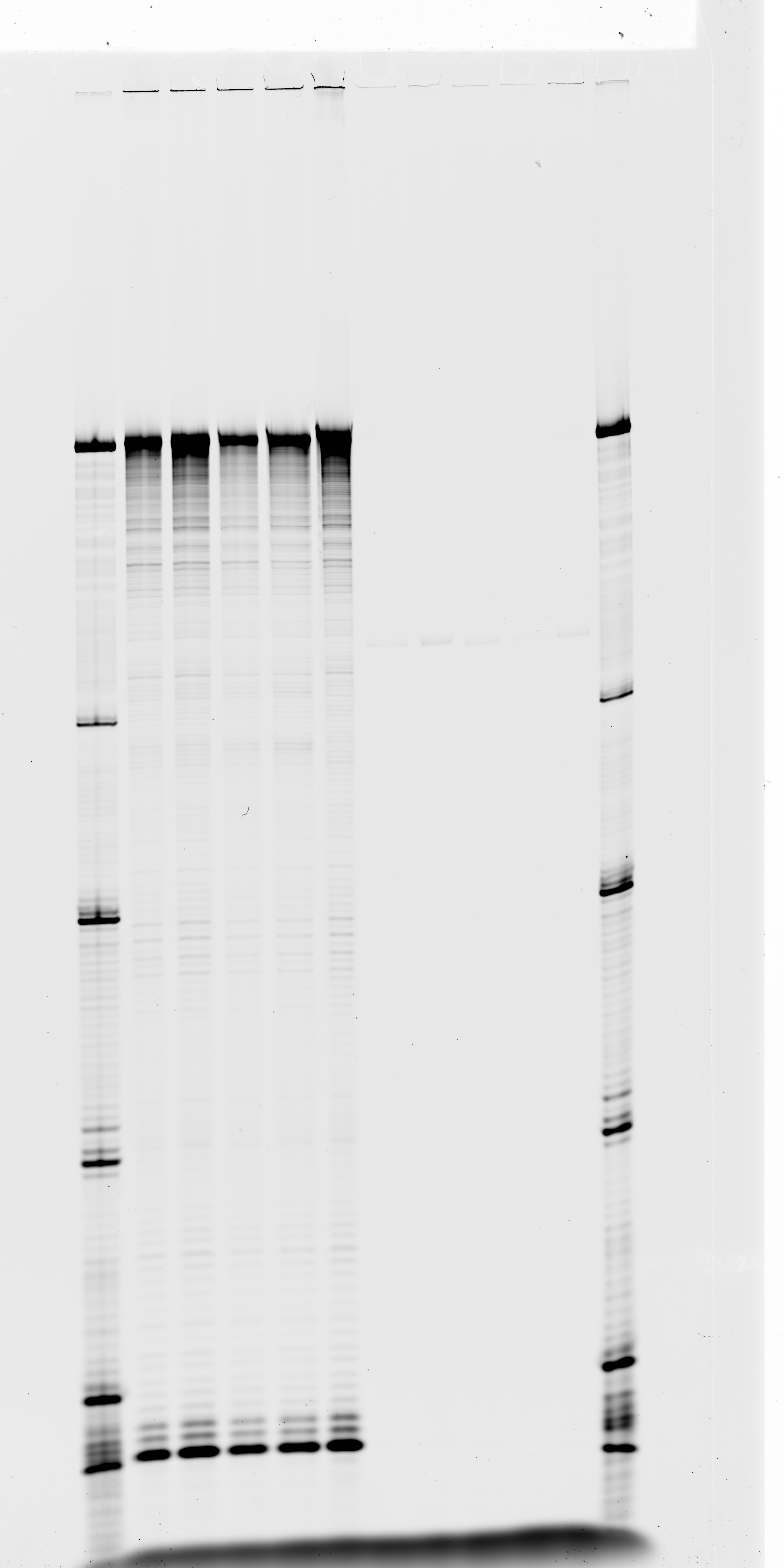

Supplement: Figure 2—source data 1. [file elife-52513-fig2-data1.zip › Figure2-sourcedata-Original/Chd1MAPscans/8Nov2018SHL+-2blockyChd1524map-Cy5.tif]

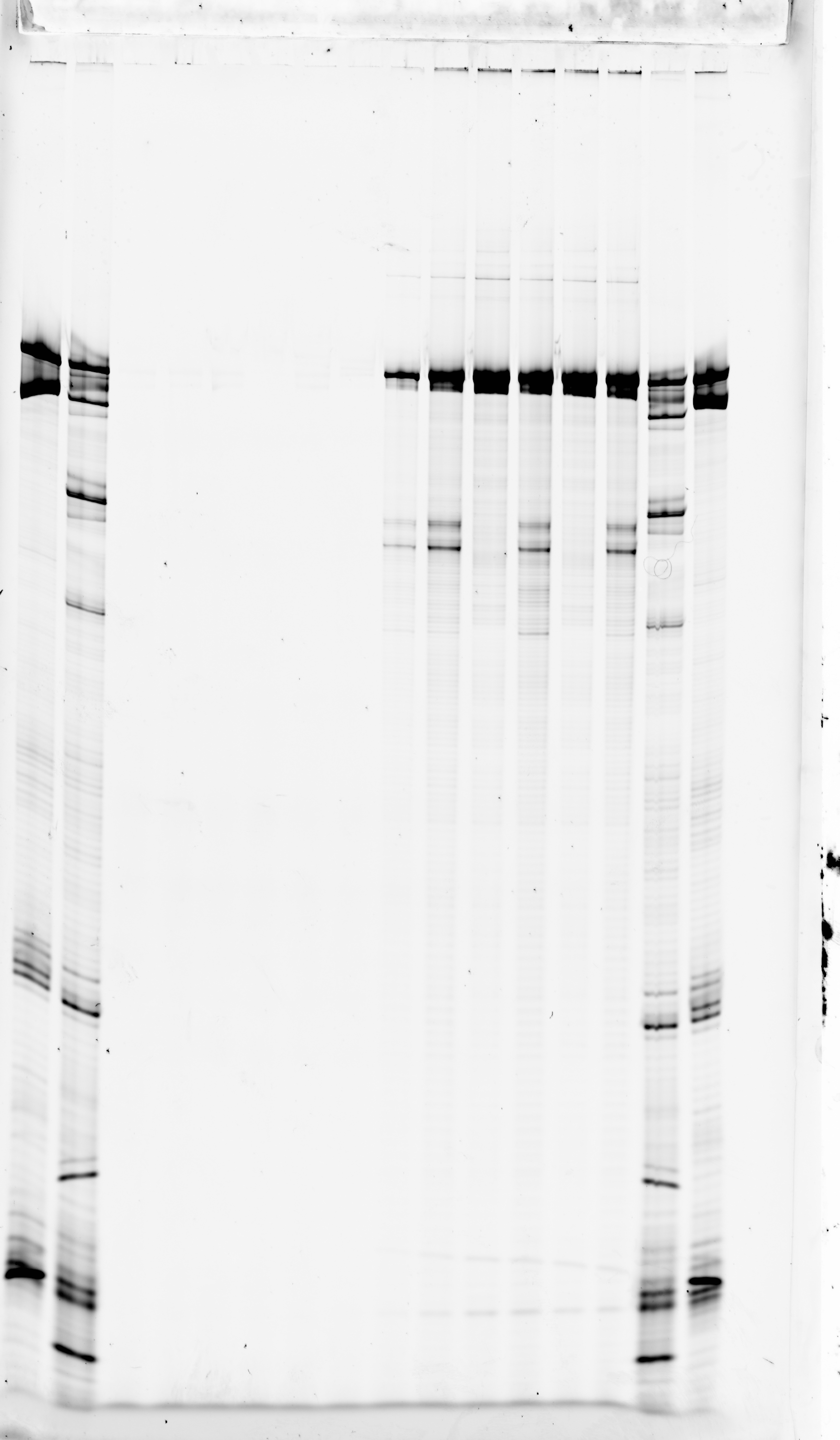

Supplement: Figure 2—source data 1. [file elife-52513-fig2-data1.zip › Figure2-sourcedata-Original/NucMAPscans/18Sept2018SHL+-2SAslideMAP-Cy3.tif]

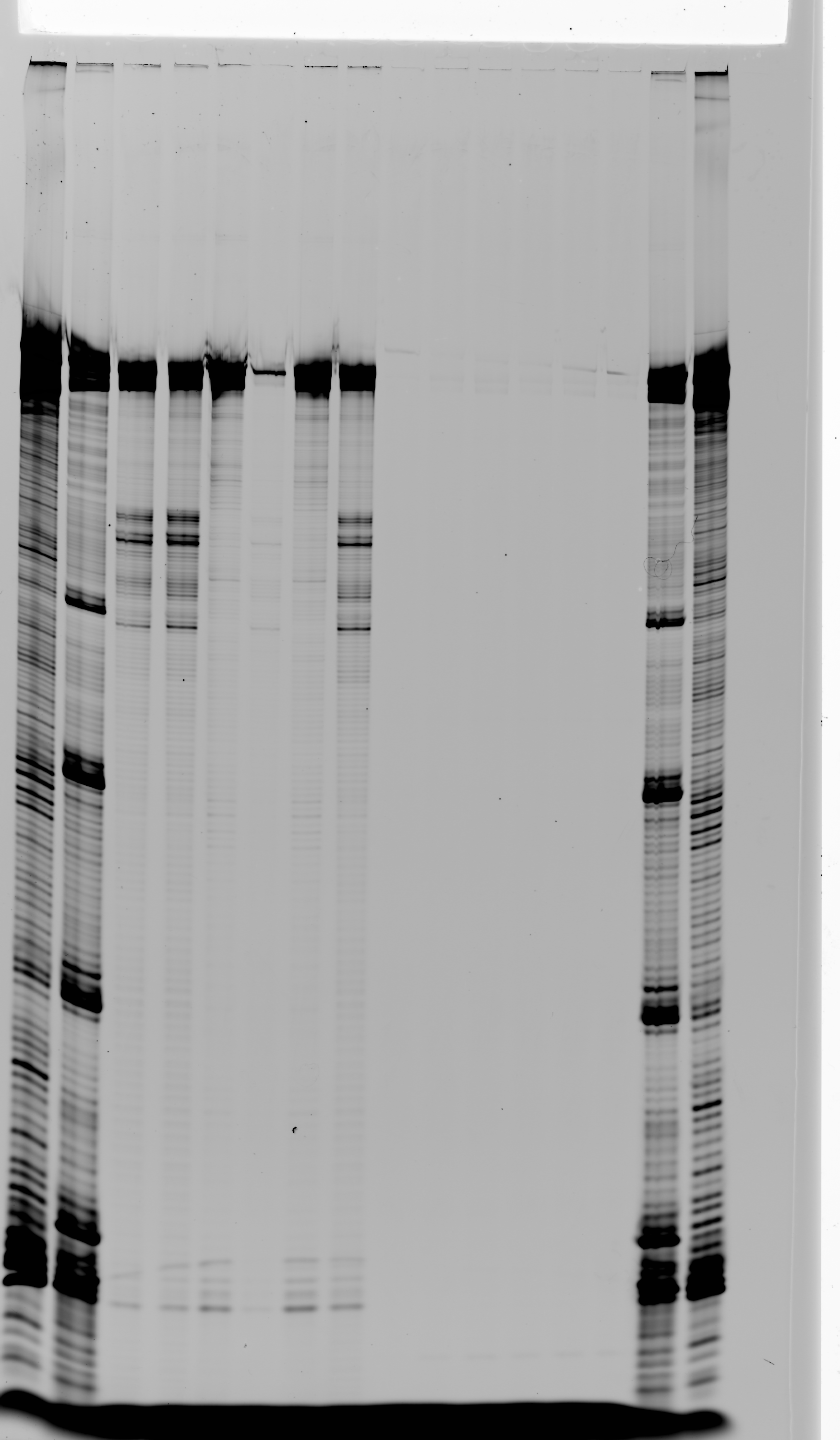

Supplement: Figure 2—source data 1. [file elife-52513-fig2-data1.zip › Figure2-sourcedata-Original/NucMAPscans/18Sept2018SHL+-2SAslideMAP-Cy5.tif]

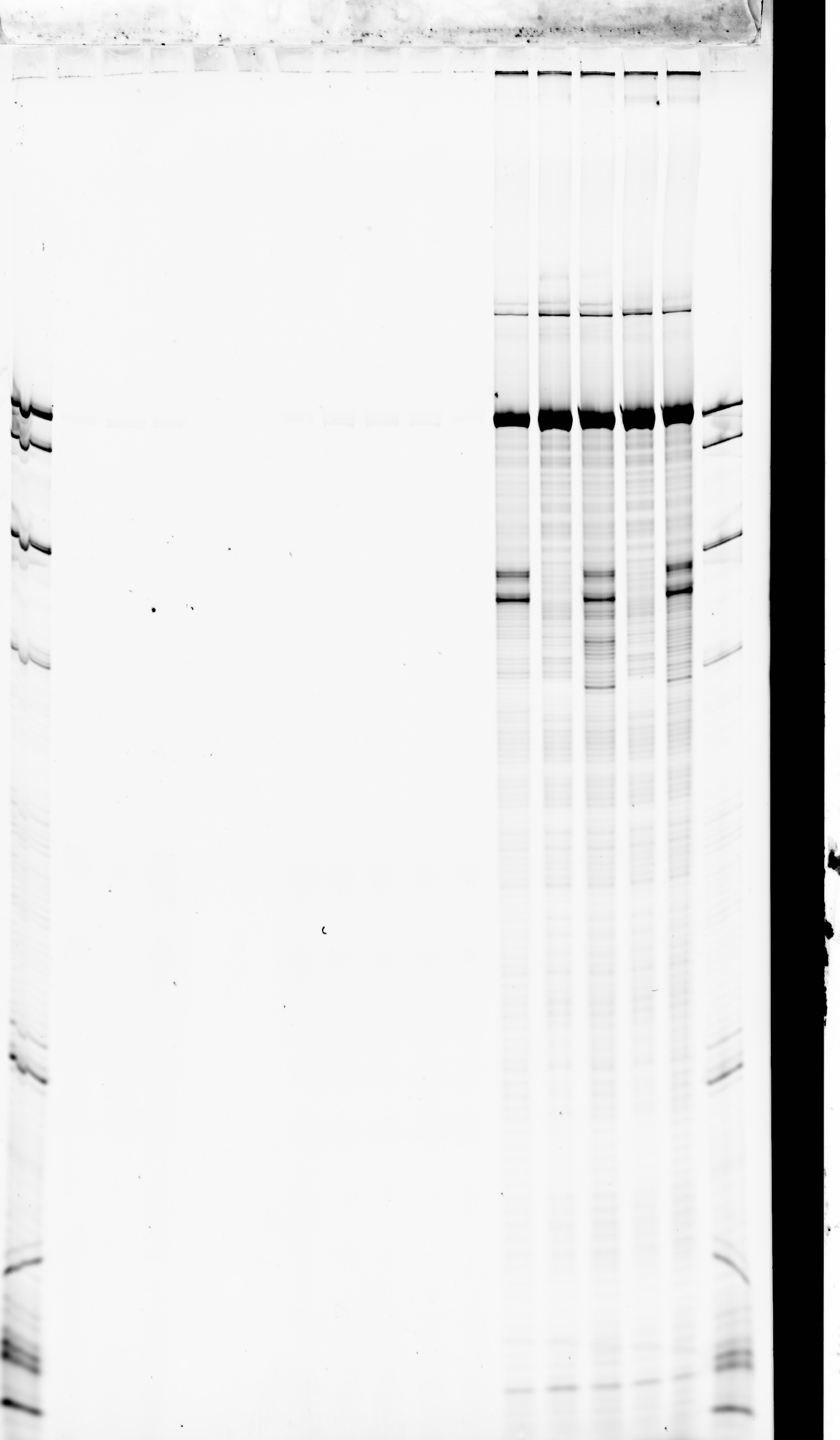

Supplement: Figure 2—source data 1. [file elife-52513-fig2-data1.zip › Figure2-sourcedata-Original/NucMAPscans/31MAy2018SHL+-2SAmapping-[Cy3].tif]

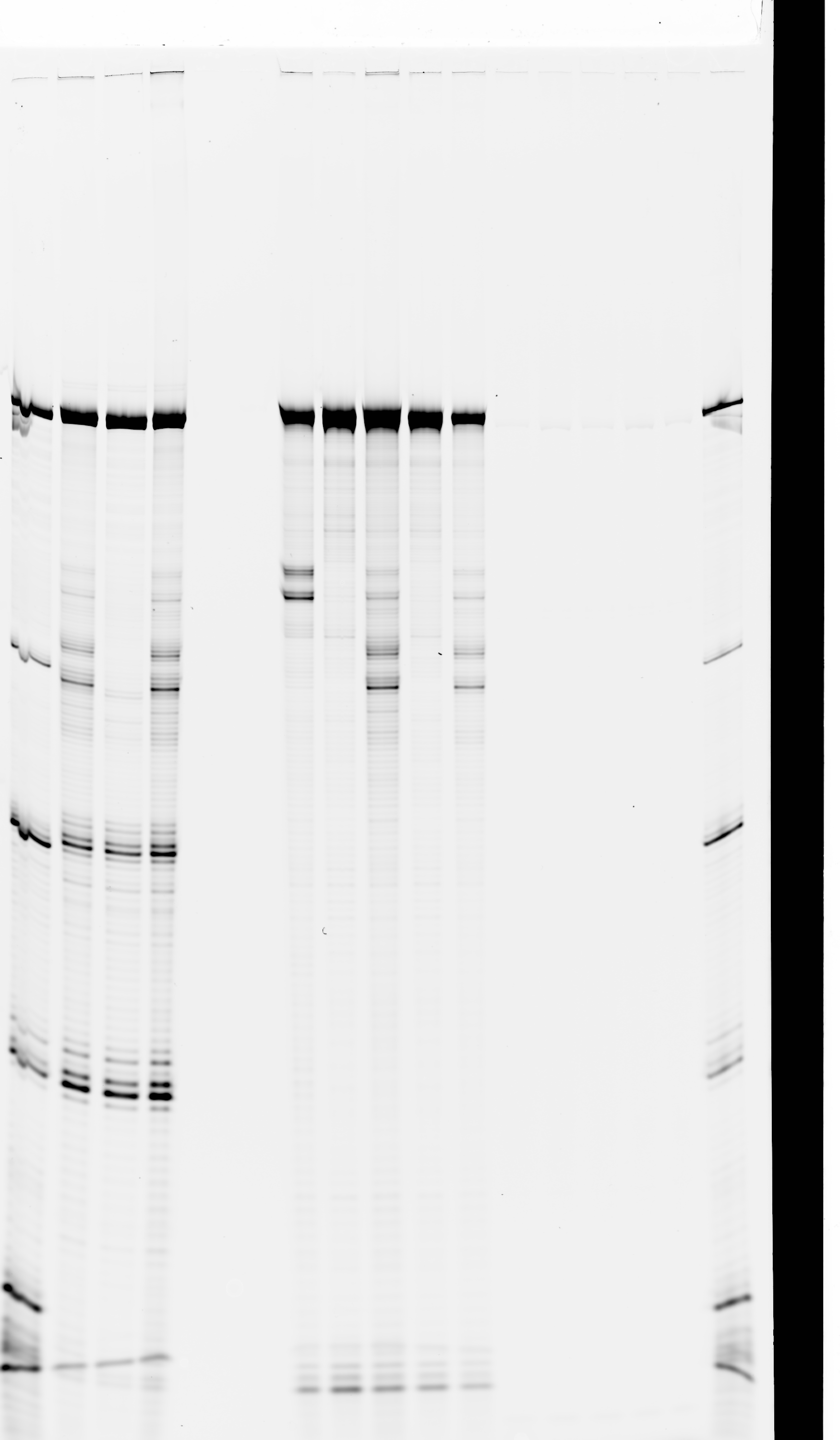

Supplement: Figure 2—source data 1. [file elife-52513-fig2-data1.zip › Figure2-sourcedata-Original/NucMAPscans/31MAy2018SHL+-2SAmapping-[Cy5].tif]

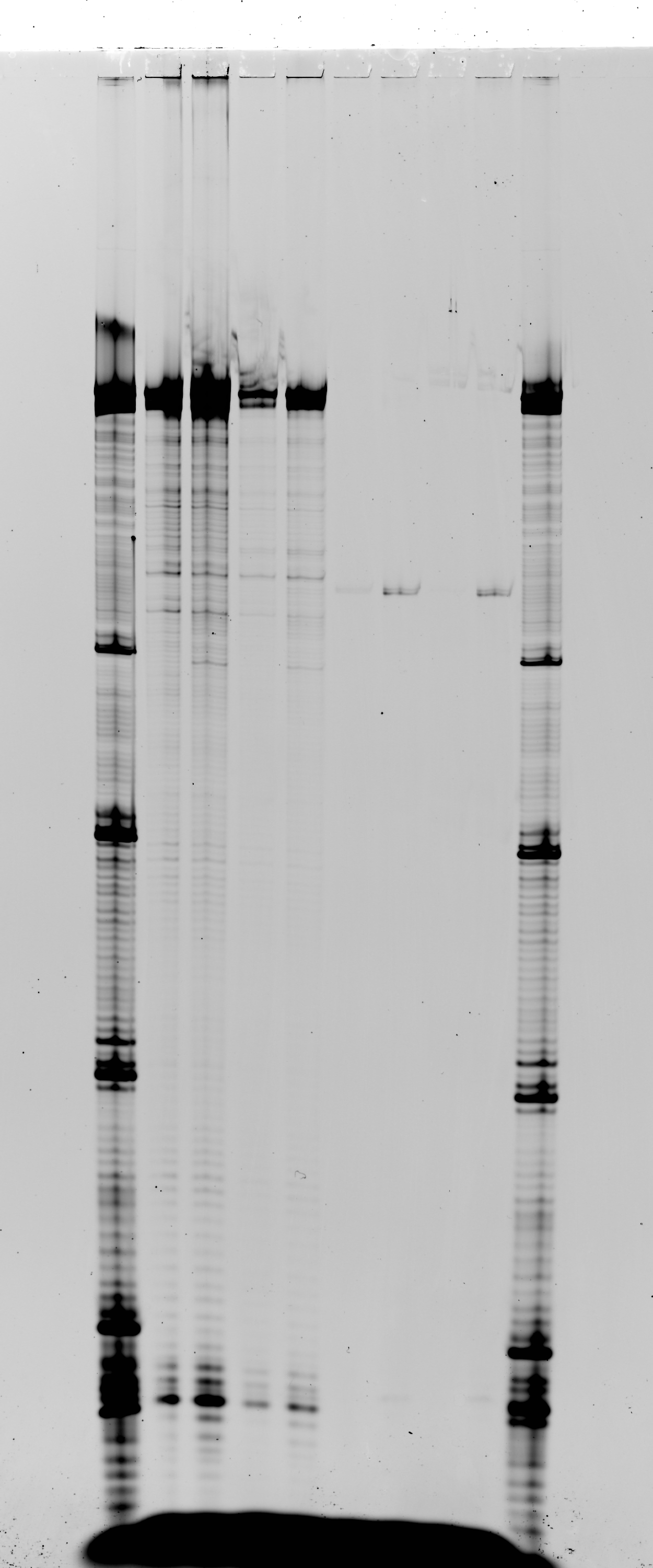

Supplement: Figure 2—source data 1. [file elife-52513-fig2-data1.zip › Figure2-sourcedata-Original/NucMAPscans/8Nov2018SHL+-2blockNucMap-Cy3.tif]

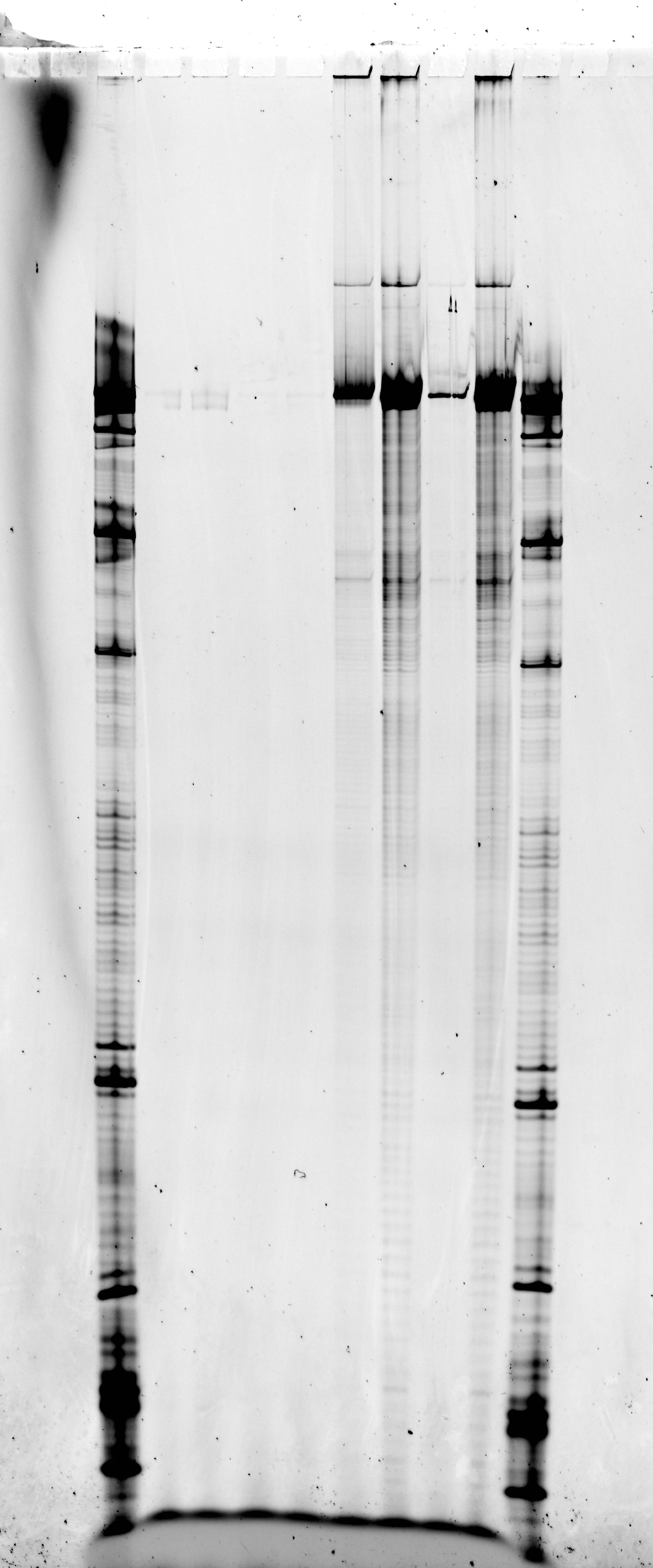

Supplement: Figure 2—source data 1. [file elife-52513-fig2-data1.zip › Figure2-sourcedata-Original/NucMAPscans/8Nov2018SHL+-2blockNucMap-Cy5.tif]

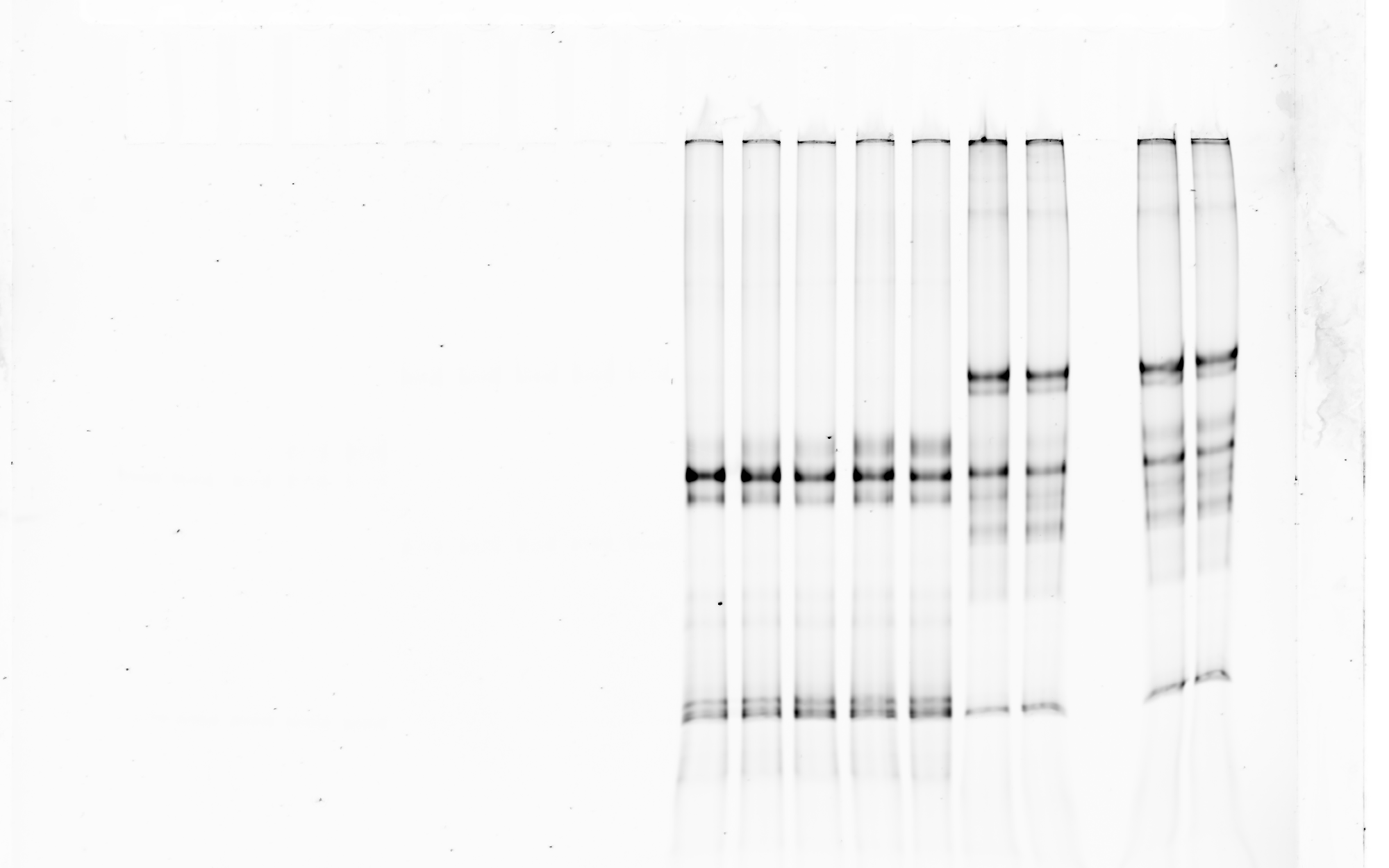

Supplement: Figure 2—source data 1. [file elife-52513-fig2-data1.zip › Figure2-sourcedata-Original/NucSlideScans/14Sept2018+-SHL2blockSlide-Cy3.tif]

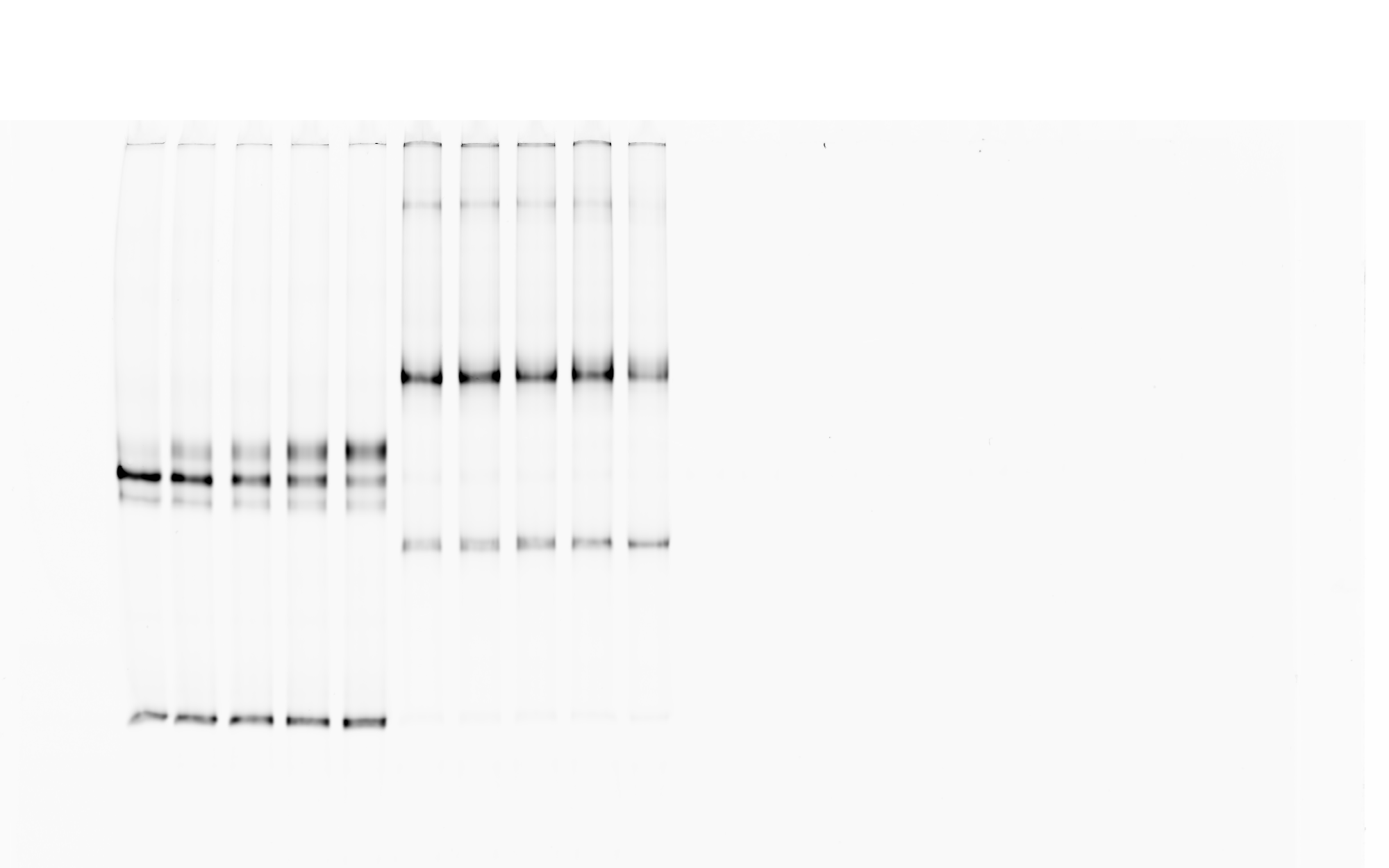

Supplement: Figure 2—source data 1. [file elife-52513-fig2-data1.zip › Figure2-sourcedata-Original/NucSlideScans/14Sept2018+-SHL2blockSlide-Cy5.tif]

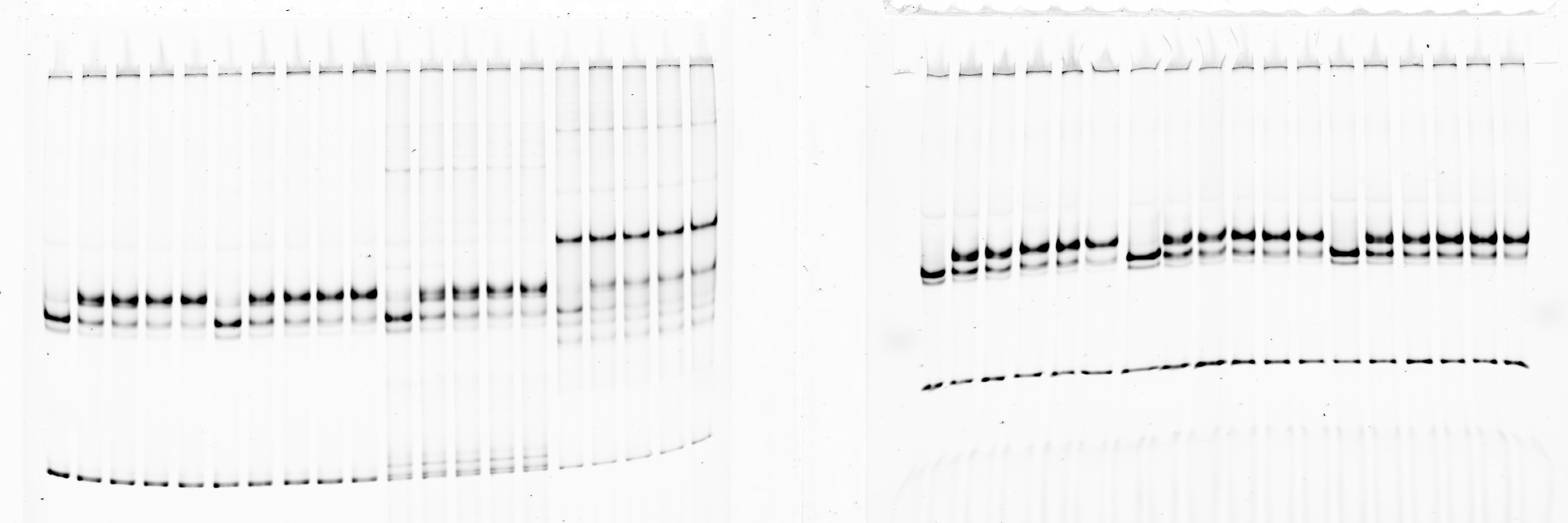

Supplement: Figure 2—source data 1. [file elife-52513-fig2-data1.zip › Figure2-sourcedata-Original/NucSlideScans/23May2018SHL2bio+SA_H3tailSlides-[Cy3].tif]

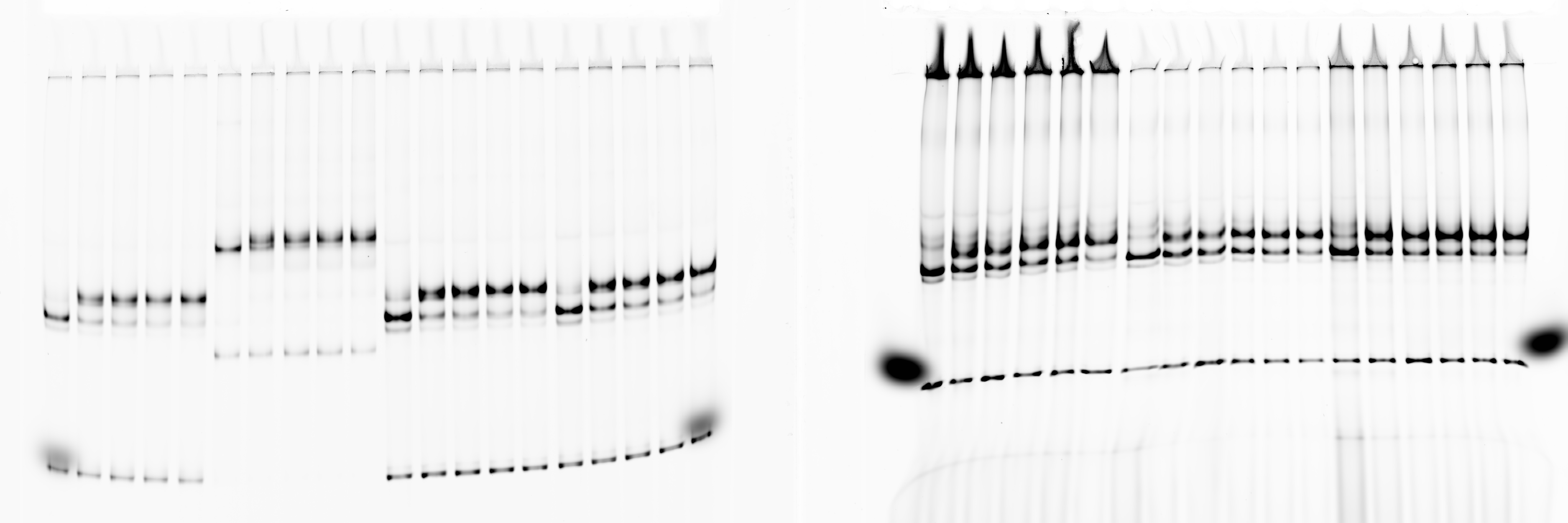

Supplement: Figure 2—source data 1. [file elife-52513-fig2-data1.zip › Figure2-sourcedata-Original/NucSlideScans/23May2018SHL2bio+SA_H3tailSlides-[Cy5].tif]

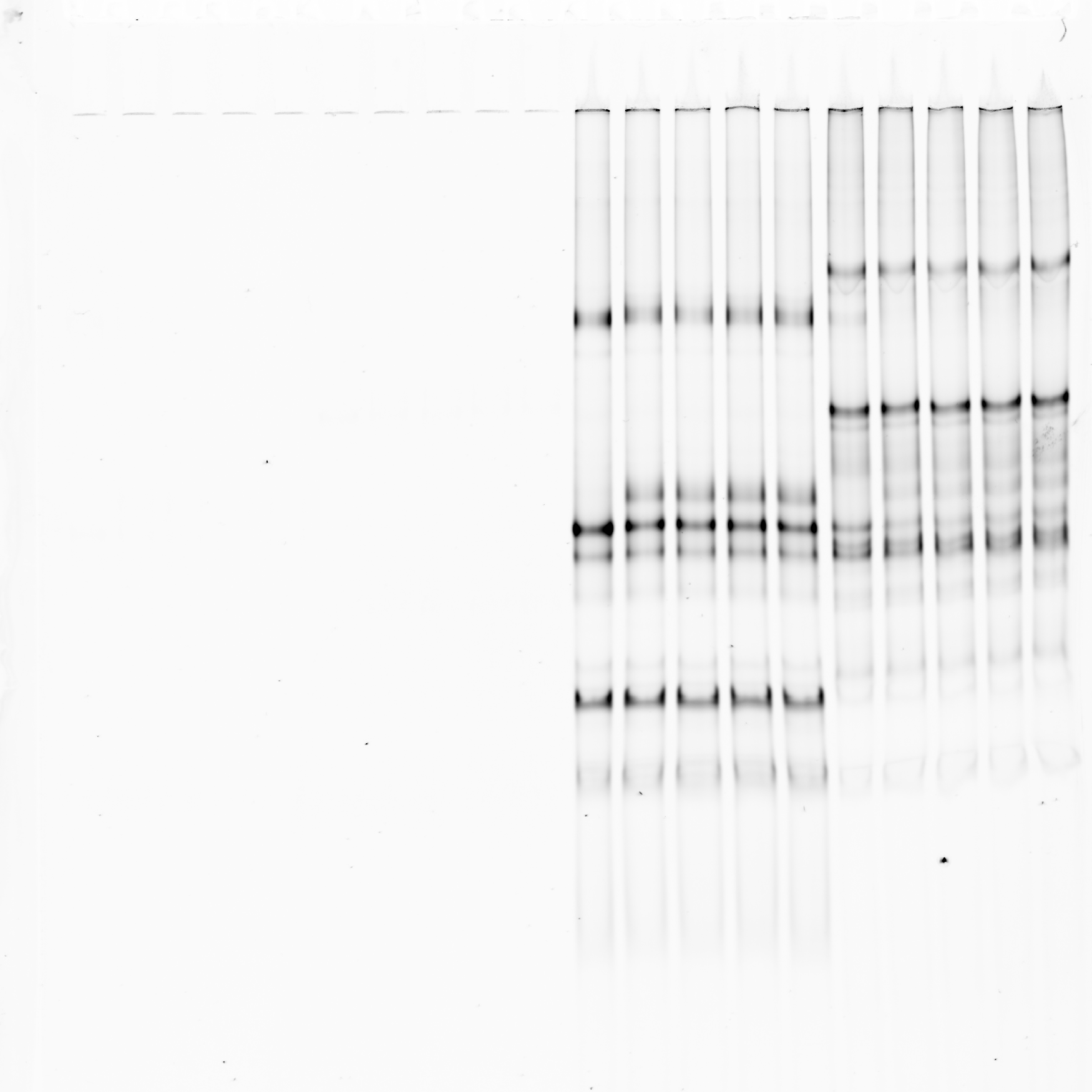

Supplement: Figure 2—source data 1. [file elife-52513-fig2-data1.zip › Figure2-sourcedata-Original/NucSlideScans/7Nov2018xNucMAPslidingSHL+-2SAblock-Cy3.tif]

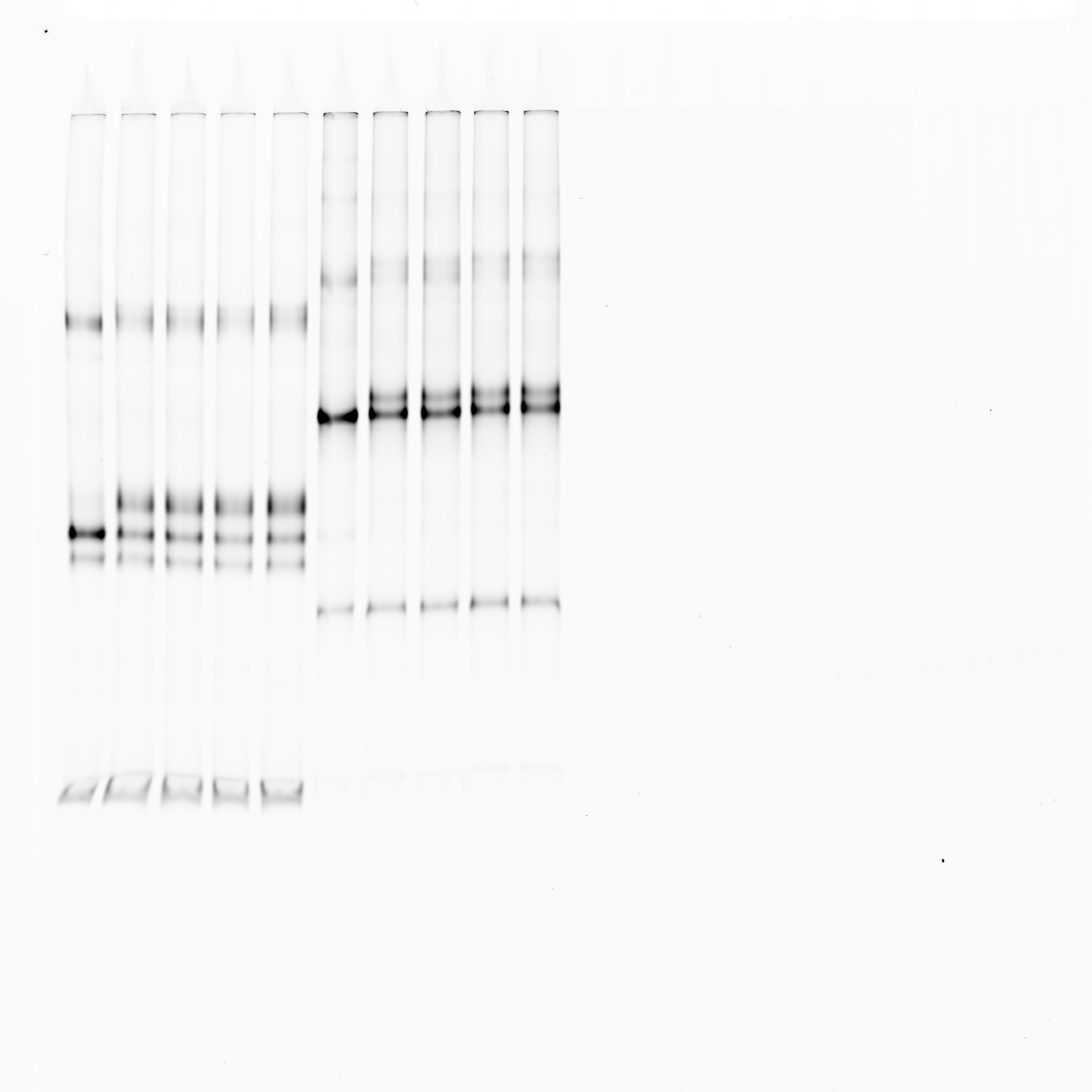

Supplement: Figure 2—source data 1. [file elife-52513-fig2-data1.zip › Figure2-sourcedata-Original/NucSlideScans/7Nov2018xNucMAPslidingSHL+-2SAblock-Cy5.tif]

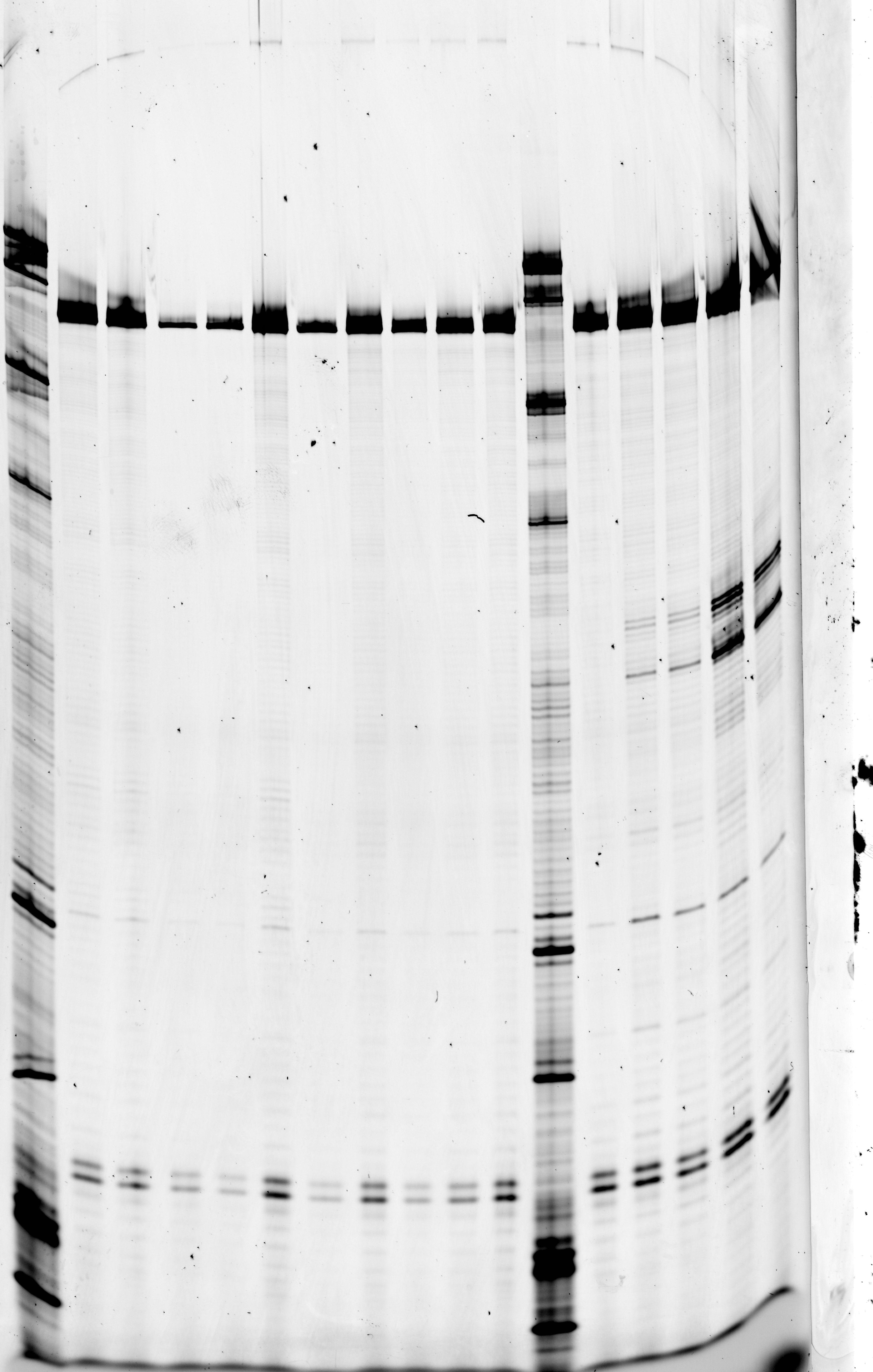

Supplement: Figure 4—source data 1. [file elife-52513-fig4-data1.zip › Figure4-sourcedata-original/Chd1MAPscans/19Dec2018xNuc1W0Chd1524_1127map_NucMap-Cy3.tif]

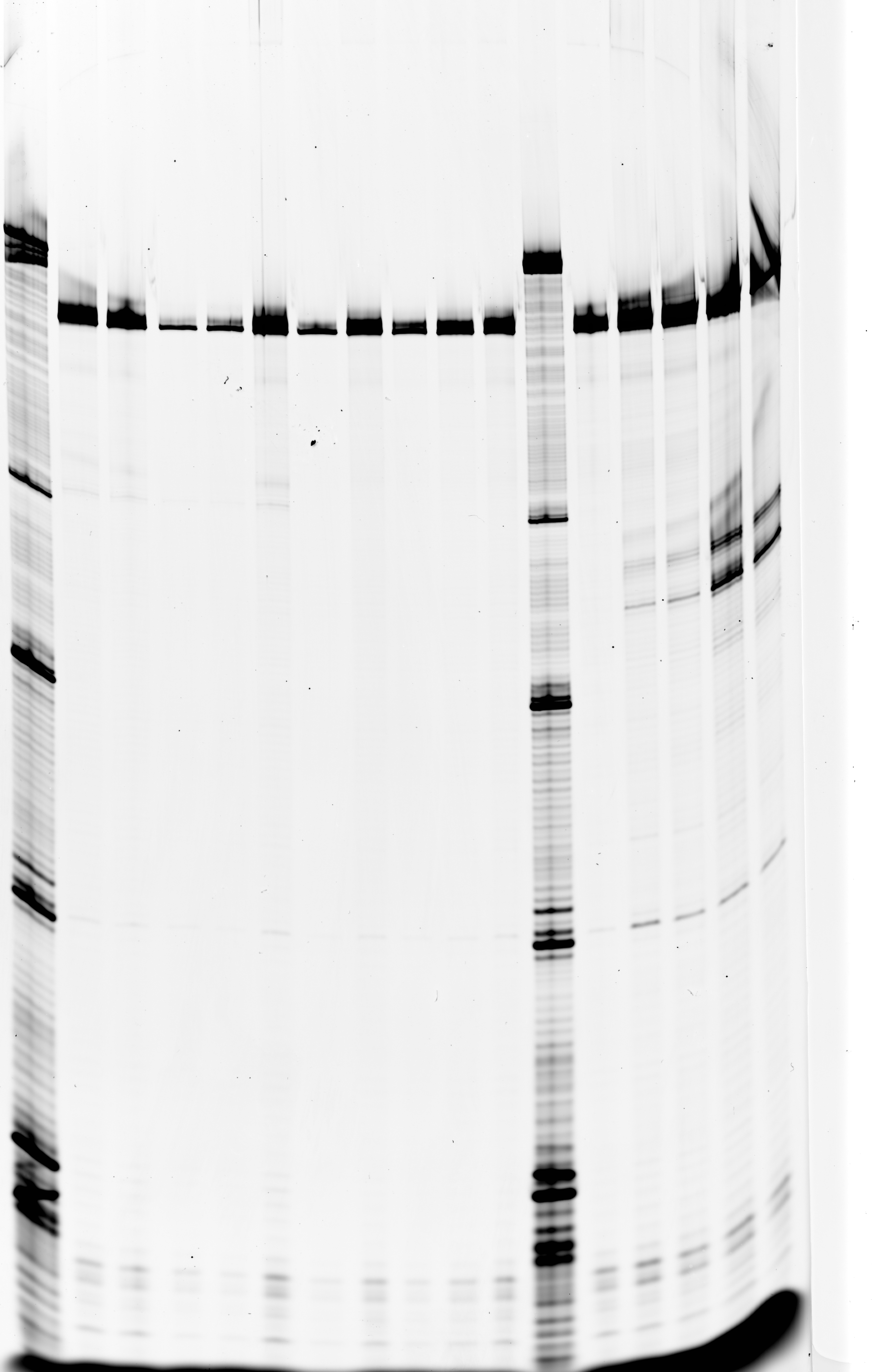

Supplement: Figure 4—source data 1. [file elife-52513-fig4-data1.zip › Figure4-sourcedata-original/Chd1MAPscans/19Dec2018xNuc1W0Chd1524_1127map_NucMap-Cy5.tif]

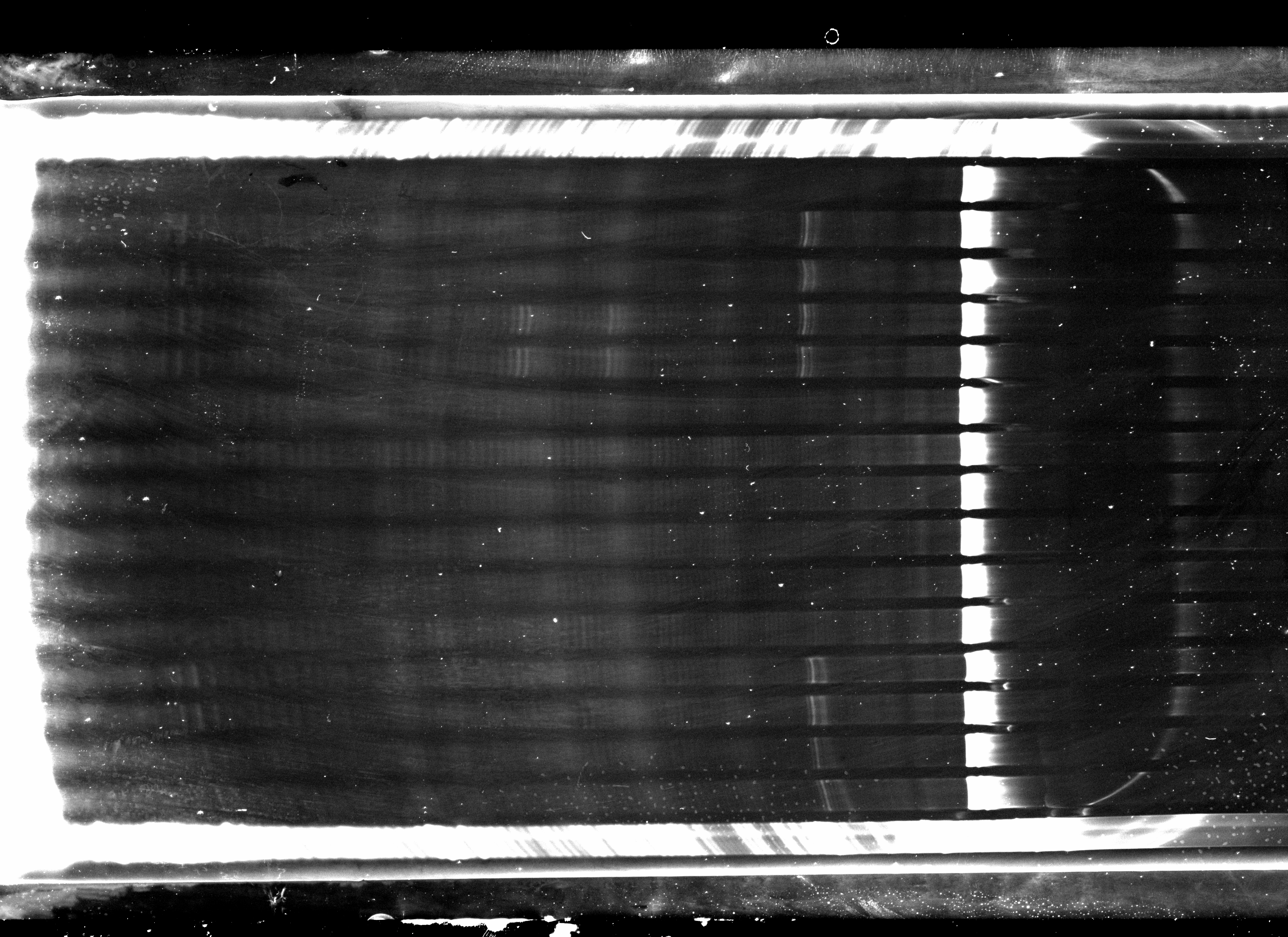

Supplement: Figure 4—source data 1. [file elife-52513-fig4-data1.zip › Figure4-sourcedata-original/Chd1MAPscans/28Nov2018xNuc0W11PAGEEyChd1map524_R804Q524_1127-Cy3.tif]

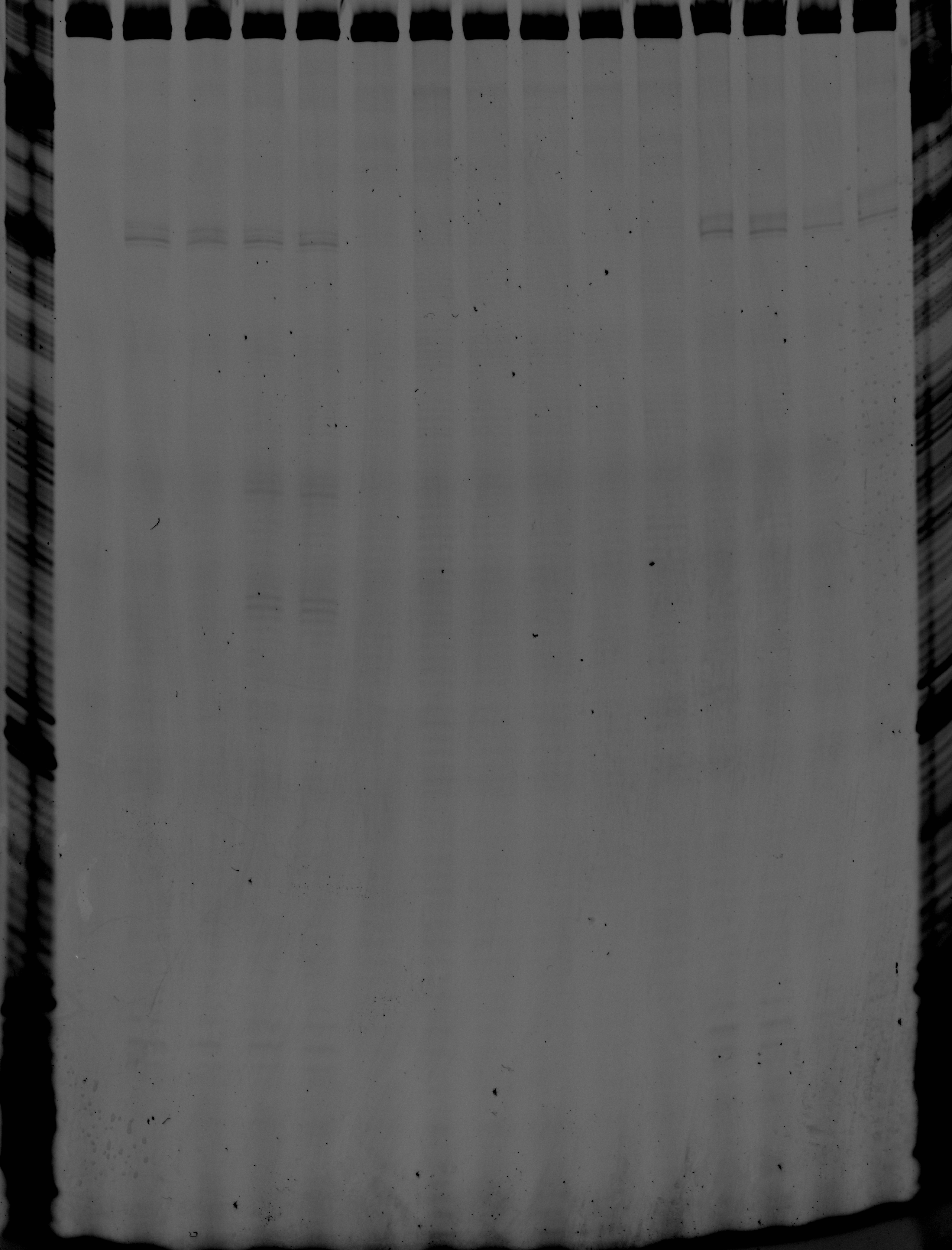

Supplement: Figure 4—source data 1. [file elife-52513-fig4-data1.zip › Figure4-sourcedata-original/Chd1MAPscans/28Nov2018xNuc0W11PAGEEyChd1map524_R804Q524_1127-Cy3hi.tif]

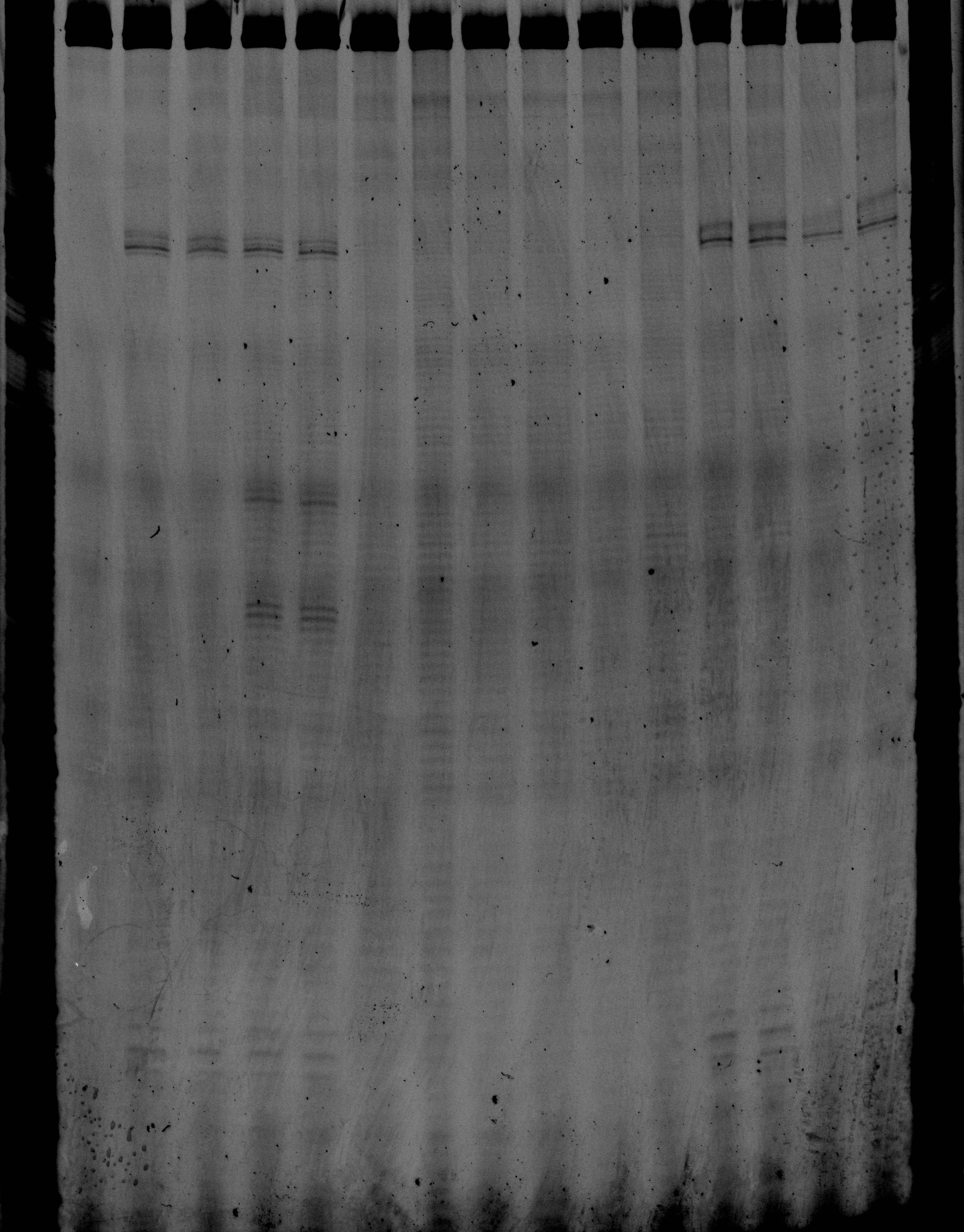

Supplement: Figure 4—source data 1. [file elife-52513-fig4-data1.zip › Figure4-sourcedata-original/Chd1MAPscans/28Nov2018xNuc0W11PAGEEyChd1map524_R804Q524_1127-Cy3over.tif]

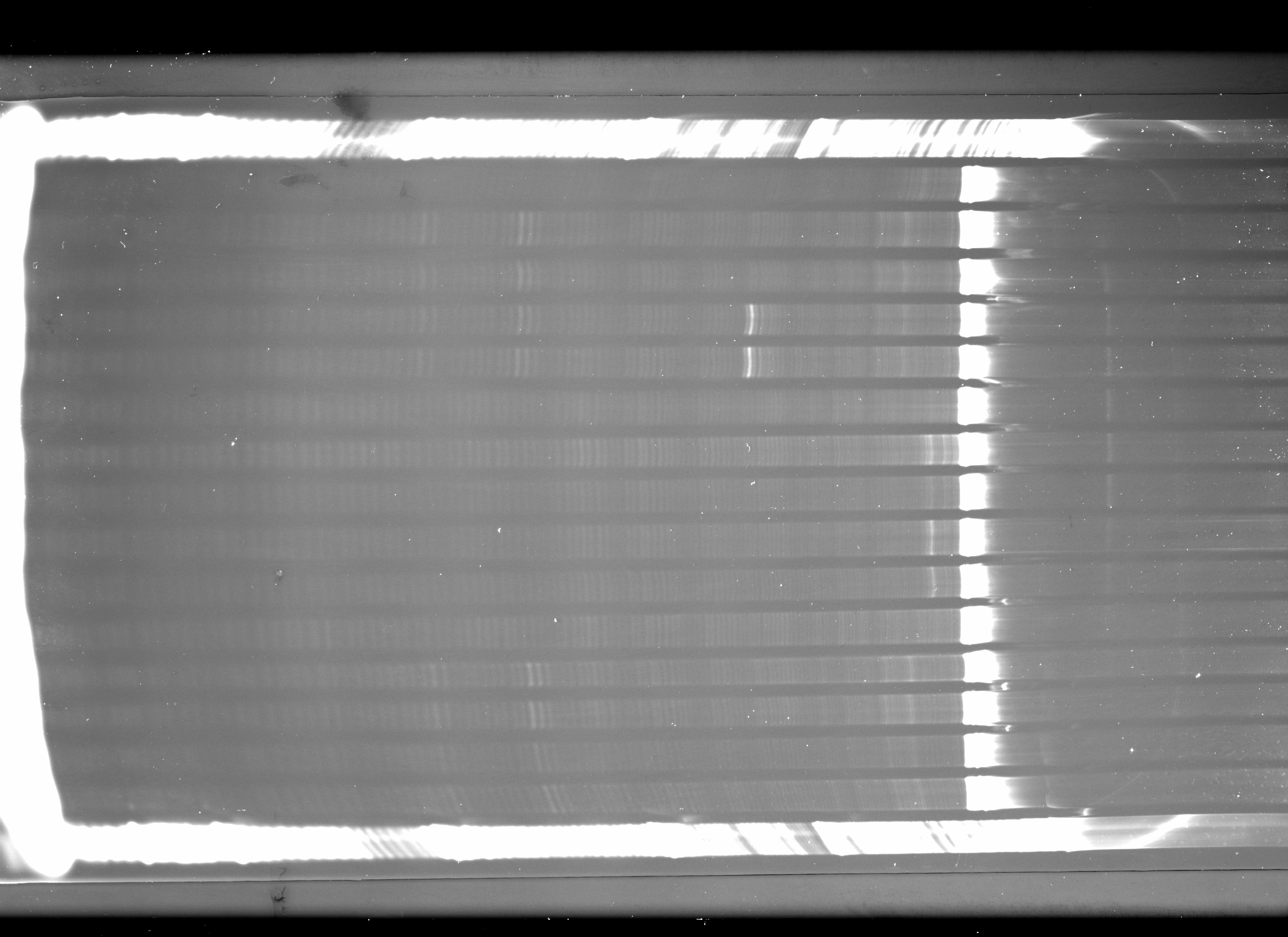

Supplement: Figure 4—source data 1. [file elife-52513-fig4-data1.zip › Figure4-sourcedata-original/Chd1MAPscans/28Nov2018xNuc0W11PAGEEyChd1map524_R804Q524_1127-Cy5.tif]

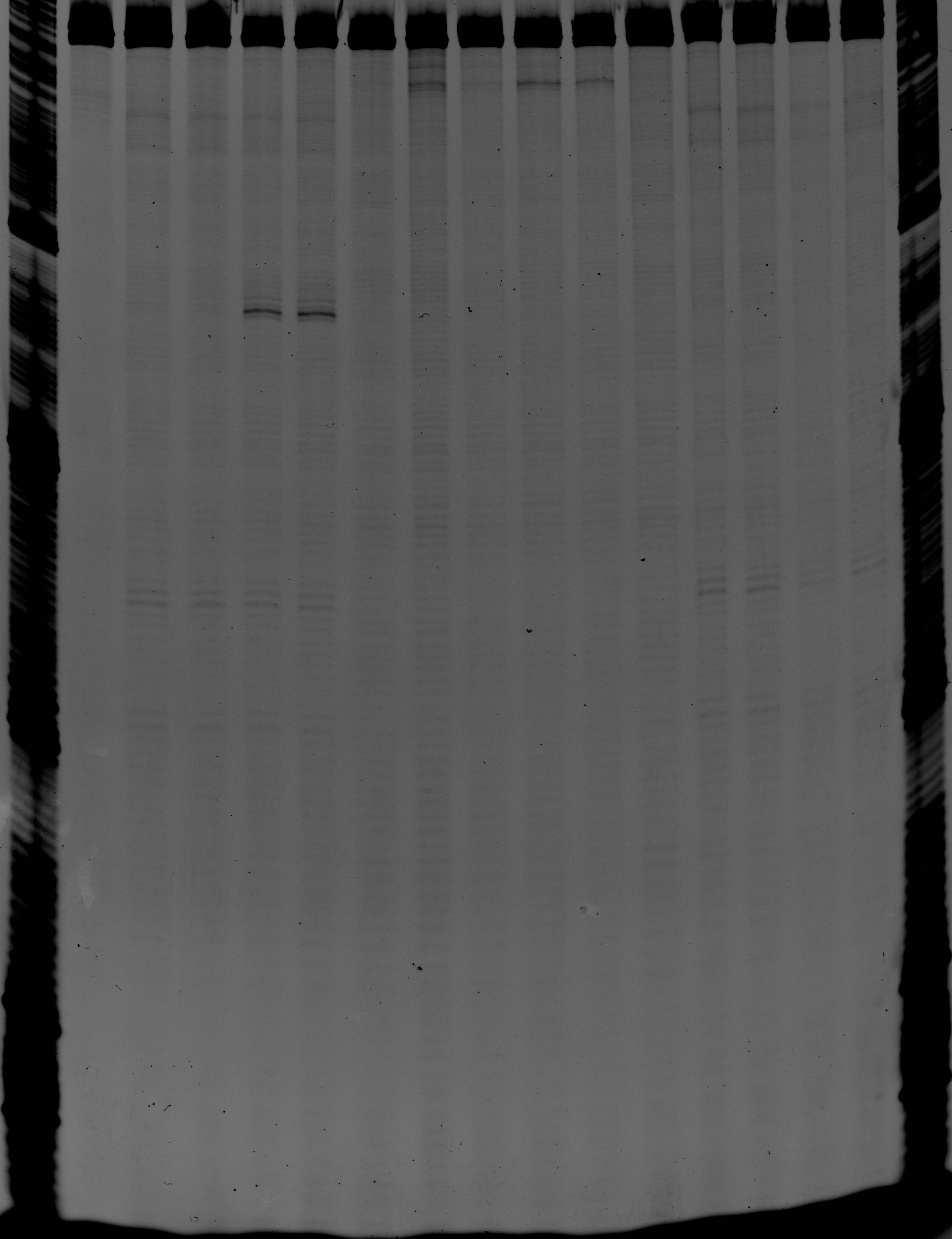

Supplement: Figure 4—source data 1. [file elife-52513-fig4-data1.zip › Figure4-sourcedata-original/Chd1MAPscans/28Nov2018xNuc0W11PAGEEyChd1map524_R804Q524_1127-Cy5hi.tif]

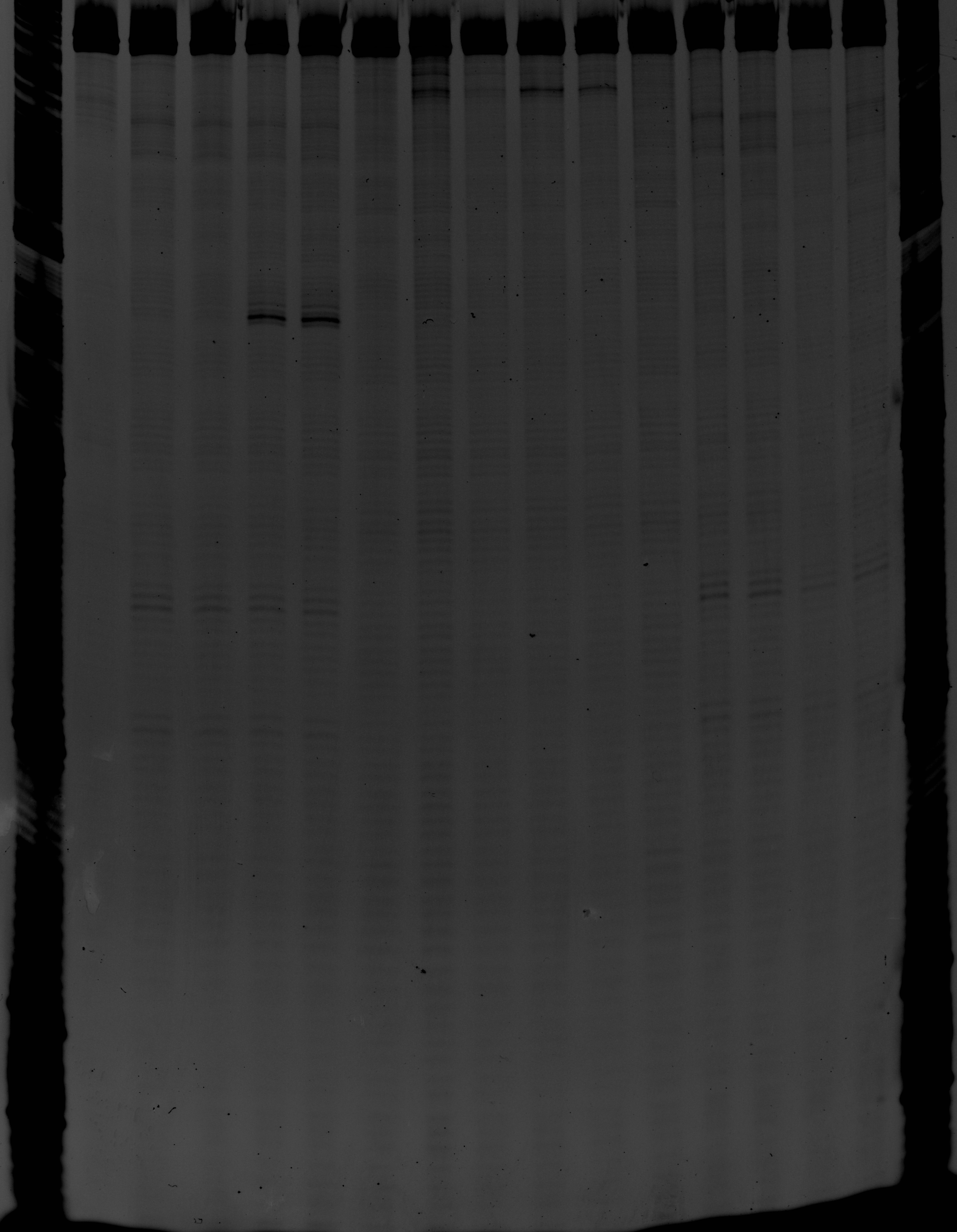

Supplement: Figure 4—source data 1. [file elife-52513-fig4-data1.zip › Figure4-sourcedata-original/Chd1MAPscans/28Nov2018xNuc0W11PAGEEyChd1map524_R804Q524_1127-Cy5over.tif]

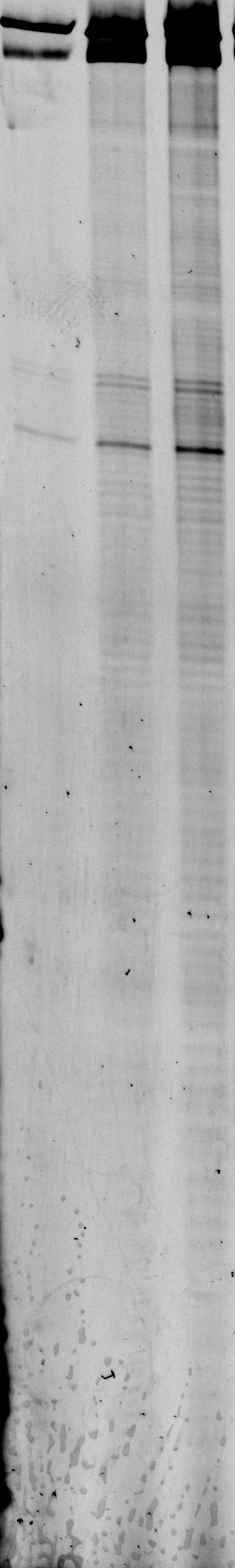

Supplement: Figure 4—source data 1. [file elife-52513-fig4-data1.zip › Figure4-sourcedata-original/Chd1MAPscans/5Dec2018xNucMAP524-Cy3.tif]

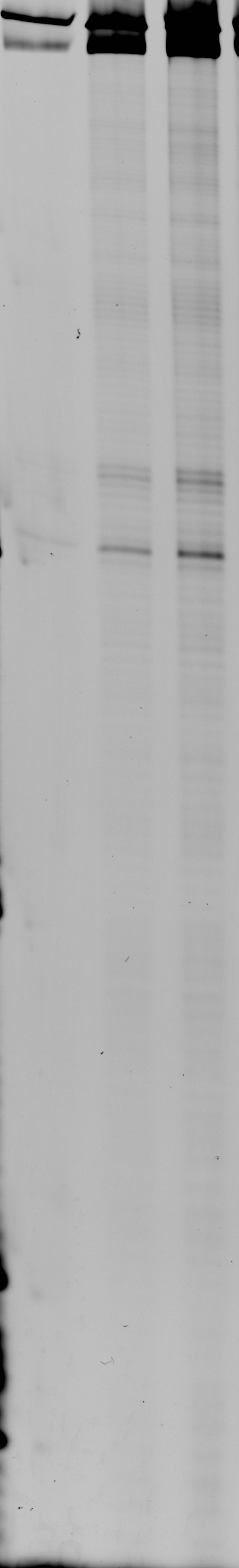

Supplement: Figure 4—source data 1. [file elife-52513-fig4-data1.zip › Figure4-sourcedata-original/Chd1MAPscans/5Dec2018xNucMAP524-Cy5.tif]

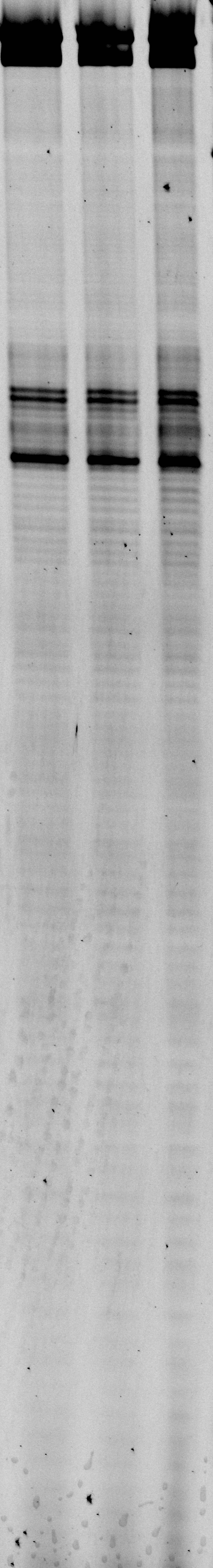

Supplement: Figure 4—source data 1. [file elife-52513-fig4-data1.zip › Figure4-sourcedata-original/Chd1MAPscans/5Dec2018xNucMAP524R804Q-Cy3.tif]

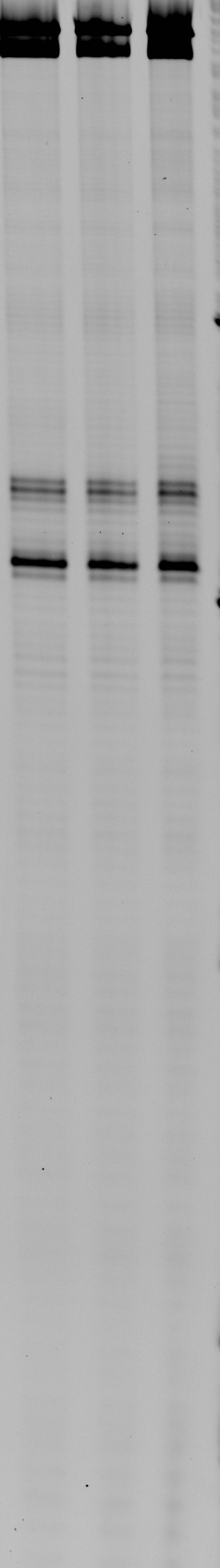

Supplement: Figure 4—source data 1. [file elife-52513-fig4-data1.zip › Figure4-sourcedata-original/Chd1MAPscans/5Dec2018xNucMAP524R804Q-Cy5.tif]

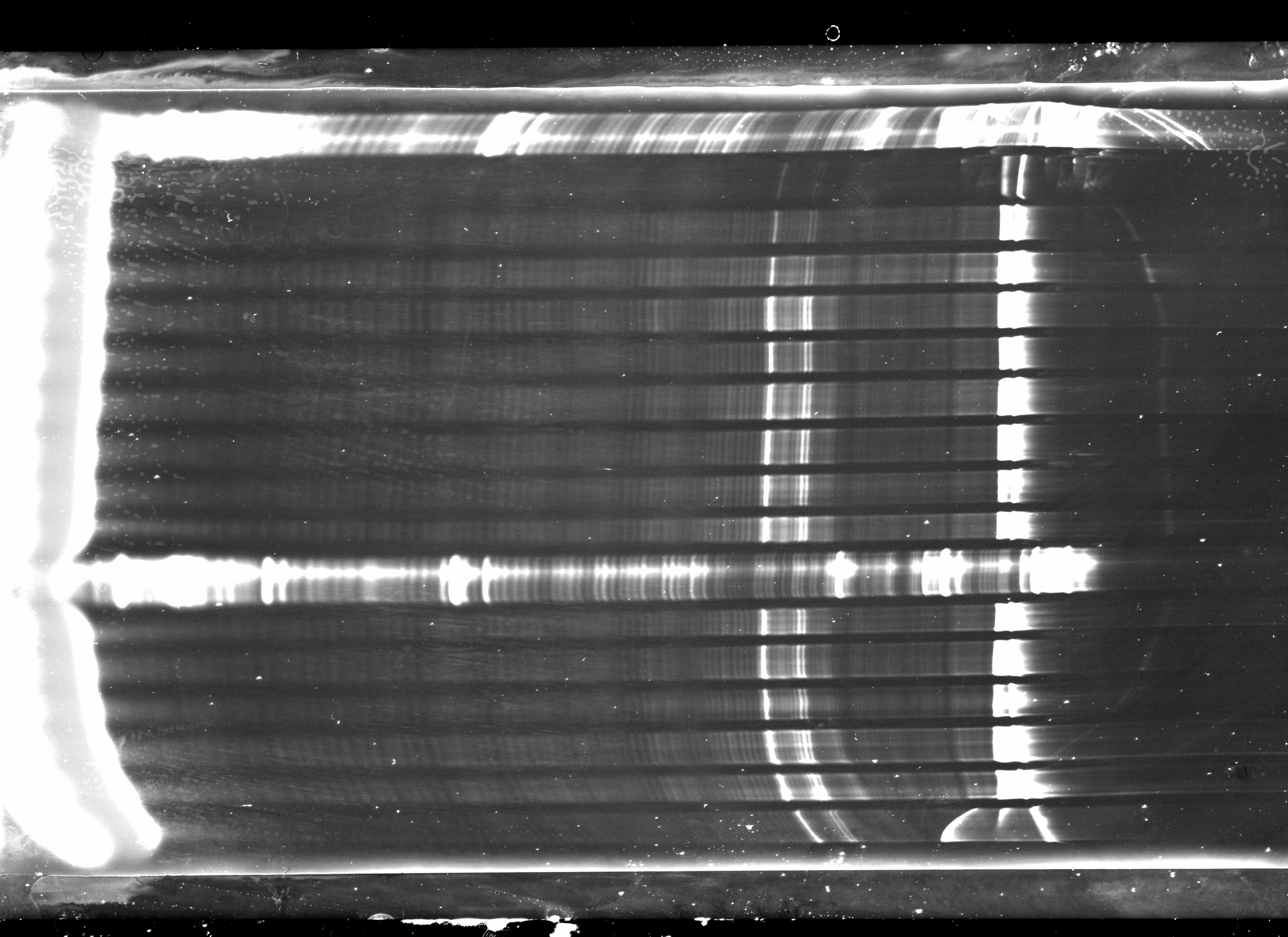

Supplement: Figure 4—source data 1. [file elife-52513-fig4-data1.zip › Figure4-sourcedata-original/Chd1MAPscans/5Dec2018xNucMAP524_804Q_1127_d960d976-Cy3.tif]

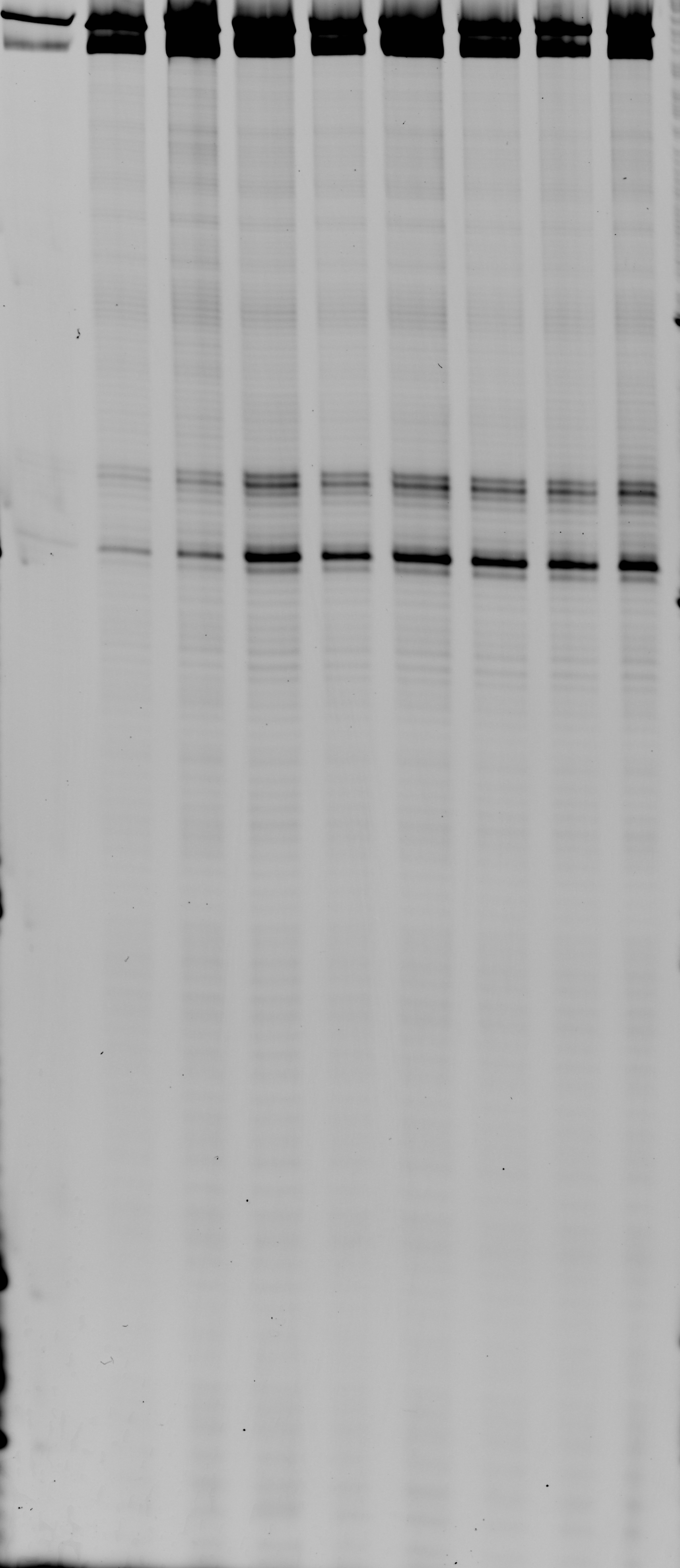

Supplement: Figure 4—source data 1. [file elife-52513-fig4-data1.zip › Figure4-sourcedata-original/Chd1MAPscans/5Dec2018xNucMAP524_804Q_1127_d960d976-Cy5.tif]

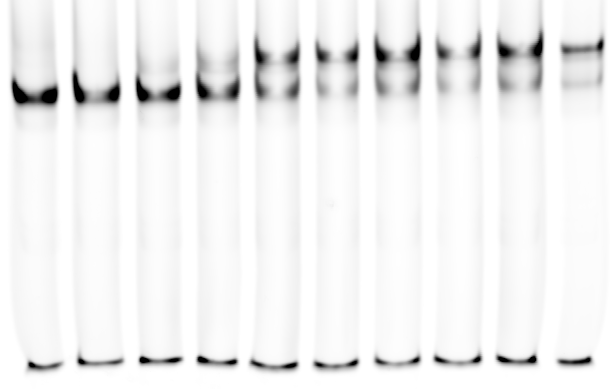

Supplement: Figure 4—figure supplement 1—source data 1. [file elife-52513-fig4-figsupp1-data1.zip › Figure4-figure supplement1-sourcedata-original/18Sept2018Chd1524MAPslideATPconc-[Cy5].tif]

A

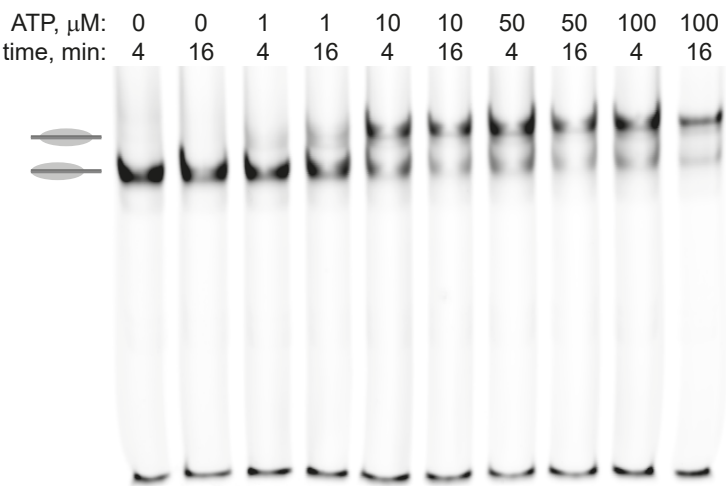

B

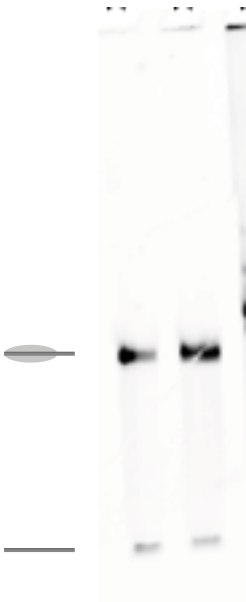

Supplement: Figure 4—figure supplement 1—source data 2. [file elife-52513-fig4-figsupp1-data2.zip › Hughes_et_al_Figure_4_figure_supplement1.pdf]
