# Supplementary material for: Structural characterisation of chromatin remodelling intermediates supports linker DNA-dependent product inhibition as a mechanism for nucleosome spacing
Source: eLife. 2025 Dec 24;14:e52513. doi: 10.7554/eLife.52513 (PMC12834501; doi:10.7554/eLife.52513)
Supplement: Figure 2—source data 2. [file elife-52513-fig2-data2.zip › Figure2_source_data-labelled.pdf]

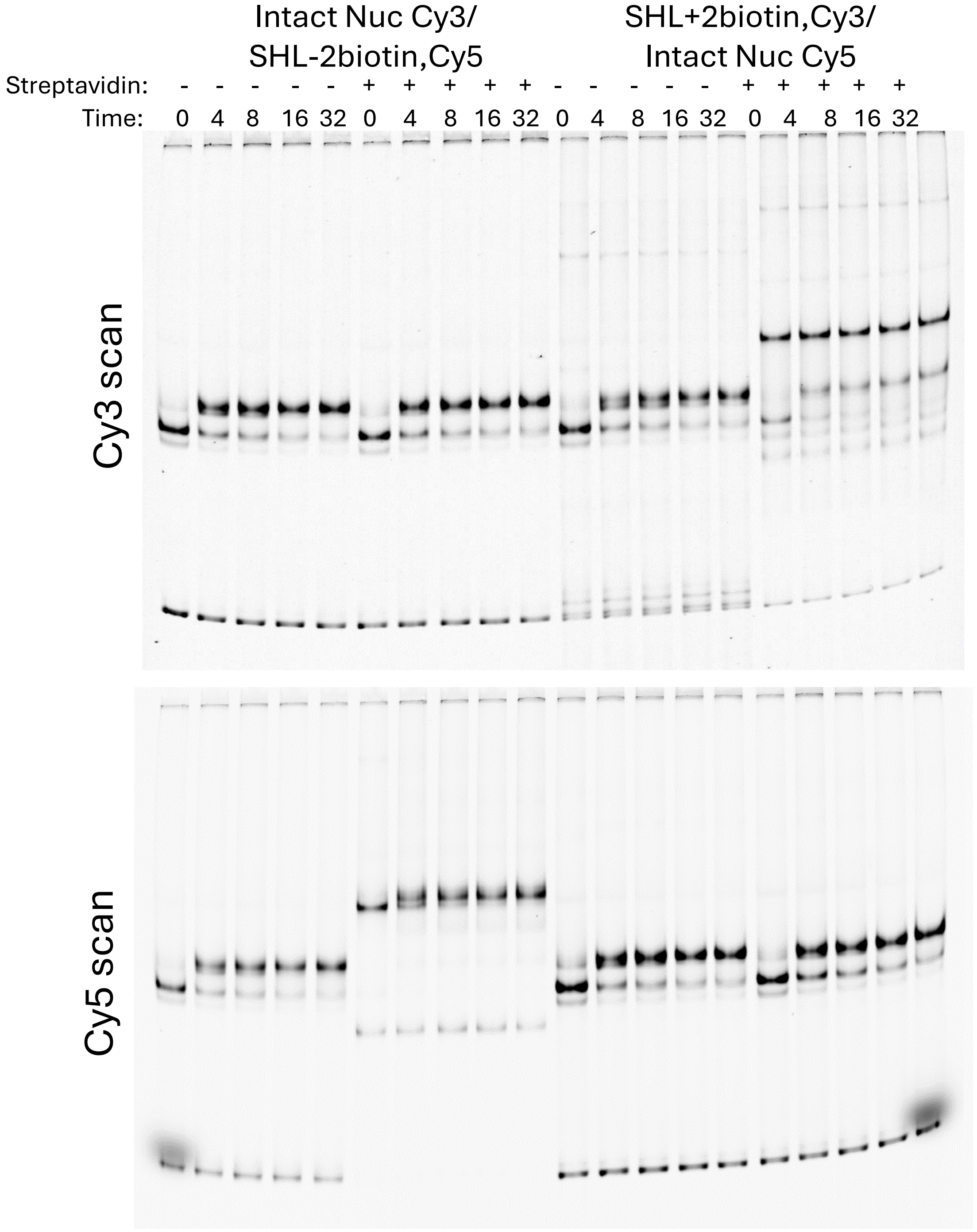

100nM biotin nucleosome
100nM wt nucleosome
1mM ATP
7nM yChd1 1-1305
+/- 1.6uM streptavidin

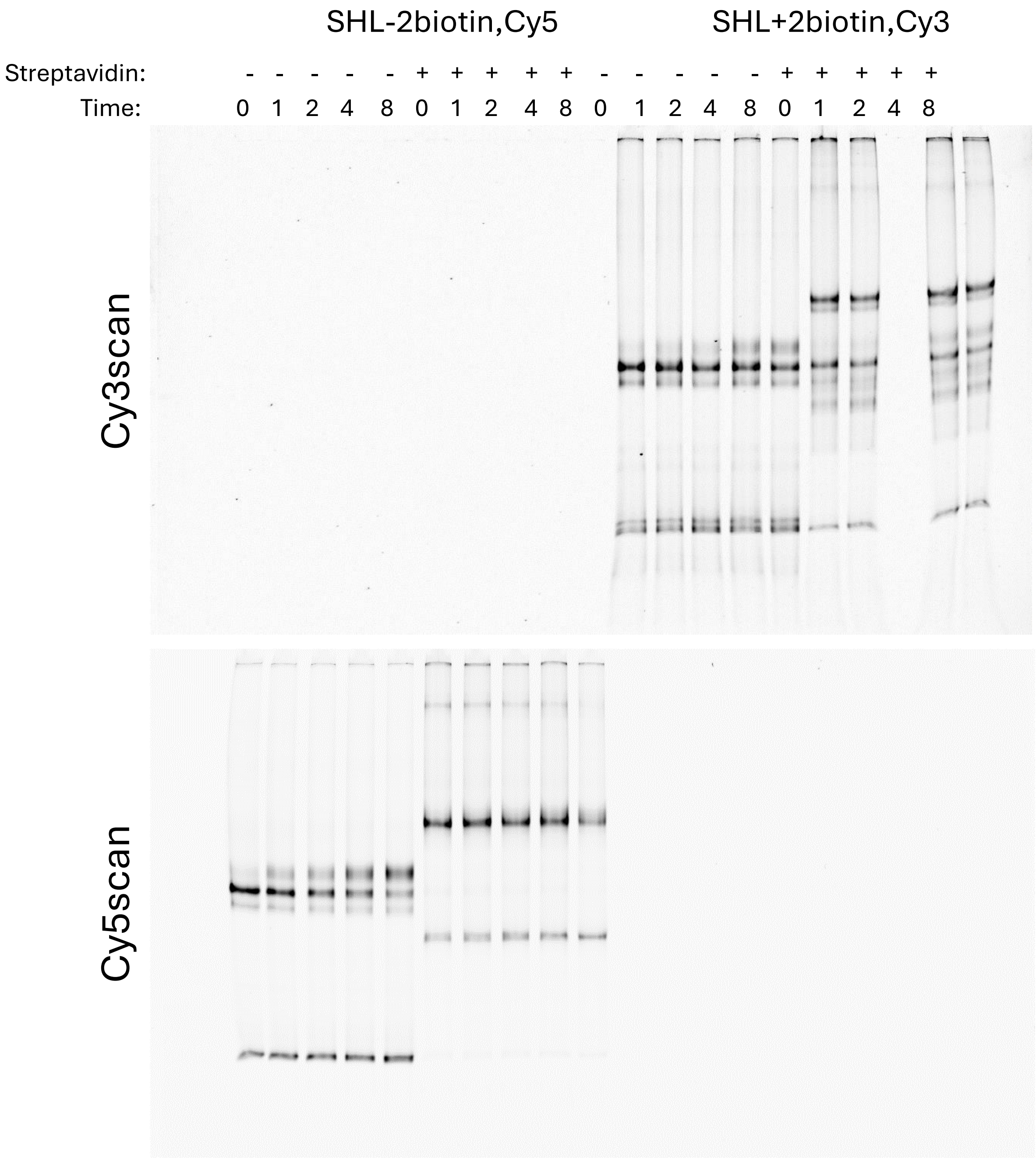

100nM biotin nucleosome
5nM yChd1 1-1305
1mM ATP
+/- 0.4uM streptavidin

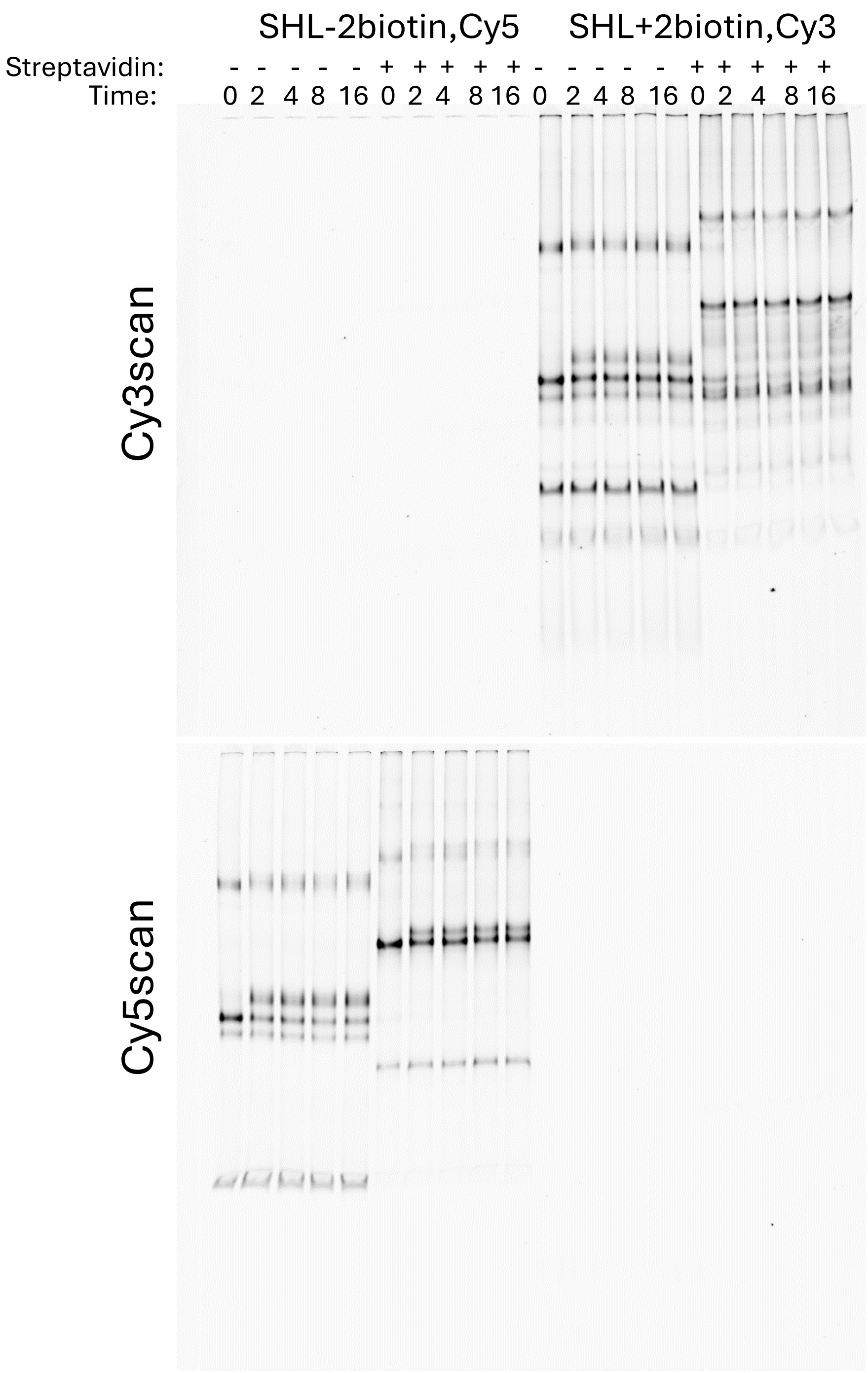

100nM biotin nucleosome
300nM yChd1 1-1305
2.5uM ATP
+/-0.8uM streptavidin

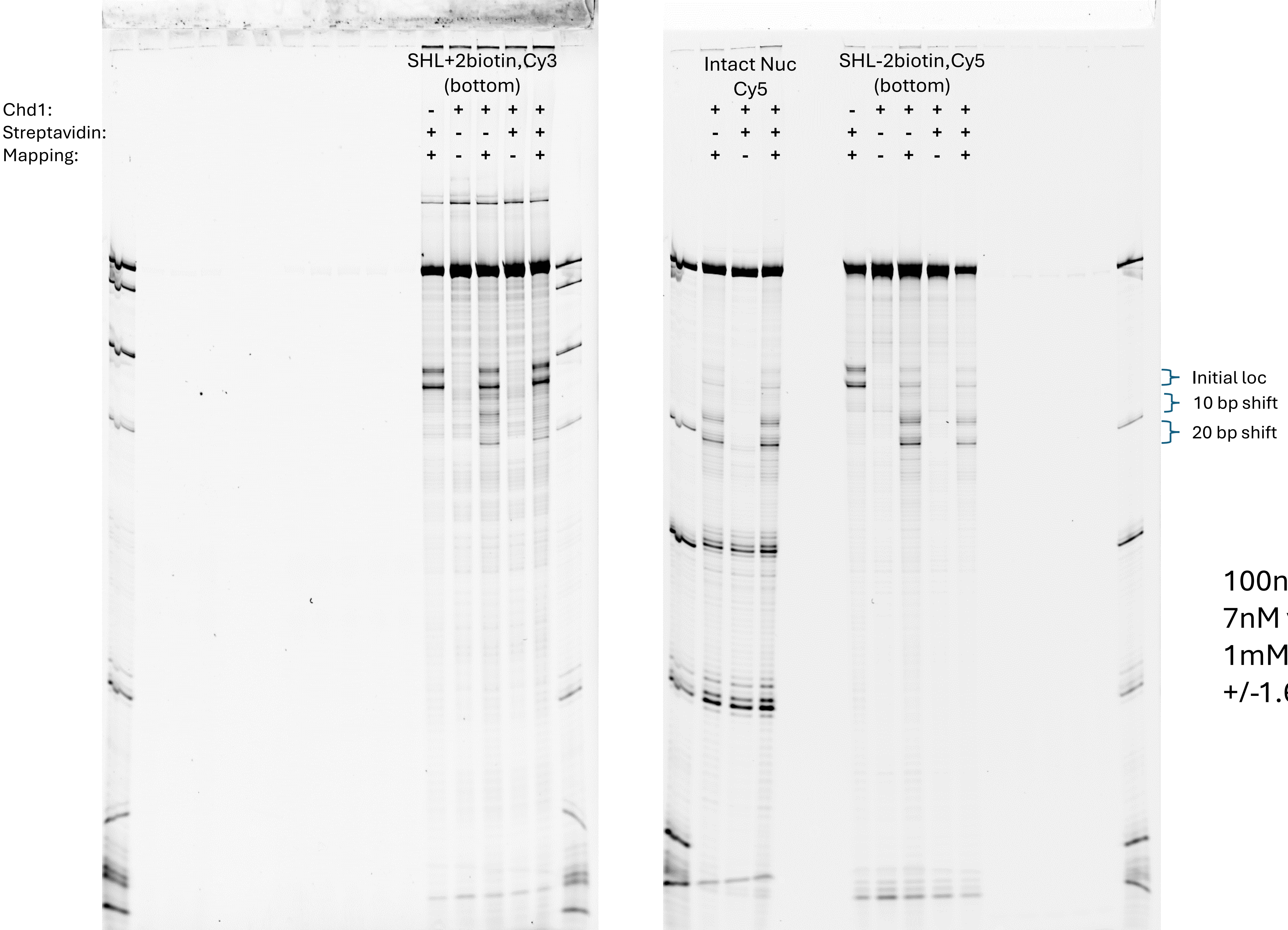

100nM nucleosome
7nM yChd1 1-305
1mM ATP
+/-1.6uM streptavidin

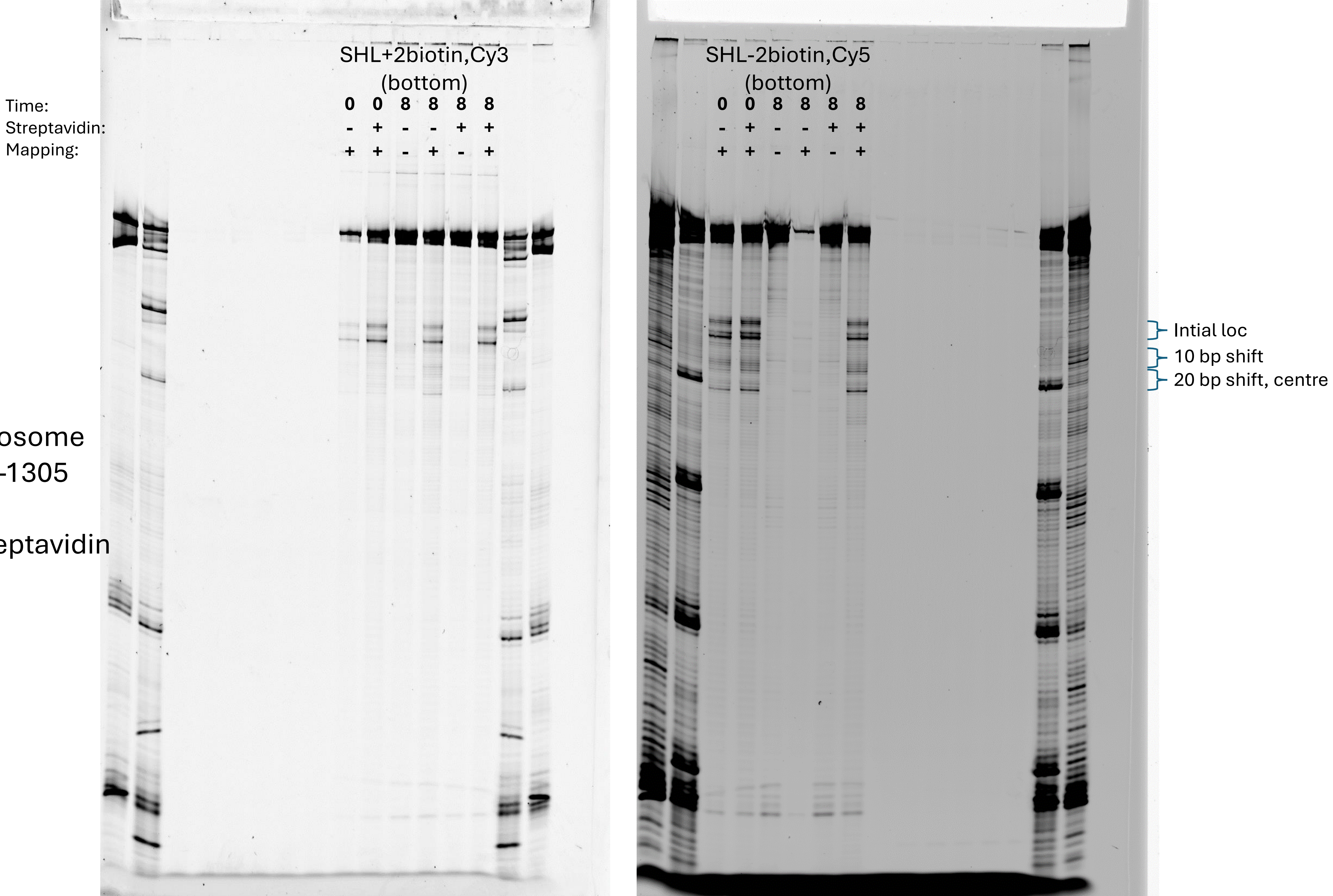

100nM nucleosome
5nM yChd1 1-1305
1mM ATP
+/- 0.4uM streptavidin

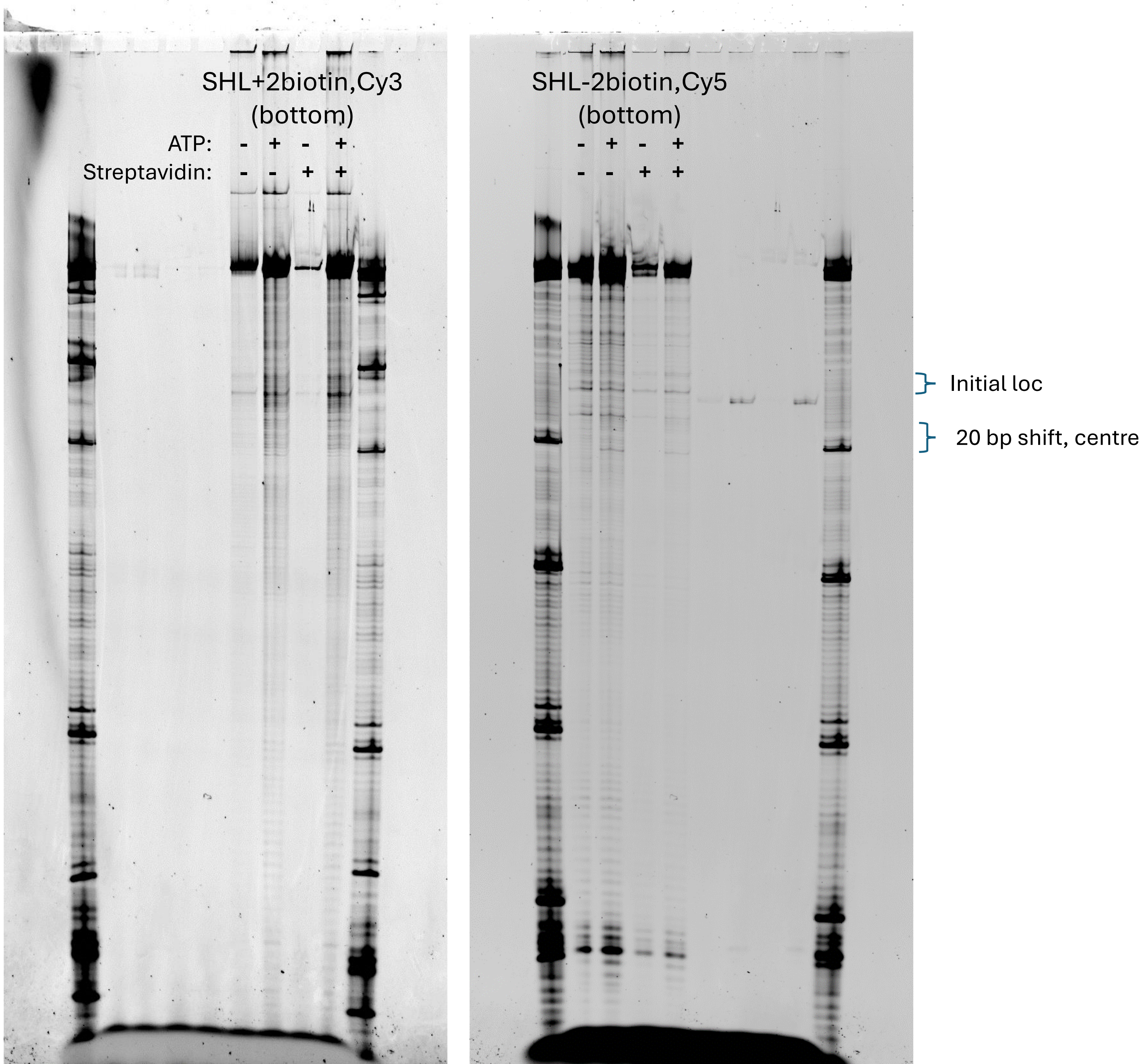

100nM nucleosome
300nM yChd1 1-1305
2.5uM ATP
16 minute sliding @30C
+/-0.8uM streptavidin

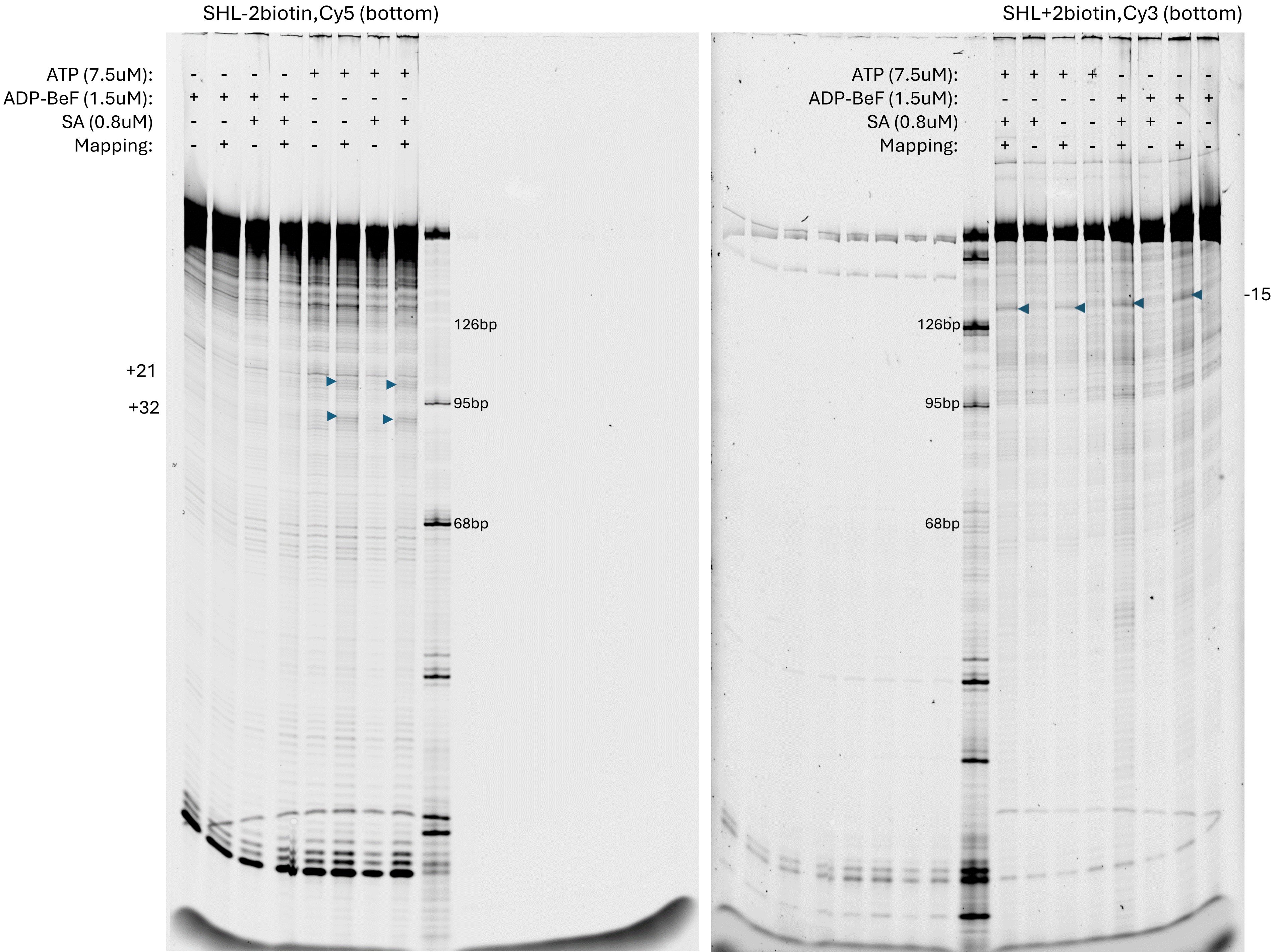
SHL-2biotin,Cy5 (bottom)
ATP (7.5uM): - - - - + + + +
ADP-BeF (1.5uM): + + + + - - - -
SA (0.8uM) - - + + - - + +
Mapping: - + - + - + - +
+21
+32
126bp
95bp
68bp
SHL+2biotin,Cy3 (bottom)
ATP (7.5uM): + + + + - - - -
ADP-BeF (1.5uM): - - - - + + + +
SA (0.8uM) + + - - + + - -
Mapping: + - + - + - + -
-15
126bp
95bp
68bp

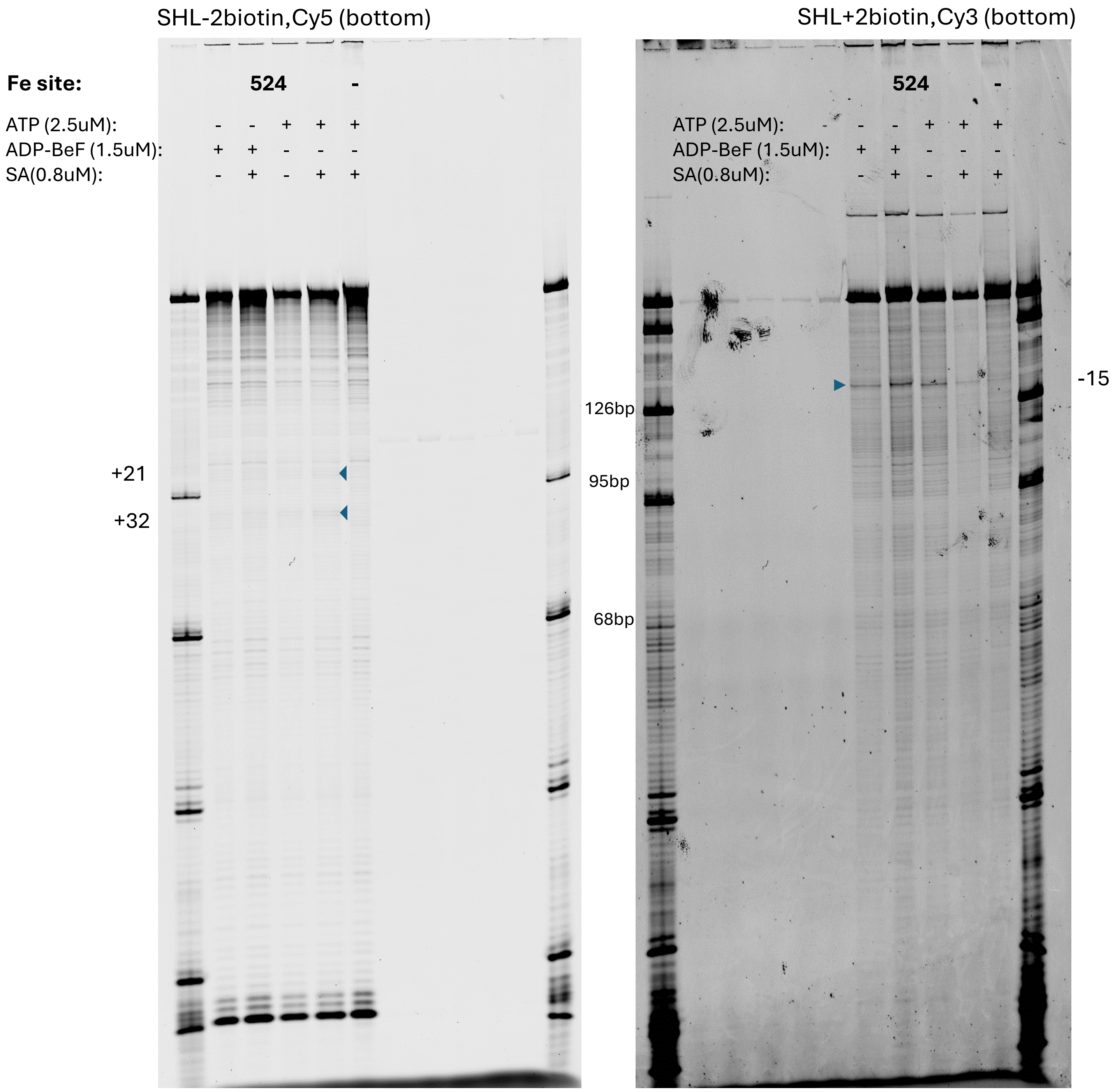
SHL-2biotin,Cy5 (bottom)
SHL+2biotin,Cy3 (bottom)
Fe site:
524
-
ATP (2.5uM):
ADP-BeF (1.5uM):
SA(0.8uM):
+21
+32
126bp
95bp
68bp
-15
