# Supplementary material for: Structural characterisation of chromatin remodelling intermediates supports linker DNA-dependent product inhibition as a mechanism for nucleosome spacing
Source: eLife. 2025 Dec 24;14:e52513. doi: 10.7554/eLife.52513 (PMC12834501; doi:10.7554/eLife.52513)
Supplement: Figure 4—source data 2. [file elife-52513-fig4-data2.zip › Figure4-source_data-labelled.pdf]

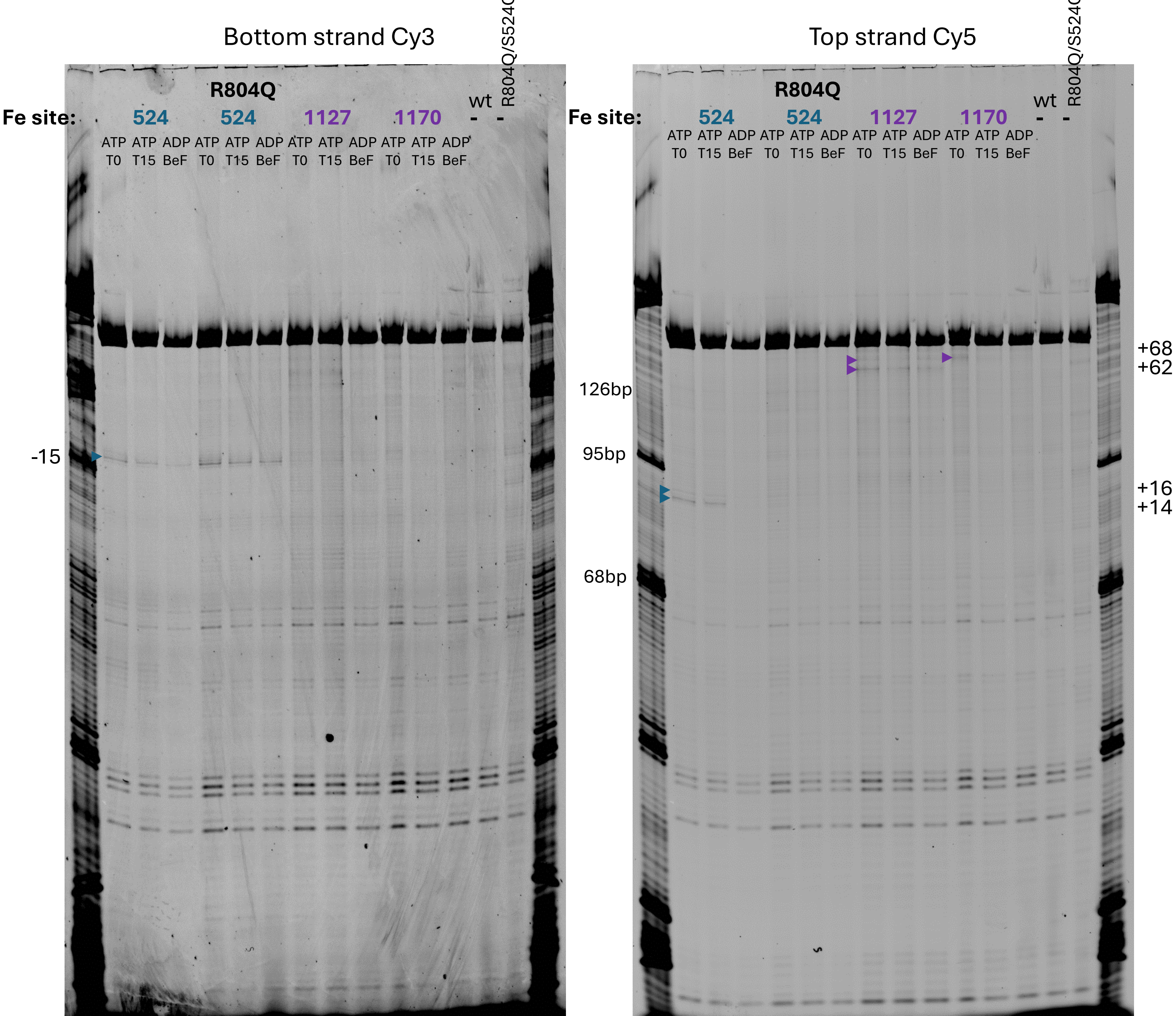
Bottom strand Cy3
Top strand Cy5
R804Q
Fe site:
524
524
1127
1170
wt
R804Q/S524C
ATP T0
ATP T15
ADP BeF
-
-15
126bp
95bp
68bp
+68
+62
+16
+14

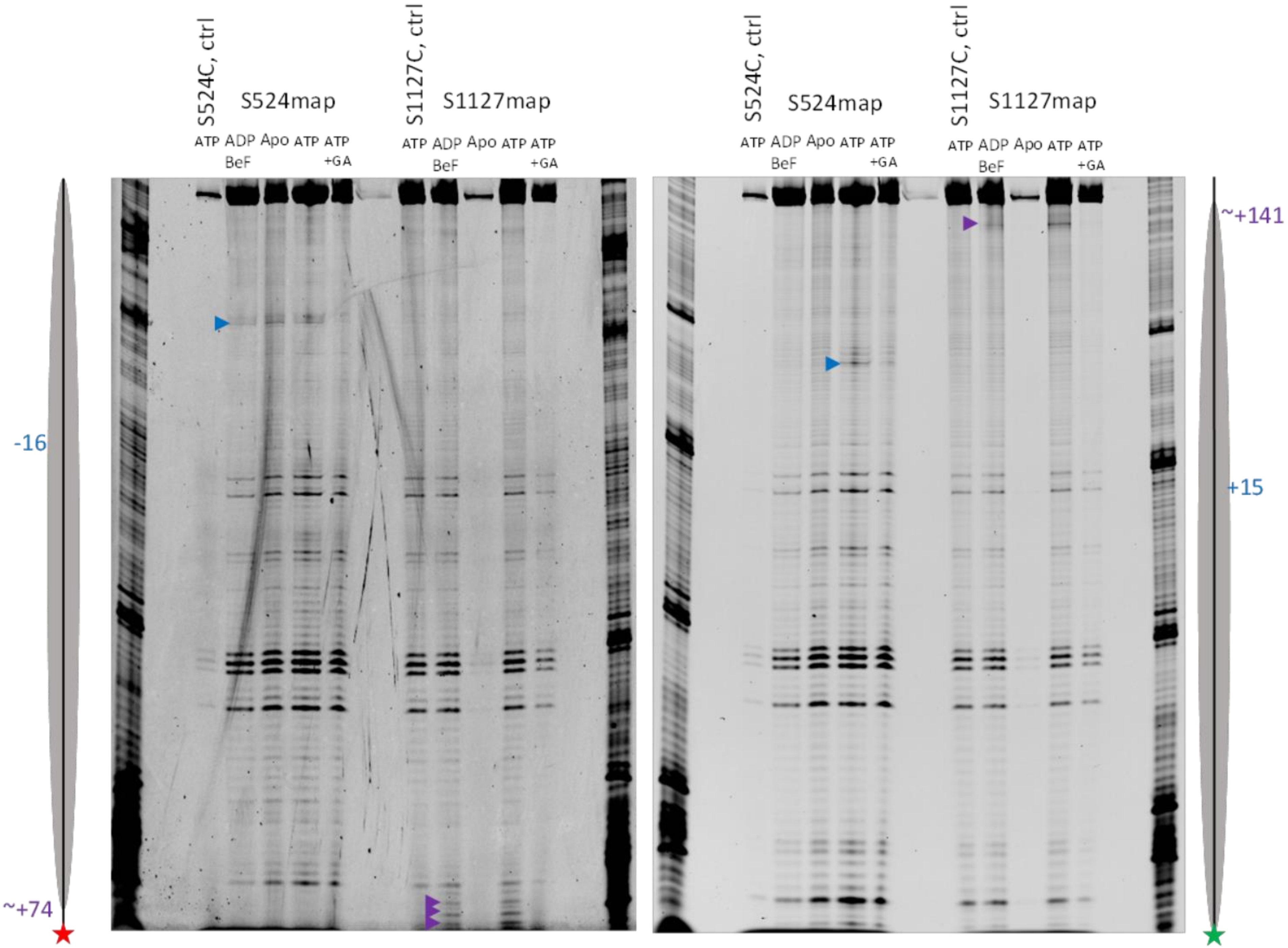
S524C, ctrl
S524map
S1127C, ctrl
S1127map
ATP
ADP BeF
Apo
ATP
ATP +GA
ATP
ADP BeF
Apo
ATP
ATP +GA
S524C, ctrl
S524map
S1127C, ctrl
S1127map
ATP
ADP BeF
Apo
ATP
ATP +GA
ATP
ADP BeF
Apo
ATP
ATP +GA
-16
~+74
~+141
+15

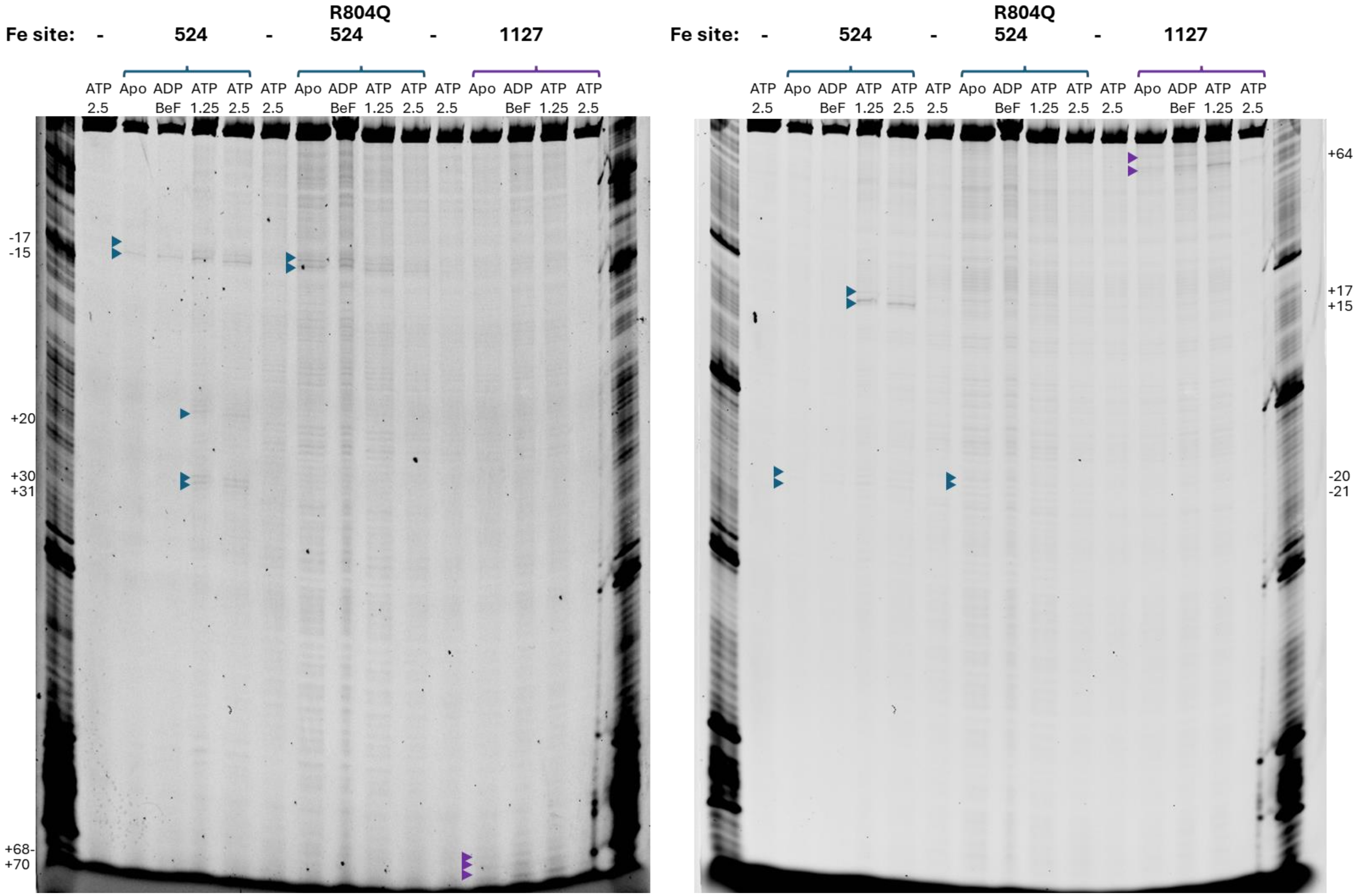

Fe site: - 524 - R804Q 524 - 1127
ATP 2.5 Apo ADP BeF ATP 1.25 ATP 2.5 ATP 2.5 Apo ADP BeF ATP 1.25 ATP 2.5 ATP 2.5 Apo ADP BeF ATP 1.25 ATP 2.5
-17
-15
+20
+30
+31
+68-
+70
Fe site: - 524 - R804Q 524 - 1127
ATP 2.5 Apo ADP BeF ATP 1.25 ATP 2.5 ATP 2.5 Apo ADP BeF ATP 1.25 ATP 2.5 ATP 2.5 Apo ADP BeF ATP 1.25 ATP 2.5
+64
+17
+15
-20
-21

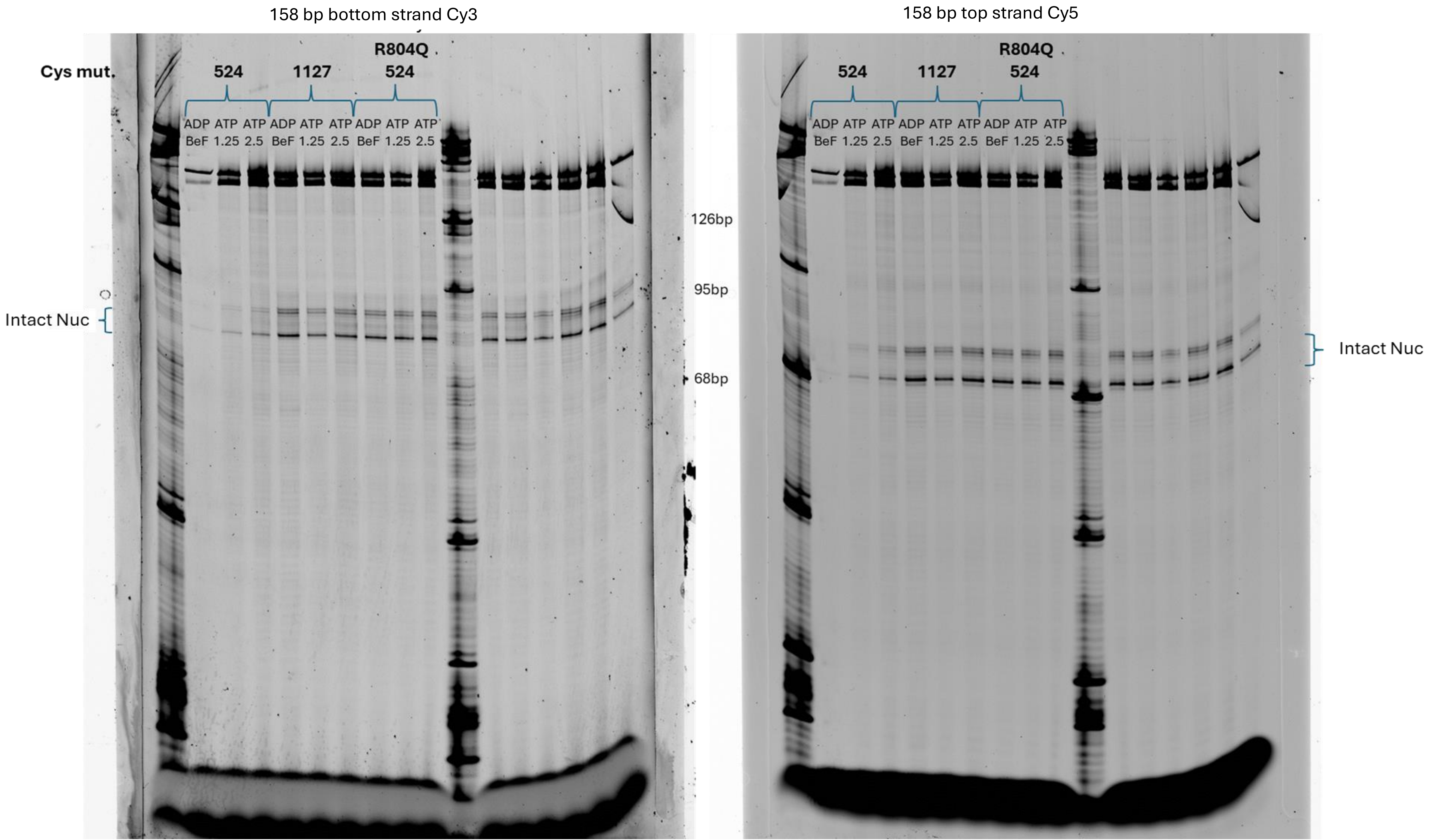
158 bp bottom strand Cy3
Cys mut.
R804Q
524
1127
524
ADP BeF
ATP 1.25
ATP 2.5
ADP BeF
ATP 1.25
ATP 2.5
ADP BeF
ATP 1.25
ATP 2.5
Intact Nuc
126bp
95bp
68bp
158 bp top strand Cy5
R804Q
524
1127
524
ADP BeF
ATP 1.25
ATP 2.5
ADP BeF
ATP 1.25
ATP 2.5
ADP BeF
ATP 1.25
ATP 2.5
Intact Nuc

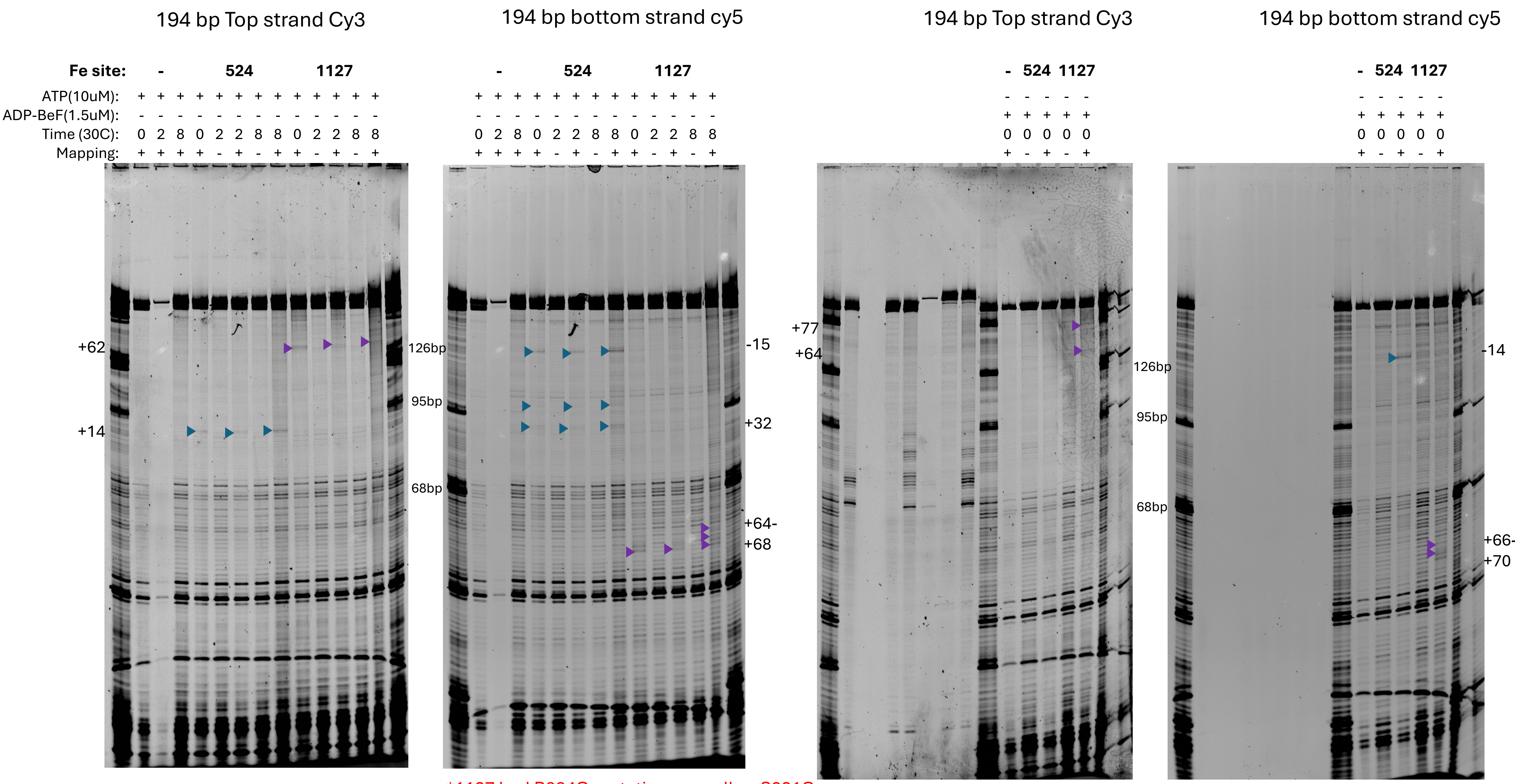

*1127 had R834Q mutation, as well as S221C

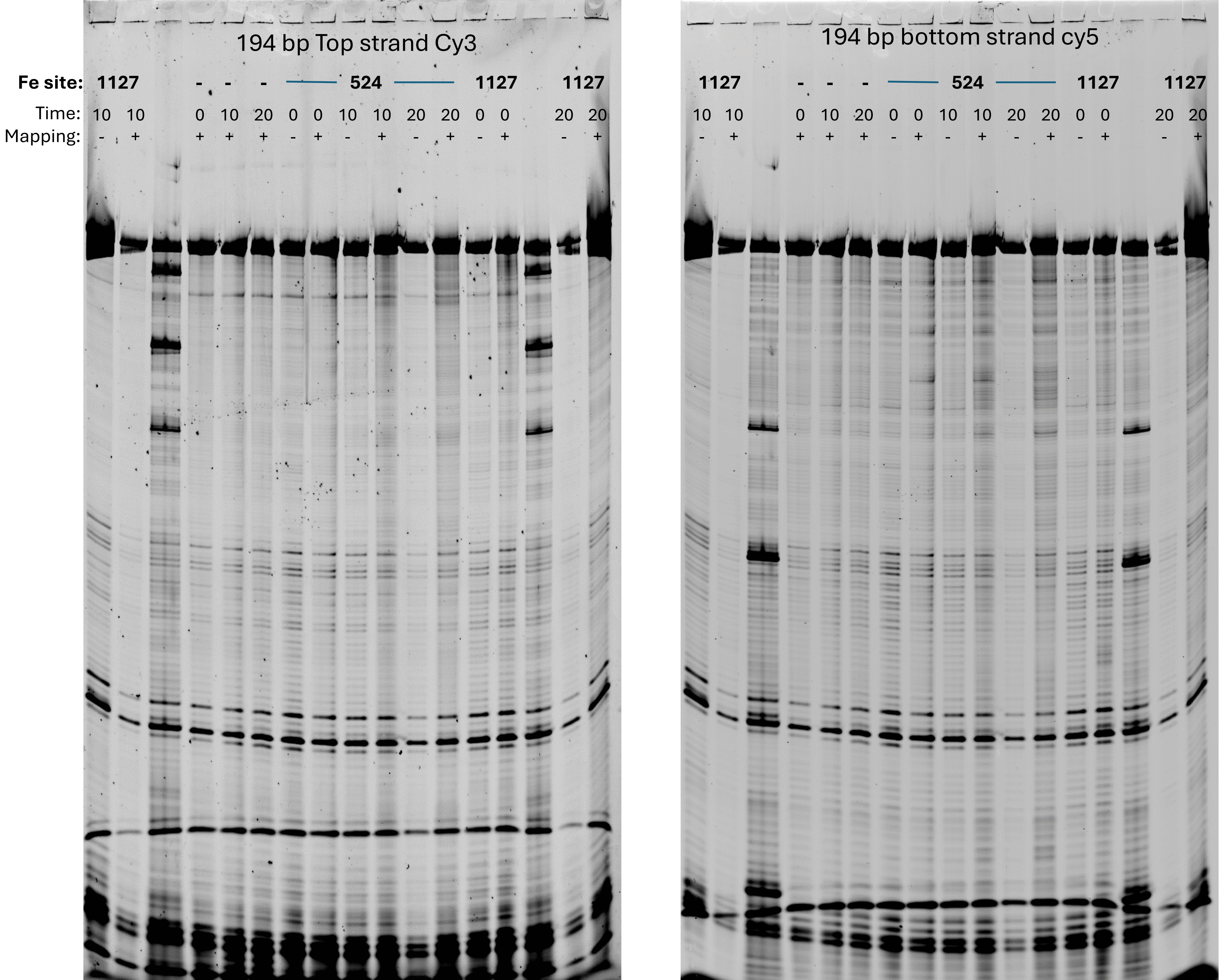

*1127 had R834Q mutation, as well as S221C

194 bp Top strand Cy3

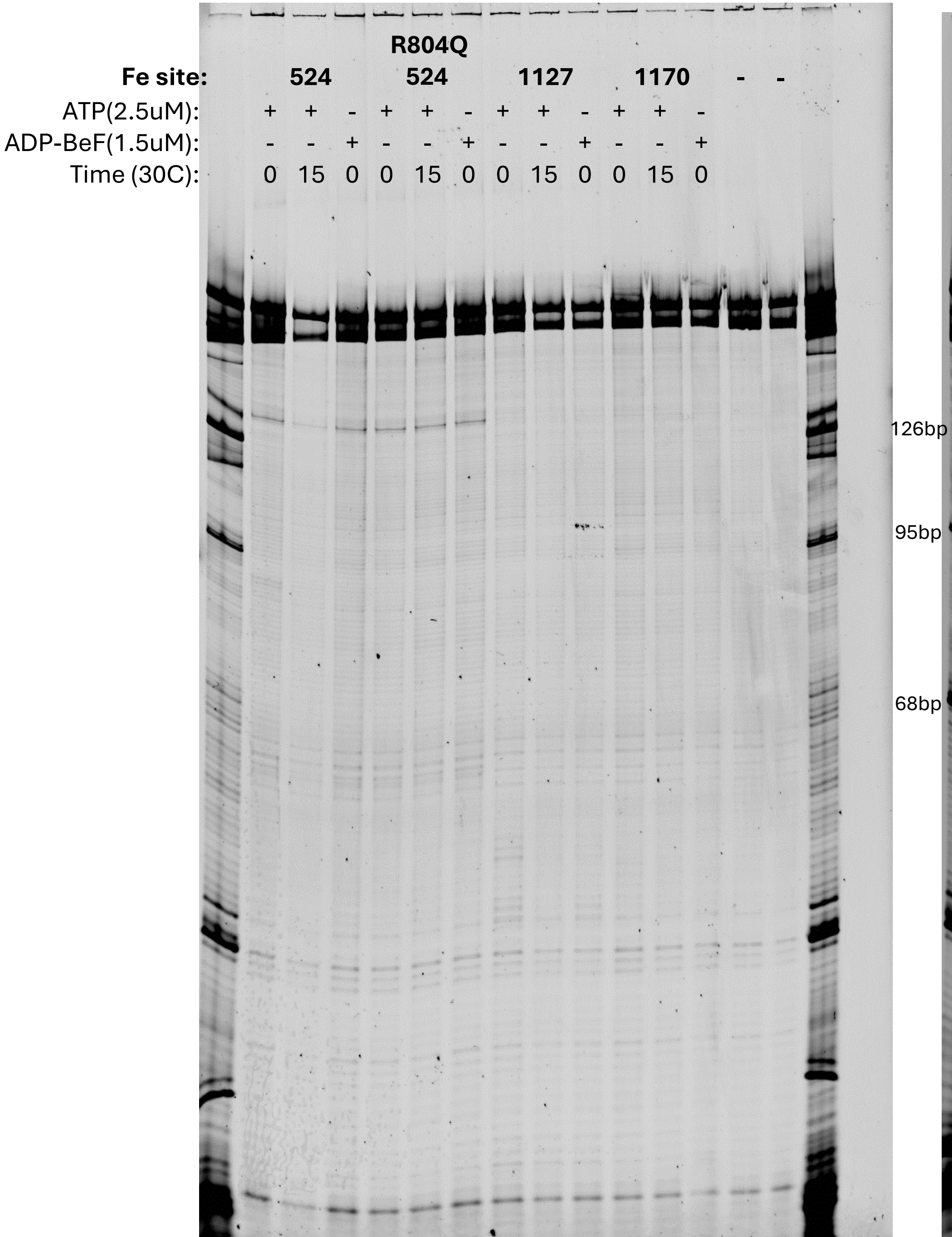

194 bp bottom strand cy5

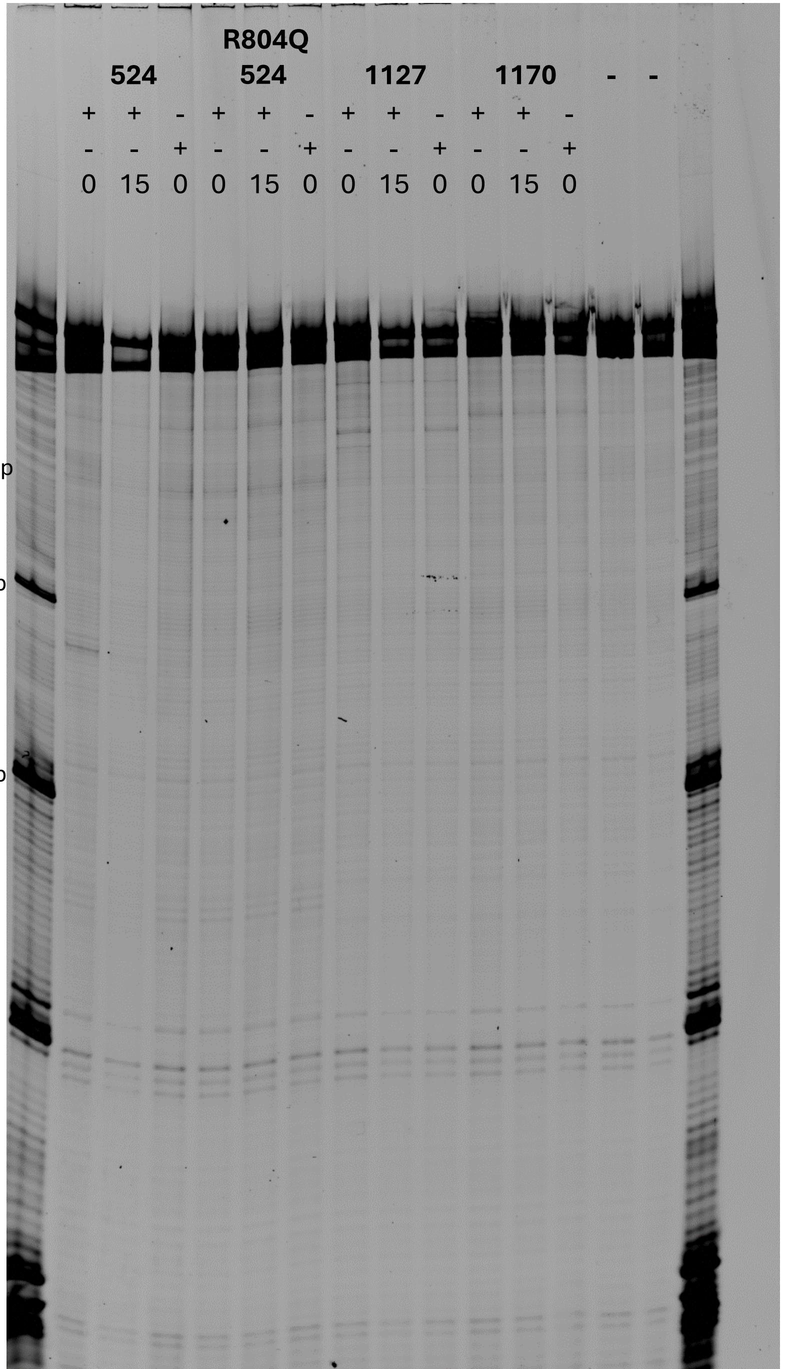

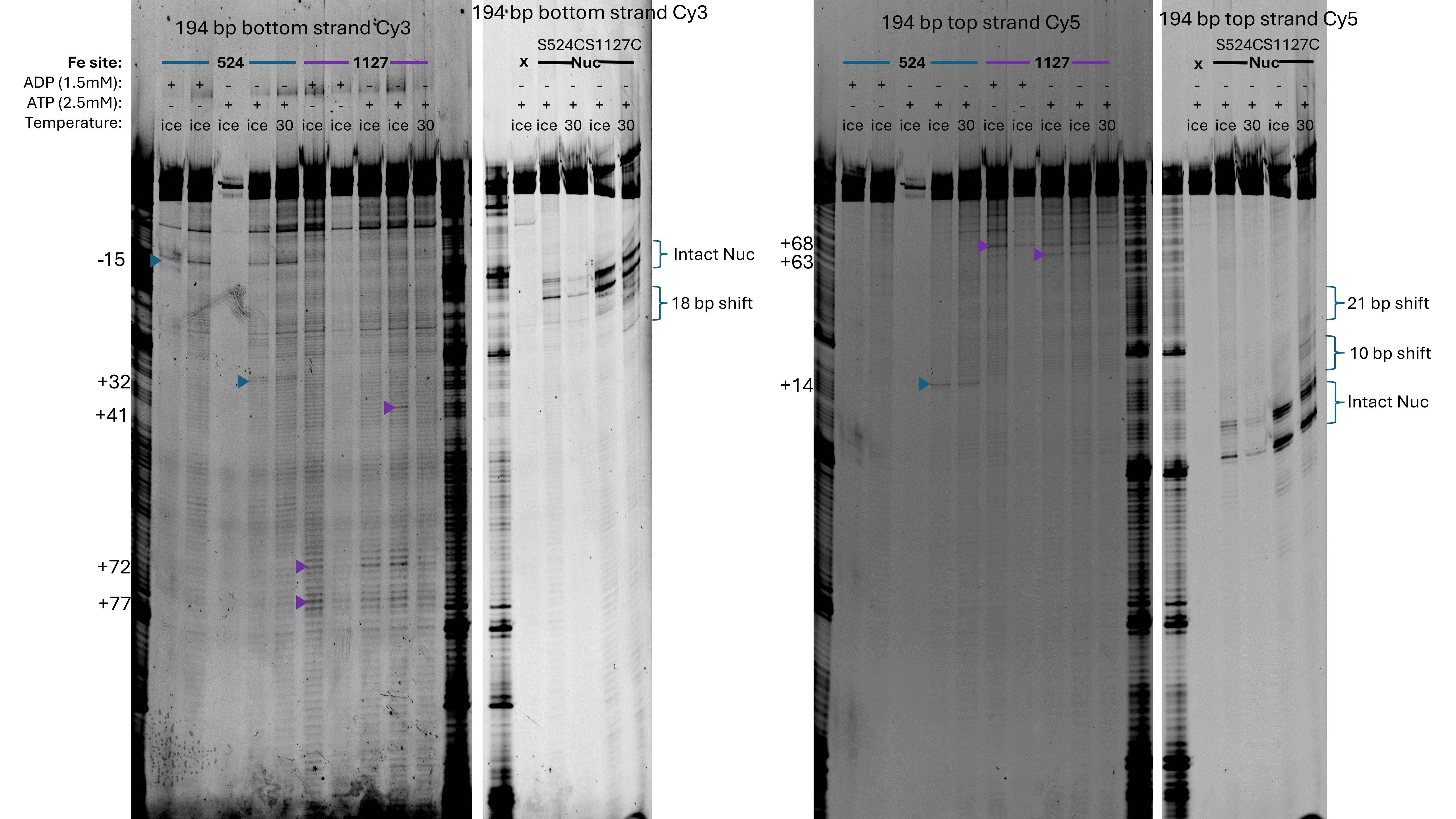
194 bp bottom strand Cy3
Fe site:
524
1127
ADP (1.5mM):
ATP (2.5mM):
Temperature:
+ + - - - + + - - -
- - + + + - - + + +
ice ice ice ice 30 ice ice ice ice 30
-15
+32
+41
+72
+77
194 bp bottom strand Cy3
S524CS1127C
x
Nuc
- - - - -
+ + + + +
ice ice 30 ice 30
Intact Nuc
18 bp shift
194 bp top strand Cy5
524
1127
+ + - - - + + - - -
- - + + + - - + + +
ice ice ice ice 30 ice ice ice ice 30
+68
+63
+14
194 bp top strand Cy5
S524CS1127C
x
Nuc
- - - - -
+ + + + +
ice ice 30 ice 30
21 bp shift
10 bp shift
Intact Nuc

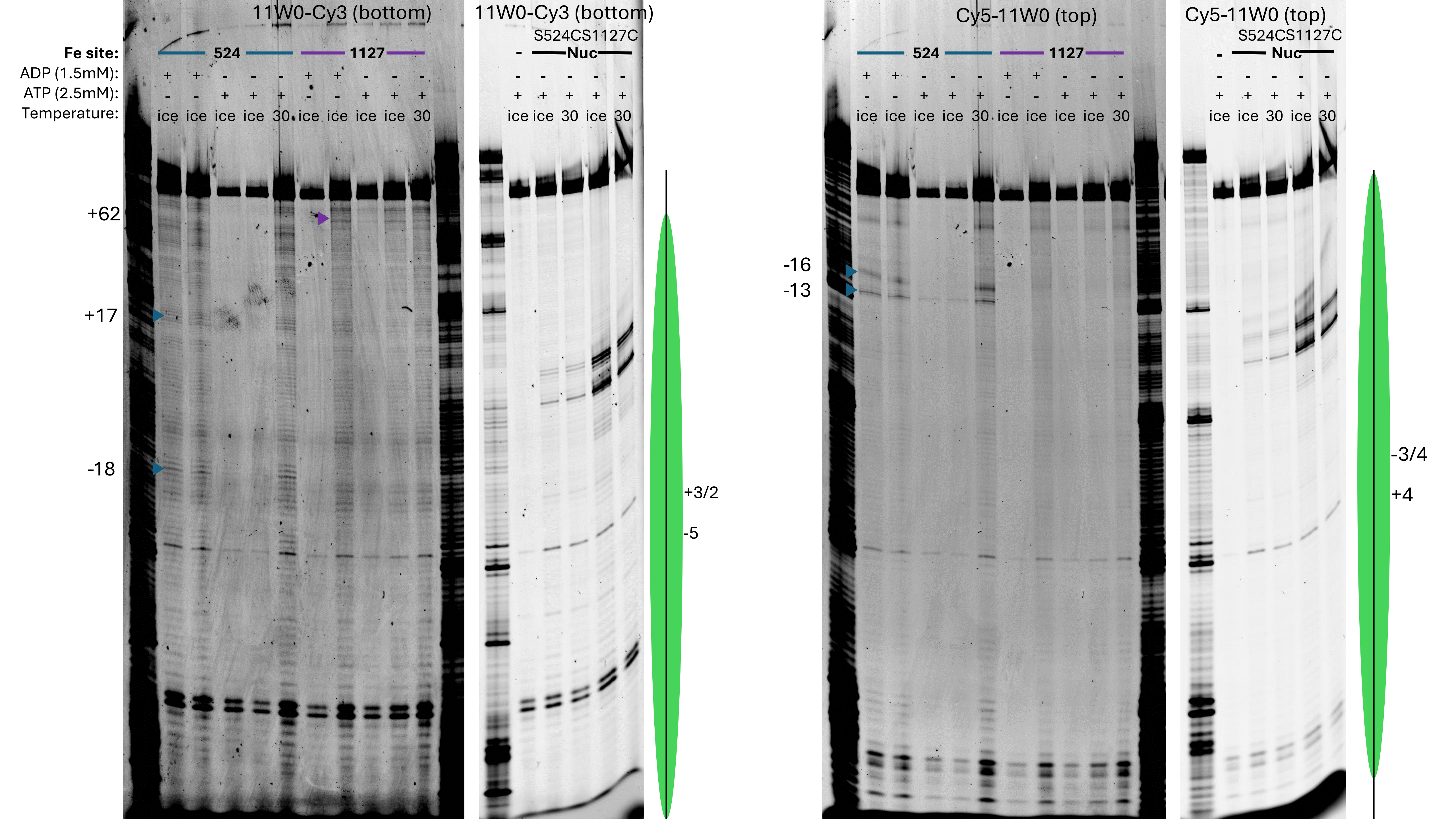
11W0-Cy3 (bottom)
Fe site:
524
1127
ADP (1.5mM):
ATP (2.5mM):
Temperature:
+ + - - - + + - - -
- - + + + - - + + +
ice ice ice ice 30 ice ice ice ice 30
+62
+17
-18
11W0-Cy3 (bottom)
S524CS1127C
- Nuc
- - - - -
+ + + + +
ice ice 30 ice 30
+3/2
-5
Cy5-11W0 (top)
524
1127
+ + - - - + + - - -
- - + + + - - + + +
ice ice ice ice 30 ice ice ice ice 30
-16
-13
Cy5-11W0 (top)
S524CS1127C
- Nuc
- - - - -
+ + + + +
ice ice 30 ice 30
-3/4
+4
